# Spirosalen–scandium catalysts enable the epimerization-tolerant closed-loop circularity of poly(l-lactic acid)

**DOI:** 10.1093/nsr/nwaf416

**Published:** 2025-09-26

**Authors:** Yu-Ting Huang, Hao-Yi Huang, Min Xie, Jia-Hao Cui, Zhi-Jie Wu, Da-Gang Yu, Haifeng Xiang, Zhongzheng Cai, Jian-Bo Zhu

**Affiliations:** National Engineering Laboratory of Eco-Friendly Polymeric Materials (Sichuan), College of Chemistry, Sichuan University, Chengdu 610064, China; National Engineering Laboratory of Eco-Friendly Polymeric Materials (Sichuan), College of Chemistry, Sichuan University, Chengdu 610064, China; National Engineering Laboratory of Eco-Friendly Polymeric Materials (Sichuan), College of Chemistry, Sichuan University, Chengdu 610064, China; National Engineering Laboratory of Eco-Friendly Polymeric Materials (Sichuan), College of Chemistry, Sichuan University, Chengdu 610064, China; National Engineering Laboratory of Eco-Friendly Polymeric Materials (Sichuan), College of Chemistry, Sichuan University, Chengdu 610064, China; Key Laboratory of Green Chemistry & Technology of Ministry of Education, College of Chemistry, Sichuan University, Chengdu 610064, China; Key Laboratory of Green Chemistry & Technology of Ministry of Education, College of Chemistry, Sichuan University, Chengdu 610064, China; National Engineering Laboratory of Eco-Friendly Polymeric Materials (Sichuan), College of Chemistry, Sichuan University, Chengdu 610064, China; National Engineering Laboratory of Eco-Friendly Polymeric Materials (Sichuan), College of Chemistry, Sichuan University, Chengdu 610064, China

**Keywords:** poly(l-lactic acid), chemical recycling, sequence control, stereoselectivity, ring-opening polymerization

## Abstract

Although poly(l-lactic acid) (PLLA) has attracted broad interest as a sustainable plastic, its chemical recycling to a monomer has been an elusive endeavour. A major obstacle for this process is that the epimerization of l-lactide (l-LA) to *meso*-LA has led to a loss of the original material performance of PLLA. Our robust spirosalen–scandium complexes (Sc) showcased outstanding heteroselectivity towards ring-opening polymerization of *rac*-LA and syndioselectivity for the polymerization of *meso*-LA. Herein we report a strategy to circumvent the inevitable epimerization by exploiting an Sc catalytic system, which promoted stereoselective and sequence-controlled polymerization of l-LA in the presence of *meso*-LA and gave rise to the stereogradient block PLA P(*meso*-LA-*grad*-LLA) without compromising the material properties of the original PLLA. This epimerization-tolerant strategy for chemical recycling of PLLA provides an innovative pathway for the next generation of chemical recyclable polymers.

## Teaser

A new catalytic system was developed to promote stereoselective and sequence-controlled polymerization of L-LA in presence of meso-LA, delivering stereogradient PLA without compromising the material properties of the original PLLA.

## INTRODUCTION

**Figure 1.**
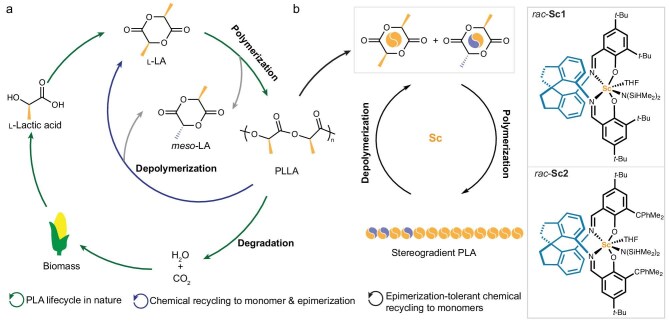
PLA lifecycle. (a) Chemical recycling to monomer. (b) Epimerization-tolerant chemical recycling to monomers.

The massive production and consumption of petroleum-based synthetic polymers have raised severe environmental and economic problems due to their poor degradability and limited resources [1,2]. To address the plastic pollution and resource crisis, the development of sustainable polymers derived from renewable feedstocks and possessing chemical recyclability has attracted growing attention [3–10]. Poly(l-lactic acid) (PLLA), as one of the most prevalent and commercial bioplastics worldwide, has emerged as a promising alternative to the traditional petroleum-based plastics owing to its renewable resource, inherent degradability and comparable material performance to polyolefins [11]. Nowadays, PLLA is fabricated from l-lactic acid, which is produced via fermentation of renewable natural resources such as corn starch and sugarcane (Fig. 1a) [12]. l-Lactic acid can undergo polycondensation to form oligomers, which are subsequentially depolymerized to the cyclic dimer l-lactide (l-LA). The production of PLLA is via ring-opening polymerization (ROP) of l-LA. Due to the biodegradability of PLLA, it provides several end-of-life options including landfill, incineration, industrial composting and recycling [13]. It should be noted that PLLA products are still stable in soil and difficult to degrade under natural environmental conditions [14]. Moreover, the composting process could lead to no material recovery and low compost quality. Instead, mechanical and chemical recycling of PLLA is considered as a desirable sustainable process. Despite the feasibility of mechanical recycling, PLLA has a narrow processing temperature window, due to its low thermal stability and high melting transition temperature, which limits the applicability of mechanical recycling. In fact, hydrolysis of PLLA has been reported, but this treatment forms lactic acid or alkyl lactates as product, which requires extra processing steps to access the original monomer l-LA [15–18]. Consequently, chemical recycling of PLLA to l-LA via ring-closing depolymerization (RCD) appears to present an ideal, circular polymer economy with the closed-loop life cycle of PLLA (Fig. 1a).

To date, only a few catalytic systems including Zn(OAc)_2_, ZnCl_2_ and Sn(Oct)_2_ or additives have been established to efficiently depolymerize PLLA to l-LA [19–23]. However, the occurrence of inevitable epimerization during the depolymerization process in these systems led to the generation of *meso*-LA in the recycled l-LA, and increasing the chemical recycling contributed to the accumulation of *meso*-LA content in the recycled l-LA. In fact, current commercial PLLA products generally are prepared via ROP of l-LA accompanied by a trace amount of *meso*-LA [24,25]. As a result, the contamination of *meso*-LA would disturb the stereoregularity of the recycled PLLA. Generally, the high stereoregularity of PLLA is highly demanded for high-performance material properties. PLLA with decreased stereoregularity might display significantly reduced crystallinity and other physical and chemical properties. In this regard, the development of new chemical recycling approaches for PLLA without a loss in material properties is still a crucial and formidable challenge.

Despite many successful reports of new catalytic systems for stereoselective ROP of LA [26–44], these catalytic systems are still facing challenges of the trade-off between the reactivity and selectivity. Recently, we have designed chiral metal complexes supported by a spirosalen scaffold as catalysts for the stereoselective ROP of *rac*-lactones with excellent reactivity and selectivity simultaneously [45–47]. These evolutionary findings prompted us to further explore this spirosalen scaffold for stereoselective polymerization of *rac*-LA and *meso*-LA and depolymerization of PLLA. We speculated that these spirosalen–scandium complexes (Sc) adopted a *cis*-α configuration with a bulky environment around the active metal center, which could accommodate a proximal binding mode for the coordinated monomer and the last inserted chain-ends to amplify the influence of the chain-end control and promote stereoselective polymerization for LA. Notably, these robust Sc showcased outstanding heteroselectivity towards ROP of *rac*-LA and syndioselectivity for the polymerization of *meso*-LA. Based on the high reactivity and syndioselectivity of Sc catalysts towards the ROP of *meso*-LA, we exploited these Sc catalysts to develop an epimerization-tolerant chemical recycling strategy for PLLA to circumvent the undesired epimerization of l-LA accompanied by chemical recycling of PLLA (Fig. 1b). As proof of our concept, copolymerization of a mixture of l-LA and *meso*-LA by Sc was investigated to provide the stereogradient block PLA P(*meso*-LA-*grad*-LLA) with competent material properties. Ultimately, an epimerization-tolerant chemical recycling of PLLA was executed to recover l-LA containing 1%–2% *meso*-LA from the first recycling. The recovered LA monomers (*meso*-LA:l-LA = 1:99 or 2:98) underwent repolymerization to afford the recycled P(*meso*-LA-*grad*-LLA) with melting transition temperature (*T*_m_) values of 146°C–154°C, demonstrating the feasibility for chemical recycling of PLLA with tolerance of epimerization by our Sc catalysts.

## RESULTS AND DISCUSSION

### Stereocontrolled ROP of LA

**Figure 2.**
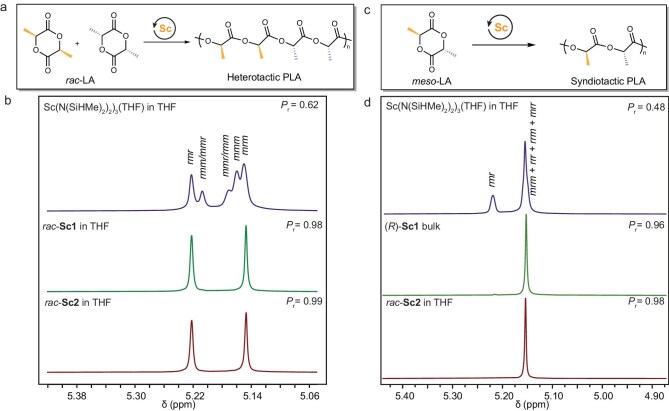
Stereocontrolled ROP of LA. (a) Scheme of the stereocontrolled ROP of *rac-*LA. (b) Homonuclear decoupled ^1^H NMR spectra overlay of heterotactic PLA products with *P*_r_ values from 0.62 to 0.99. (c) Scheme of the stereocontrolled ROP of *meso*-LA. (d) Homonuclear decoupled ^1^H NMR spectra overlay of syndiotactic PLA products with *P*_r_ values from 0.48 to 0.98.

Complexes *rac*-Sc1 and *rac*-Sc2 were initially examined as the catalysts for the ROP of *rac*-LA at a [*rac*-LA]:[catalyst]:[initiator] ratio of 200:1:1 (Fig. 2a). Impressively, the *rac*-Sc1-mediated polymerization approached 93% conversion within 1.2 h at room temperature, demonstrating a turnover frequency (TOF) of 155 h^−1^ (Table 1, Run 1). A heterotactic PLA with a *P*_r_ (probability of racemic linkages between monomer units) value of 0.98 was obtained, indicative of excellent stereoselectivity of *rac*-Sc1. The bulkier scandium complex *rac*-Sc2 exhibited perfect heteroselectivity, albeit with a sacrifice of the catalytic activity (Table 1, Run 2). Size exclusion chromatography (SEC) analysis revealed that the resulting PLA samples displayed number-average molecular weights (*M*_n_ values) of 25.2–28.7 kDa with narrow dispersity (*Đ* < 1.05), which was consistent with the theoretical values (*M*_n, Calcd_) calculated from the initial [LA]:[I] ratios and monomer conversions.

**Table 1.** Ring-opening polymerization results of LA^a^.

Run	LA	Catalyst (Cat)	[LA]:[Cat]:[I]	Time (h)	Conversion^b^ (%)	TOF^c^ (h^−1^)	*M*_n,Calcd_^d^ (kDa)	*M*_n_^e^ (kDa)	*Đ* ^e^	*P*_r_^f^
1	*rac*-LA	*rac*-Sc1	200:1:1	1.2	93	155	26.9	28.7	1.04	0.98
2	*rac*-LA	*rac*-Sc2	200:1:1	19.5	91	9	26.3	25.2	1.05	0.99
3	*meso*-LA	(*R*)-Sc1	200:1:1	1.5	98	131	28.3	26.1	1.05	0.96
4	*meso*-LA	(*R*)-Sc1	500:1:1	6	>99	82	71.4	54.2	1.06	0.96
5	*meso*-LA	(*R*)-Sc1	1000:1:1	24	96	40	138	101	1.05	0.95
6^g^	*meso*-LA	(*R*)-Sc1	2000:1:1	8 min	49	7350	141	71.2	1.06	0.96
7	*meso*-LA	(*S*)-Sc1	200:1:1	1.2	98	163	28.3	25.2	1.06	0.95
8	*meso*-LA	*rac*-Sc1	200:1:1	1.5	96	128	27.8	33.0	1.06	0.95
9	*meso*-LA	*rac*-Sc2	50:1:1	2.5	99	20	7.32	8.3	1.06	0.98
10	*meso*-LA	*rac*-Sc2	200:1:1	11	93	17	26.9	25.6	1.04	0.98
11	*meso*-LA	*rac*-Sc2	500:1:1	24	76	16	54.8	42.7	1.04	0.98
12^g^	*meso*-LA	*rac*-Sc2	1000:1:1	0.5	54	1080	77.9	28.3	1.04	0.90

a Condition: LA = 150 mg (1.04 mmol), [LA] = 1.0 M, *p*-tolylmethanol as the initiator (I), THF as the solvent, room temperature. ^b^Monomer conversion measured by ^1^H NMR of the quenched solution. ^c^TOF = {[LA]/[Cat] × Conv. (%)}/time (h). ^d^Calculated from [LA]_0_/[I]_0_ × Conv. × MW_LA_ + MW_Initiator_. ^e^*M*_n_ and *Đ* (*M*_w_/*M*_n_) determined by SEC at 40°C in THF. ^f^*P*_r_ is the probability of *rac* linkage determined by ^1^H NMR spectroscopy. ^g^Reaction temperature: 55°C, neat.

It’s still challenging to prepare syndiotactic PLA via stereoselective ROP of *meso*-LA [48]. To date, only a few catalytic systems have been found to enable the preparation of highly syndiotactic PLA with limited *M*_n_ values due to their moderate catalytic activity [42,49,50]. The excellent heteroselectivity of *rac*-Sc complexes towards ROP of *rac*-LA motivated us to investigate their catalytic performance for the polymerization of *meso*-LA (Fig. 2c). Under a similar polymerization condition, (*R*)-Sc1 promoted the polymerization of *meso*-LA with a TOF of 131 h^−1^ and yielded a syndiotactic PLA product with a *P*_r_ value of 0.96 (Table 1, Run 3). Increasing the [*meso*-LA]:[(*R*)-Sc1]:[I] ratio from 200:1:1 to 1000:1:1 contributed to an increase in *M*_n_ from 26.1 to 101 kDa without a decrease of *P*_r_ (Table 1, Runs 3–5). Remarkably, bulk polymerization with a [*meso*-LA]:[(*R*)-Sc1]:[I] ratio of 2000:1:1 at 55°C rapidly reached 49% monomer conversion in 8 min, demonstrating a TOF of 7350 h^−1^ (Table 1, Run 6). The resulting syndiotactic PLA showed a *P*_r_ value of 0.96 and an *M*_n_ of 71.2 kDa with a *Đ* of 1.06, maintaining the excellent stereocontrol at increased temperature. In addition, (*S*)-Sc1 and *rac*-Sc1 showcased a similar reactivity and stereoselectivity pattern to (*R*)-Sc1, delivering syndiotactic PLA products with a *P*_r_ value of 0.95 (Table 1, Runs 7–8). Solvent screening revealed that tetrahydrofuran (THF) could act as a coordinating solvent and compete with monomers for binding to the catalytic metal center, thereby leading to the decrease in catalytic activity (Table S1, Runs 1–3). *rac*-Sc2 was next evaluated for the ROP of *meso*-LA, which facilitated stereocontrolled polymerization and afforded syndiotactic PLA products with an improved *P*_r_ of 0.98 (Table 1, Runs 9–11). This is one of the highest *P*_r_ records to date for the syndioselective ROP of *meso*-LA. In comparison to Sc1, Sc2 equipped with bulkier ligand exhibited higher stereoselectivity albeit with a decrease in catalytic activity. To boost the catalytic activity of *rac*-Sc2, bulk polymerization with a [*meso*-LA]:[*rac*-Sc2]:[I] ratio of 1000:1:1 at 55°C was carried out. As expected, *rac*-Sc2 exhibited a significant increase of the catalytic activity with a TOF of 1080 h^−1^, despite a slight decrease of syndioselectivity (Table 1, Run 12). The stereochemical microstructure of these synthesized PLA samples was determined by homonuclear decoupled ^1^H NMR spectroscopy. As shown in Fig. 2d, the degrees of syndiotacticity of the produced PLA samples could be calculated based on the integral ratios of the methine region. Collectively, this Sc system allowed access of syndiotactic PLA with a *P*_r_ value of up to 0.98 and an *M*_n_ up to 101 kDa.

**Figure 3.**
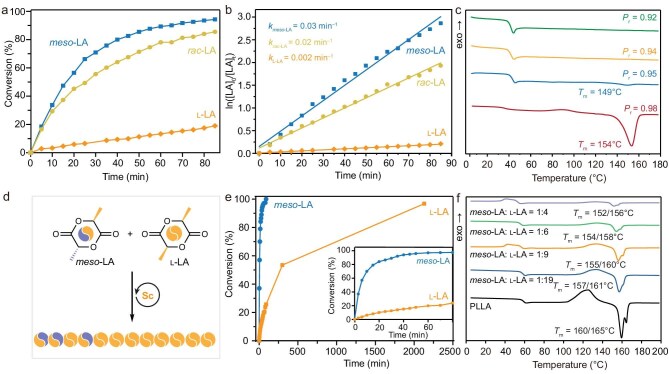
ROP study of *meso*-LA and copolymerization with l-LA. (a) Time–conversion plots of *meso*-LA, l-LA and *rac*-LA for (*S*)-Sc1-meidated polymerization, 1.0 M in THF, room temperature, [LA]:[(*S*)-Sc1]:[I] = 200:1:1. (b) Kinetic plot for (*S*)-Sc1-mediated polymerization of *meso*-LA, l-LA and *rac*-LA. (c) DSC curves of PLA samples with *P*_r_ values of 0.92, 0.94, 0.95 and 0.98. (d) Copolymerization of *meso*-LA and l-LA. (e) Time-conversion plots of copolymerization *meso*-LA and l-LA. (f) DSC curves of PLA samples with *meso-*LA:l-LA = 1:4, 1:6, 1:9 and 1:19.

To better elucidate the stereocontrolled mechanism of this Sc catalyst system, kinetic study of the ROP of *rac*-LA and *meso*-LA was performed by monitoring the monomer conversions over reaction time through ^1^H NMR spectroscopy (Fig. 3a and Fig. S1). Linear correlations between ln([LA]_0_/[LA]) and reaction time were acquired for all the kinetic plots, indicative of first-order kinetics (Fig. 3b and Fig. S3). Specifically, (*S*)-Sc1, (*R*)-Sc1 and *rac*-Sc1 accommodated similar l-LA consumption rates with *k*_l__-LA_ of ∼0.002 min^−1^, while they showed a *rac*-LA consumption rate (*k_rac_*_-LA_) of ∼0.02 min^−1^. Consequently, a relative *k_rac_*_-LA_:*k*_l__-LA_ ratio of 10:1 suggested a chain-end control mechanism for heteroselectivity. In line with the kinetic study for the ROP of *rac*-LA, the polymerization of *meso*-LA by (*R*)-Sc1, and *rac*-Sc1 also showed a linear dependence of ln([*meso*-LA]_0_/[*meso*-LA]) on reaction time with a slope (*k_meso_*_-LA_) of ∼0.03 min^−1^ (Fig. 3b), which provided corroborative evidence for the syndioselectivity of Sc1 via chain-end control. A relative *k_meso_*_-LA_:*k*_l__-LA_ ratio of 15:1 suggested distinct polymerization kinetics for l*-*LA and *meso*-LA.

A series of syndiotactic PLA samples with different *P*_r_ values were examined by thermal gravimetric analysis (TGA) and differential scanning calorimetry (DSC) to explore the effect of stereoregularity of the PLA products on their thermal properties. The thermal decomposition performance of these PLA samples was characterized by the 5% mass loss decomposition temperature (*T*_d_), and these polymers displayed a range of *T*_d_ from 247°C to 260°C (Figs S40–S43). As shown in Fig. 3c, DSC curves of these PLA samples revealed that only negligible melting transition on the second heating scan was observed for syndiotactic PLA samples having *P*_r_ values of 0.92–0.95, indicative of a slow crystallization process. Syndiotactic PLA with a *P*_r_ value of 0.95 displayed a *T*_m_ of 149°C with a tiny heat of fusion (*∆H*_m_ = 3.30 J/g). An increase of *P*_r_ to 0.98 for the syndiotactic PLA sample led to the appearance of a clear *T*_m_ of 154°C with a *∆H*_m_ of 43.6 J/g, manifesting the significance of stereoregularity. This *T*_m_ value (154°C) was comparable to those (152°C–153°C) reported for syndiotactic PLA with a *P*_r_ of 0.96 [42,49–51], further confirming the high *P*_r_ value of our sample.

### Ring-opening copolymerization (ROCOP) of *meso*-LA and l-LA

Inspired by the high reactivity and syndioselectivity of Sc catalysts towards the ROP of *meso*-LA, we anticipated exploiting this catalytic system to prepare the stereogradient block PLA P(*meso*-LA-*grad*-LLA) via ROCOP of a mixture of l-LA and *meso*-LA, which would possess competent material performance in comparison to PLLA (Fig. 3d). Consequently, this strategy could enable compatibility for the epimerization of l-LA during polymerization and serve as a promising solution to chemical recycling of PLLA. To verify our proposal, ROCOP of *meso*-LA and l-LA was next investigated as a model study. Initially, the ROP of l-LA with an [l-LA]:[(*S*)-Sc1]:[I] ratio of 200:1:1 was carried out to provide PLLA with an *M*_n_ of 43.2 kDa and a *Đ* of 1.04. As a reference, this isotactic PLLA displayed *T*_m_ values of 160°C–165°C (Fig. 3f). ROCOP of a mixture of l-LA and *meso*-LA in various feed ratios of 99:1, 19:1, 9:1, 6:1 and 4:1 was then performed. To probe the copolymerization process, the polymerization mixture of l-LA and *meso*-LA in a 9:1 ratio was monitored by ^1^H NMR analysis. It was found that *meso*-LA was rapidly consumed, with quantitative conversion in 60 min, while only 20% l-LA consumption was observed at this point. Further extending the reaction time to 25 h led to 93% conversion of l-LA, yielding the final PLA, the stereogradient block product P(*meso*-LA-*grad*-LLA) (Fig. 3e). A series of copolymer P(*meso*-LA-*grad*-LLA) with *meso*-LA compositions from 1% to 20% were prepared accordingly, demonstrating *M*_n_ values of 37.0–42.7 kDa with narrow dispersity (*Đ* < 1.1) (Table 2, Entries 2–6). Additionally, increasing the [LA]:[(*S*)-Sc1]:[I] ratio from 200:1:1 to 600:1:1 gave rise to P(*meso*-LA-*grad*-LLA) samples with improved *M*_n_ values (Table 2, Entries 7–10).

**Table 2.** ROCOP results of *meso*-LA and l-LA^a^.

				Conversion^b^ (%)					Second heating crystallization		Second heating melting	
Entry	[LA]/[(*S*)-Sc1]/[I]	l-LA/*meso*-LA	Time (h)	*meso*-LA	l-LA	*M*_n,Calcd_^c^ (kDa)	*M*_n_^d^ (kDa)	*Đ* ^d^	*T*_c_^e^ (°C)	Δ*H*_c_^e^ (J/g)	*T*_m_^e^ (°C)	Δ*H*_m_^e^ (J/g)
1	200:1:1	100:0	21		94	27.2	43.2	1.04	125	41.6	160–165	39.6
2	200:1:1	99:1	22	>99	83	24.0	38.0	1.09	133	17.5	157	19.3
3	200:1:1	19:1	37	>99	96	27.8	42.7	1.05	134	18.4	157–161	20.2
4	200:1:1	9:1	25	>99	93	27.2	41.0	1.04	134	13.7	155–160	12.8
5	200:1:1	6:1	22.5	>99	88	26.0	37.0	1.06	134	5.8	154–158	6.9
6	200:1:1	4:1	21	>99	92	27.2	37.4	1.07	133	2.4	152–156	4.0
7	300:1:1	9:1	37	>99	84	32.3	57.1	1.07	138	4.2	159–162	5.9
8	400:1:1	9:1	16.5	>99	77	45.6	63.1	1.05	135	1.0	158	1.9
9	600:1:1	9:1	16.5	>99	72	74.9	81.5	1.06	135	0.5	161	1.5
10	600:1:1	19:1	22	>99	82	71.8	74.9	1.07	135	3.1	161	3.9

a Reaction conditions: LA = 150 mg, [LA] = 1.0 M, *p*-tolylmethanol as the initiator (I), THF as the solvent, room temperature. ^b^Monomer conversion measured by ^1^H NMR of the quenched solution. ^c^Calculated from [LA]_0_/[I]_0_ × Conv. × MW_LA_ + MW_Initiator_. ^d^*M*_n_ and *Ð* (*M*_w_/*M*_n_) determined by SEC at 40°C in THF. ^e^*T*_c_ and *T*_m_ were measured by DSC with the cooling and heating rate of 10°C min^−1^ for all samples.

A DSC study was next conducted to gain further insights into the *meso*-LA composition influence on the thermal performance of these stereogradient block PLA samples. It was found that the increment of the *meso*-LA composition in the resulting P(*meso*-LA-*grad*-LLA) from 5% to 20% led to a slight reduction of *T*_m_ values from 157°C–161°C to 152°C–156°C (Fig. 3f). Relative to the isotactic PLLA having *T*_m_ values of 160°C–165°C, these P(*meso*-LA-*grad*-LLA) samples still maintained high crystallinity. In contrast, a random PLA copolymer having 10% *meso*-LA content displayed no crystallization transitions on its second heating scan (Fig. S60). These results provided supporting evidence for stereo- and sequence-control copolymerization and highlighted the importance of this stereogradient control fashion.

### Chemical recycling of PLLA

**Figure 4.**
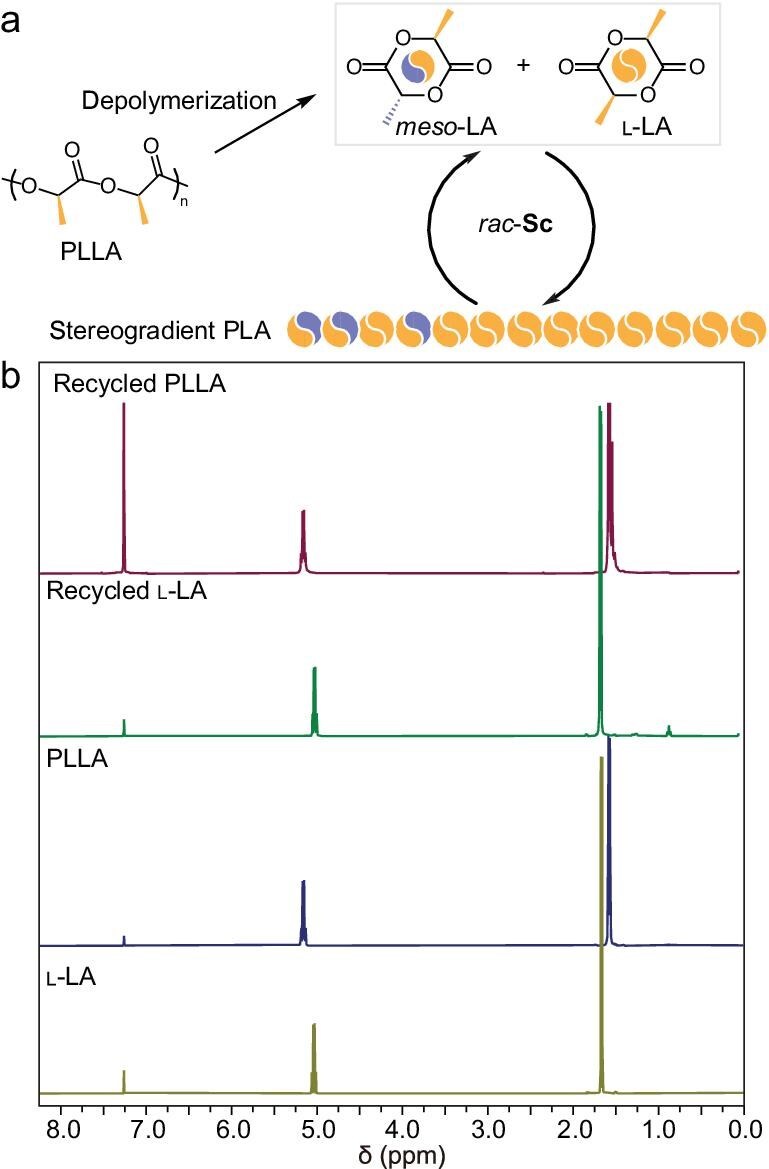
Chemical recyclability study of PLLA. (a) Chemical recycling of PLLA. (b) ^1^H NMR (CDCl_3_, 25°C) spectra of thermal depolymerization and repolymerization for PLLA.

With the establishment of well syndioselective-controlled and sequence-controlled copolymerization of *meso*-LA and l-LA by our Sc system, we set out to test our hypothesis for chemical recycling of PLLA. *rac*-Sc2 with a catalyst loading of 0.1–0.2 mol% promoted the depolymerization of PLLA at 180°C–200°C, giving a 99% yield of LA monomers containing 1%–2% *meso*-LA (Table S3, Entries 2–5). In the absence of the catalyst, only a trace amount of LA monomer was observed (Table S3, Entry 1), indicative of the significance of *rac*-Sc2. In comparison with the reported literature that Sn(Oct)_2_ gave a 92% yield of l-LA with 99% purity, and MgCl_2_ at 180°C for 18 h was found to give a 96% yield with 95% purity of l-LA [21,23], our *rac*-Sc2 demonstrated complete degradation at 200°C for 10.5 h, approaching 99% yield of pure l-LA. We speculated that the spirosalen ligand could adjust the acidity of the catalytic system, minimizing the racemization of l-LA during the degradation process. In addition, it allowed a better contact effectiveness between the catalyst and PLLA, thus improving the degradation efficiency. It should be noted that the epimerization of l-LA to *meso*-LA was inevitable during the depolymerization in the most commonly reported systems [19,20]. The recovered LA monomers (*meso*-LA:l-LA = 1:99 or 2:98) were subjected to repolymerization under similar conditions to our copolymerization study (Fig. 4). Excitingly, the recycled PLA samples displayed *M*_n_ values of 20.7 and 15.9 kDa, respectively (Table S4, Entries 1–2). Regarding the reduced molecular weights of the recycled PLA product compared with the analogues obtained from the direct copolymerization, we speculated that the recycled LA contained a small amount of initiator from the degradation of the original PLLA. Furthermore, the longer reaction time might promote the side reaction of chain transfer, leading to reduced molecular weights. The DSC analysis revealed that the recycled P(*meso*-LA-*grad*-LLA) with 1%–2% *meso*-LA composition exhibited *T*_m_ values of 146°C–154°C, which demonstrated the feasibility for chemical recycling of PLLA with tolerance of epimerization by our Sc catalysts (Fig. S74). It is worth noting that the presence of a trace amount of impurities in the recovered LA might slow down the repolymerization and lead to racemization. Consequently, the resulting recycled PLA might have a higher percentage of *meso*-LA composition, leading to lower *T*_m_ values.

## CONCLUSIONS

In summary, spirosalen–scandium complexes (Sc) promoted robust and stereocontrolled polymerization for both *rac*-LA and *meso*-LA and afforded perfectly heterotactic PLA with *P*_r_ values up to 0.99 and syndiotactic PLA with *P*_r_ values up to 0.98. The high polymerization reactivity and stereoselectivity of *meso*-LA has been exploited as an effective strategy to establish an epimerization-tolerant chemical recycling of PLLA, constructing the stereogradient block PLA P(*meso*-LA-*grad*-LLA) without compromising the material properties of the original PLLA. These findings manifested the beneficial effect of our Sc catalysts on stereocontrolled polymerization of LA and chemical recycling of PLLA.

## Supplementary Material

nwaf416_Supplemental_File
