# Supplementary material for: Spirosalen–scandium catalysts enable the epimerization-tolerant closed-loop circularity of poly(l-lactic acid)
Source: Natl Sci Rev. 2025 Sep 26;12(12):nwaf416. doi: 10.1093/nsr/nwaf416 (PMC12693492; doi:10.1093/nsr/nwaf416)
Supplement: nwaf416_Supplemental_File [file nwaf416_supplemental_file.pdf]

Spiro-Salen Scandium Catalysts Enable the Epimerization-Tolerant Closed-Loop Circularity of Poly(L-lactic acid)

Yu-Ting Huang[1], Hao-Yi Huang[1], Min Xie[1], Haifeng Xiang[2]*, Zhongzheng Cai[1]*, and Jian-Bo Zhu[1]*

1 National Engineering Laboratory of Eco-Friendly Polymeric Materials (Sichuan)
College of Chemistry, Sichuan University
29 Wangjiang Rd, Chengdu, 610064, P. R. China
2 College of Chemistry, Sichuan University
29 Wangjiang Rd, Chengdu, 610064, P. R. China

Supplementary Methods

TABLE OF CONTENTS

Materials and Methods 2
Chemicals 2
Reaction conditions 2
Instruments and characterizations 2
General Polymerization Procedures 4
Equations 4
General Polymerization Procedures 5
Kinetic studies for the ring-opening polymerization of LA 7
Polymer Characterizations 13
NMR spectra of syndiotactic PLA samples 13
NMR spectra of heterotactic PLA samples 20
SEC traces of PLA 25
TGA curves for PLA 29
DSC curves for PLA 33
Chemical Recycling to Monomer (CRM) 42
Supplementary References 54

Materials and Methods

Chemicals

Unless otherwise indicated, all starting materials were purchased from Adamas-beta, Energy Chemical and Innochem and used as received. L-Lactide (L-LA), *rac*-lactide (*rac*-LA) were purchased from Adamas-beta and Energy Chemical, *meso*-lactide (*meso*-LA) was obtained as a research gift from NatureWorks Co. All monomers were purified via sublimation twice and recrystallization from THF at −20 °C prior to polymerization. PLLA was obtained according to literature route.[1] Catalysts Sc(Spiro-salen$^{t\text{-Bu}, R}$)$[N(SiHMe_2)_2]_3$(THF) prepared according to literature procedures.[2]

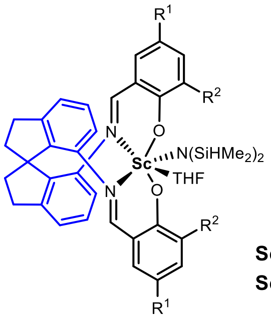

Sc1: R^1 = ^{t}Bu, R^2 = ^{t}Bu
Sc2: R^1 = ^{t}Bu, R^2 = CMePh$_2$

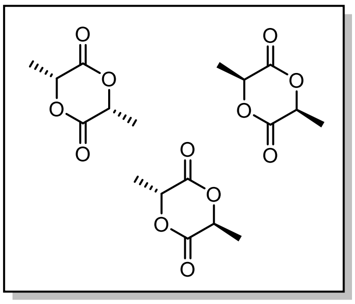

Reaction conditions

All synthesis and manipulations of air- and moisture-sensitive chemicals and materials were carried out in flamed Schlenk-type glassware on a dual-manifold Schlenk line, on a high-vacuum line, or in an inert gas (Ar)-filled glovebox. High-performance liquid chromatography (HPLC)-grade organic solvents were dried by Vigor YJC-5 and then stored over activated 4 Å molecular sieves.

Instruments and characterizations

^{1}H NMR Spectroscopy

^{1}H NMR spectra was recorded on an Agilent 400-MR DD2 or a Brüker Advance 400 spectrometer (^{1}H: 400 MHz). Chemical shifts (δ) for ^{1}H spectra is given in ppm relative to TMS. The residual solvent signals were used as references for ^{1}H spectra and the chemical shifts converted to the TMS scale ($CDCl_3$: δH = 7.26 ppm). The solvent employed and the respective measuring frequency are indicated for each experiment. All spectra were processed with MestReNova 14.1.1 software, and coupling

Size Exclusion Chromatography (SEC)

Measurements of polymer number-average molecular weight (M_n), and molecular weight distributions or dispersity indices ($Đ = M_w/M_n$) were performed via size exclusion chromatography (SEC). The SEC instrument consisted of an Agilent LC system equipped with one guard column and two PL gel 5 μm mixed-C gel permeation columns and coupled with an Agilent G7162A 1260 Infinity II RI detector; The analysis was performed at 40 °C using THF as the eluent at a flow rate of 1.0 mL/min. The instrument was calibrated with nine polystyrene standards, and chromatograms were processed with Agilent OpenLab CDS Acquisition 2.5 molecular weight characterization software.

Differential scanning calorimetry (DSC)

Differential scanning calorimetry (DSC) Melting-transition temperature (T_m) and glass-transition temperature (T_g) of purified and thoroughly dried polymer samples were measured by differential scanning calorimetry (DSC) on a TRIOS DSC25, TA Instrument. All T_g values were obtained from a second scan after the thermal history was removed from the first scan. All T_m and T_g values were obtained from a second scan after the thermal history was removed from the first heating scan. Unless otherwise indicated, the heating rate was 10 °C/min and cooling rate was 10 °C/min. The raw data was processed in the TA TRIOS software.

Thermo-Gravimetric Analysis (TGA)

Decomposition onset temperatures (T_{onset}) and maximum rate decomposition temperatures (T_{max}) of the polymers were measured by thermal gravimetric analysis (TGA) on a TGA55 Analyzer, TA Instrument. Polymer samples were heated from ambient temperature to 500 °C at a heating rate of 10 °C/min. Values of T_{max} were obtained from derivative (wt%/°C) vs. temperature (°C) plots and defined by the peak values, while T_d values were obtained from wt% vs. temperature (°C) plots and defined by the temperature of 5% weight loss. The raw data was processed in the TA TRIOS software.

High Performance Liquid Chromatography (HPLC)

HPLC was performed on Agilent 1260 Infinity II Quaternary LC system. Enantiomeric excesses (ee) were determined by chiral HPLC analysis on Daicel chiralcel IA columns. The column employed and the respective solvent mixture are indicated for each experiment. The chromatograms were processed with Agilent OpenLab CDS software.

General Polymerization Procedures

Inside glovebox, the polymerization was performed in 4 ml glass vials. To a solution of monomer and initiator *p*-tolylmethanol, the salen complex in solvent solution was added rapidly and stirred for a desired time. The polymerization was quenched by addition of 1 mL of benzoic acid/wet chloroform (10 mg mL^{-1}) and a 0.05 mL of aliquot was taken from the reaction mixture and prepared for ^{1}H NMR analysis to calculate the monomer conversion. The quenched mixture was precipitated into 20 mL of cold methanol, centrifuged (4500 rpm, 10 minutes, 3 times), washed with cold methanol to remove any unreacted monomer, and dried in a vacuum oven at 60−70 °C to a constant weight.

Equations

Stereoregularity of syndiotactic PLA

For polymerization of *meso*-LA, the degree of PLA syndiotacticity was determined by HD ^{1}H NMR of the methine region according to the relative intensities of the rrr tetrad (δ 5.15 ppm) overlapping with the rrm/mrr and mrm tetrads compared to the rmr tetrad (δ 5.22 ppm). [3]

$[rmr] = (P_m^2 + P_rP_m)/2$, $[rrr] = P_r^2 + P_rP_m/2$, $[mrr] = [rrm] = P_rP_m/2$, $[mrm] = P_m^2/2$.

$P_r = 1 - P_m = 1–2[rmr]$.

Stereoregularity of heterotactic PLA

For polymerization of *rac*-LA, the degree of PLA heterotacticity was determined by HD ^{1}H NMR of the methine region according to the relative intensities of the mrm (δ 5.16 ppm) and rmr (δ 5.23 ppm) tetrads relative to the other tetrads: mmm (δ 5.17 ppm), rmm (δ 5.22 ppm), mmr (δ 5.18 ppm). [3]

$[mmm] = P_m^2 + P_rP_m/2$, $[mmr] = [rmm] = P_rP_m/2$, $[rmr] = P_r^2/2$, $[mrm] = (P_r^2 + P_rP_m)/2$.

$P_r = 1 - P_m = \sqrt{2[rmr]}$.

General Polymerization Procedures

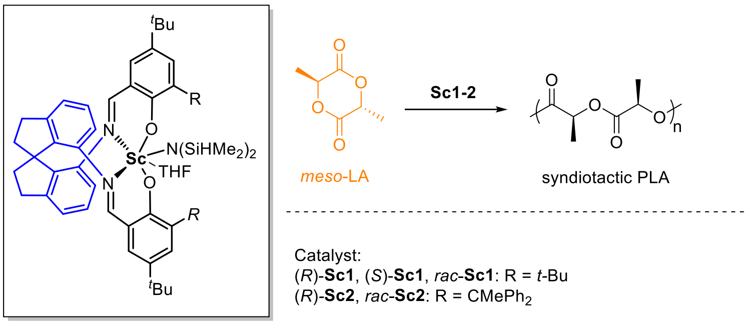

Table S1. ROP of *meso*-LA with Spiro-salen$^{t\text{-Bu, R}}$ complex **Sc1-2**.[a]

Entry	Catalyst	Sol.	[M]/[Sc]/[I]	Time (min)	Conv.[b] (%)	TOF[c] (h^{-1})	$M_{n,calcd}$[d] (kDa)	M_n [e] (kDa)	$Đ$[e]	P_r[f]
**1**	(*R*)-**Sc1**	THF	200:1:1	90	98	131	28.3	26.1	1.05	0.96
**2**	(*R*)-**Sc1**	TOL	200:1:1	7	>99	1697	28.6	36.8	1.07	0.95
**3**	(*R*)-**Sc1**	DCM	200:1:1	20	>98	588	28.3	50.6	1.12	0.95
**4**	(*R*)-**Sc1**	THF	500:1:1	6 h	>99	82	71.4	54.2	1.06	0.96
**5**	(*R*)-**Sc1**	THF	1000:1:1	24 h	96	40	138	101	1.05	0.95
**6**	(*S*)-**Sc1**	THF	200:1:1	1.2 h	98	163	28.3	25.2	1.06	0.95
**7**	*rac*-**Sc1**	THF	200:1:1	90	96	128	27.8	33.0	1.06	0.95
**8**	*rac*-**Sc1**	TOL	200:1:1	12	99	990	28.6	34.4	1.09	0.93
**9**	(*R*)-**Sc2**	THF	200:1:1	11.5 h	81	14	23.4	18.5	1.07	0.92
**10**	*rac*-**Sc2**	THF	50:1:1	2.5 h	99	20	7.32	8.30	1.06	0.98
**11**	*rac*-**Sc2**	THF	200:1:1	11 h	93	17	26.9	25.6	1.04	0.98
**12**	*rac*-**Sc2**	TOL	200:1:1	6 h	99	33	28.6	29.6	1.05	0.94
**13**	*rac*-**Sc2**	THF	500:1:1	24 h	76	16	54.8	42.7	1.04	0.98
**14**	*rac*-**Sc2**	THF	1000:1:1	84 h	28	3	40.4	47.5	1.03	N.D.
**15**[g]	(*R*)-**Sc1**	Neat	200:1:1	5	75	1800	21.7	21.8	1.09	0.94
**16**[g]	(*R*)-**Sc1**	Neat	2000:1:1	8	49	7350	141	71.2	1.06	0.96
**17**[g]	*rac*-**Sc2**	Neat	1000:1:1	30	54	1080	77.9	28.3	1.04	0.90
**18**[h]	Precursor	THF	200:1:1	170	>99	70	28.6	83.6	1.38	0.48

[a] Reaction conditions: *meso*-LA = 150 mg (1.04 mmol), [*meso*-LA] = 1.0 M, *p*-tolylmethanol as the initiator, room temperature. [b] Monomer conversion measured by ^{1}H NMR of the quenched solution. [c] Turnover frequency TOF = {[*meso*-LA]/[**Sc**] × Conv. (%)}/time(h). [d] M_n(calcd) = MW(*meso*-LA) × [*meso*-LA]/[*p*-tolylmethanol] × Conv. (%) + MW of chain-end groups (*p*-tolylmethanol). [e] Number-average molecular weight (M_n) and dispersity index ($Đ = M_w/M_n$), determined by size Exclusion Chromatography (SEC) at 40 °C in THF. [f] P_r is theprobability of syndio-enchainment determined by Homonuclear decoupled ^{1}H NMR spectroscopy. [g] Reaction temperature: 55 °C, neat. [h] Catalyst: Precursor = $Sc(N(SiHMe_2)_2)_3(THF)$.

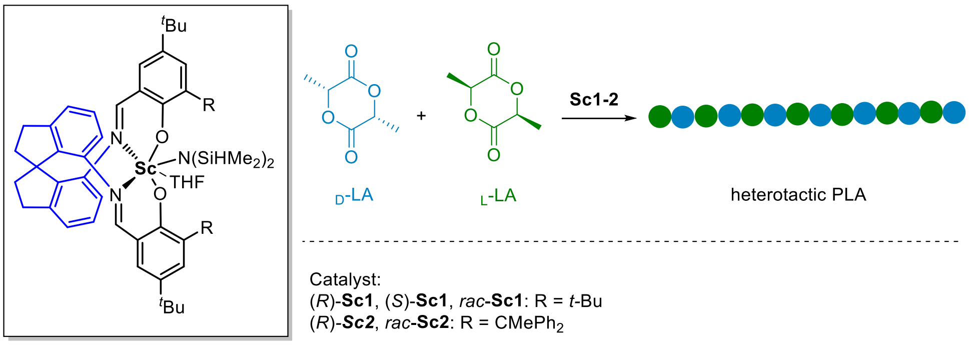

Table S2. ROP of *rac*-LA with Spiro-salen$^{t\text{-Bu, R}}$ complex **Sc1-2**.[a]

Entry	Catalyst	Sol.	[M]/[Sc]/[I]	Time (min)	Conv.[b] (%)	TOF[c] (h^{-1})	$M_{n,calcd}$[d] (kDa)	M_n[e] (kDa)	$Đ$[e]	P_r[f]
**1**	(*R*)-**Sc1**	THF	200:1:1	90	88	117	101	25.2	1.07	0.97
**2**	(*R*)-**Sc1**	TOL	200:1:1	20	93	558	83	32.8	1.06	0.83
**3**	(*R*)-**Sc1**	DCM	200:1:1	70	98	168	93	29.1	1.06	0.91
**4**	(*R*)-**Sc1**	THF	500:1:1	10 h	88	44	114	55.7	1.06	0.97
**5**	(*S*)-**Sc1**	THF	200:1:1	70	86	147	91	27.5	1.08	0.97
**6**	*rac*-**Sc1**	THF	200:1:1	1.2 h	93	155	94	28.7	1.04	0.98
**7**	(*R*)-**Sc2**	THF	200:1:1	11.5 h	64	11	96	19.3	1.05	0.83
**8**	*rac*-**Sc2**	THF	200:1:1	19.5 h	91	9	104	25.2	1.05	0.99
**9**	*rac*-**Sc2**	THF	500:1:1	24 h	44	9	118	27.0	1.05	0.99
**10**[g]	(*R*)-**Sc1**	Neat	200:1:1	20	91	546	123	21.3	1.30	0.85
**11**[g]	*rac*-**Sc2**	Neat	1000:1:1	40	83	1245	131	91.3	1.31	0.85
**12**[g]	*rac*-**Sc2**	Neat	2000:1:1	3.5 h	61	348	519	33.9	1.20	0.79
**13**[h]	Precursor	THF	200:1:1	170	85	60	54	45.3	1.92	0.62

[a] Reaction conditions: *rac*-LA = 150 mg (1.04 mmol), [*rac*-LA] = 1.0 M, *p*-tolylmethanol as the initiator, THF as the solvent, room temperature. [b] Monomer conversion measured by ^{1}H NMR of the quenched solution. [c] Turnover frequency TOF = {[*rac*-LA]/[**Sc**] × Conv. (%)}/time(h). [d] M_n(calcd) = MW(*rac*-LA) × [*rac*-LA]/[*p*-tolylmethanol] × Conv. (%) + MW of chain-end groups (*p*-tolylmethanol). [e] Number-average molecular weight (M_n) and dispersity index ($Đ = M_w/M_n$), determined by size Exclusion Chromatography (SEC) at 40 °C in THF. [f] P_r is the probability of hetero-enchainment determined by Homonuclear decoupled ^{1}H NMR spectroscopy. [g] Reaction temperature: 130 °C, neat. [h] Catalyst: Precursor = $Sc(N(SiHMe)_2)_2)_3(THF)$.

Kinetic studies for the ring-opening polymerization of LA

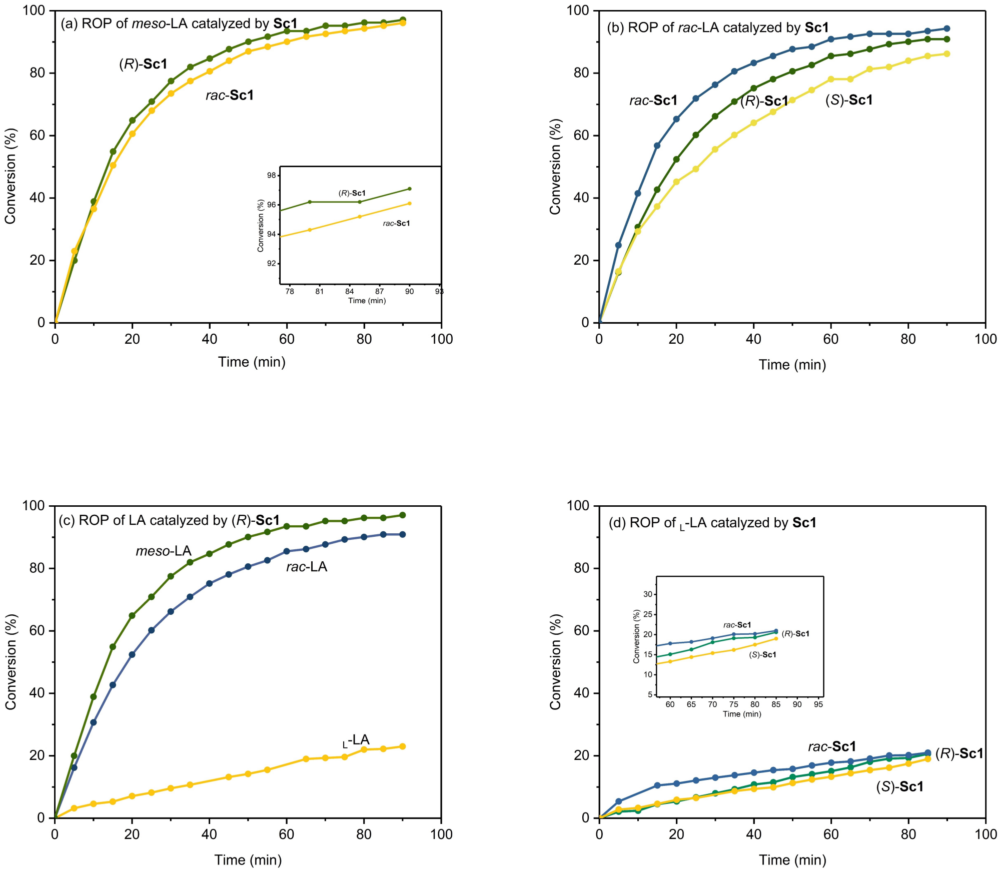

Fig. S1. Plots of monomer conversions over time during the ROP of LA catalyzed by **Sc1**. [LA]/[**Sc1**]/[I] = 200/1/1 in THF.

(a) ROP of *meso*-LA catalyzed by (*R*)-**Sc1** and *rac*-**Sc1**, respectively. (b) ROP of *rac*-LA catalyzed by (*R*)-**Sc1**, (*S*)-**Sc1** and *rac*-**Sc1**, respectively. (c) ROP of *meso*-LA, *rac*-LA and L-LA catalyzed by (*R*)-**Sc1**, respectively. (d) ROP of L-LA catalyzed by (*R*)-**Sc1**, (*S*)-**Sc1** and *rac*-**Sc1,** respectively.

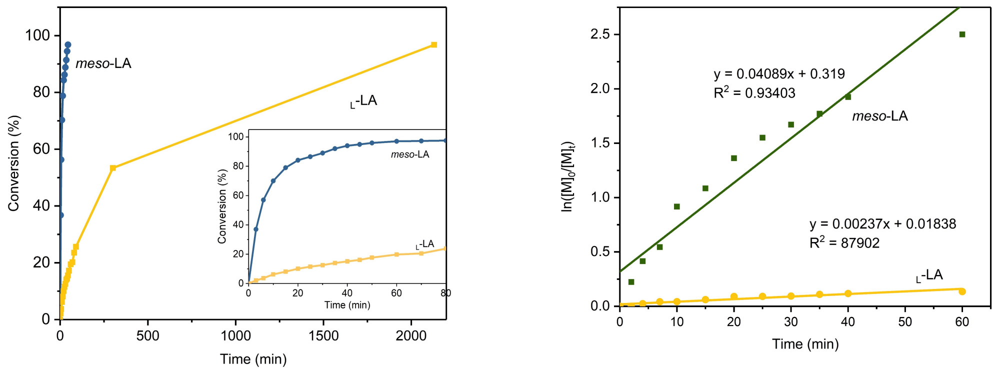

Fig. S2. Plots of monomer conversions over time during the ROP of *meso*-LA and L-LA consumption by (*S*)-**Sc1** catalyst in THF at room temperature, [L-LA]/[*meso*-LA]/[(*S*)-**Sc1**]/[I] = 190/10/1/1, [LA] = 1.0 M.

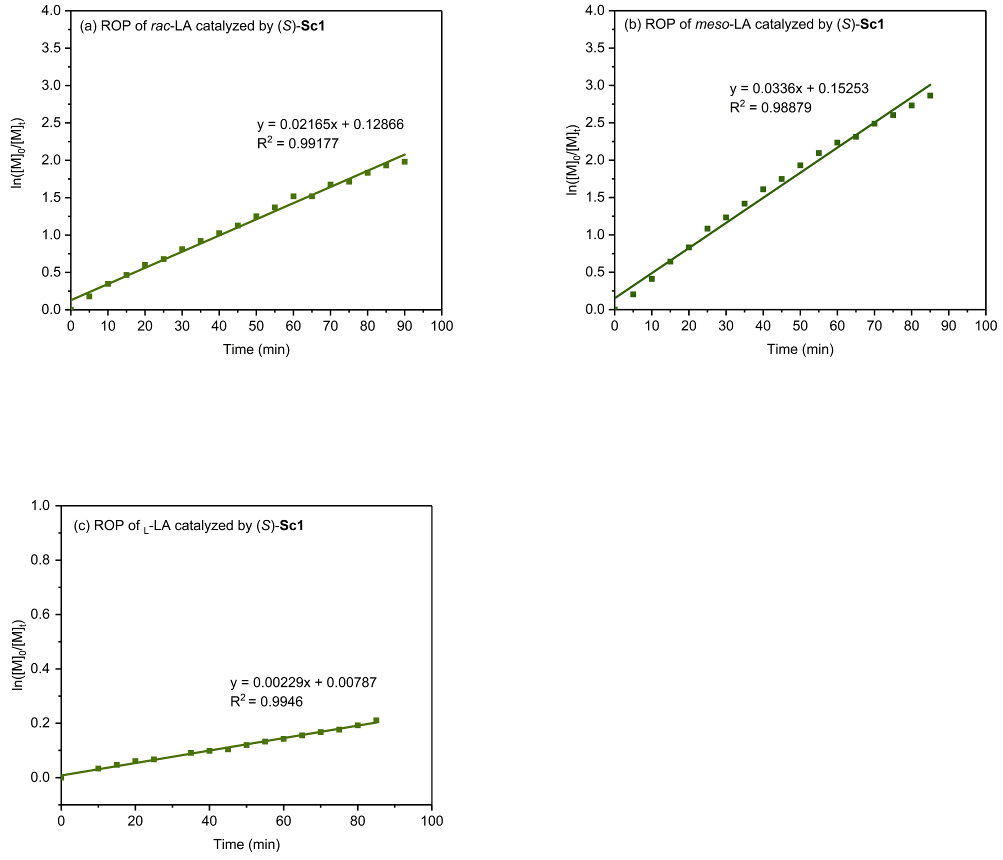

Fig. S3. Kinetics of LA consumption by (*S*)-**Sc1** catalyst in THF at room temperature. ([LA]/[(*S*)-**Sc1**]/[I] = 200/1/1, [LA] = 1.0 M).

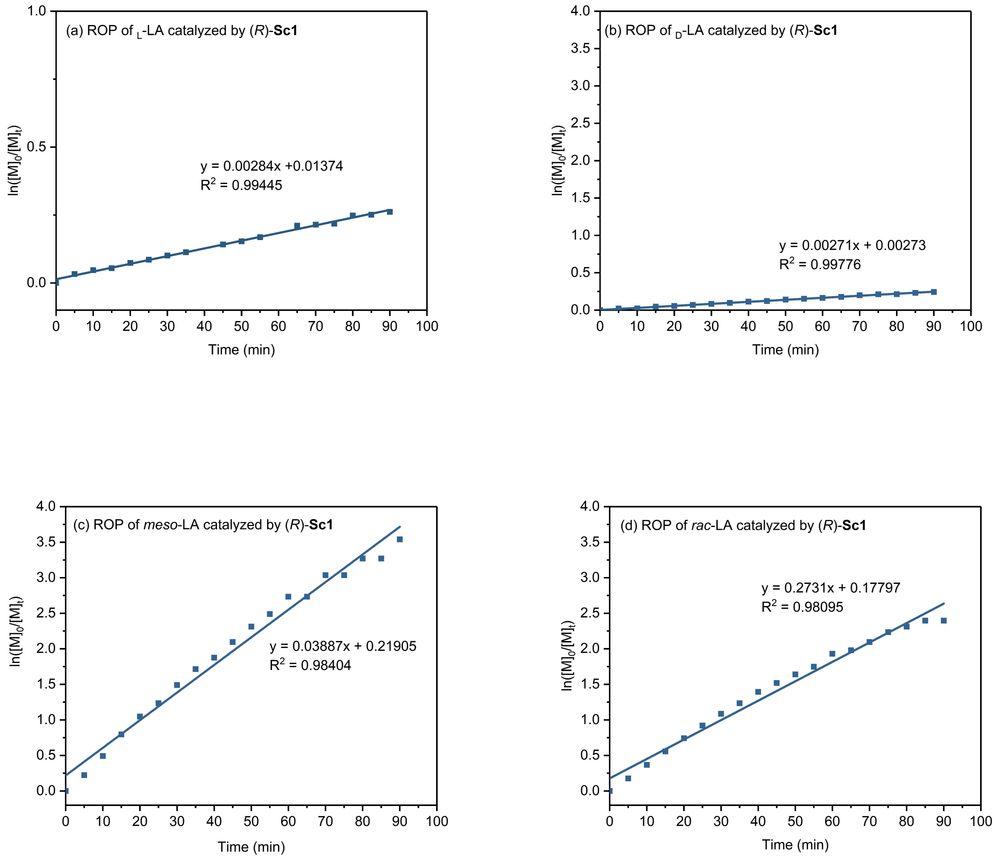

Fig. S4. Kinetics of LA consumption by (*R*)-**Sc1** catalyst in THF at room temperature. ([LA]/[(*R*)-**Sc1**]/[I] = 200/1/1, [LA] = 1.0 M).

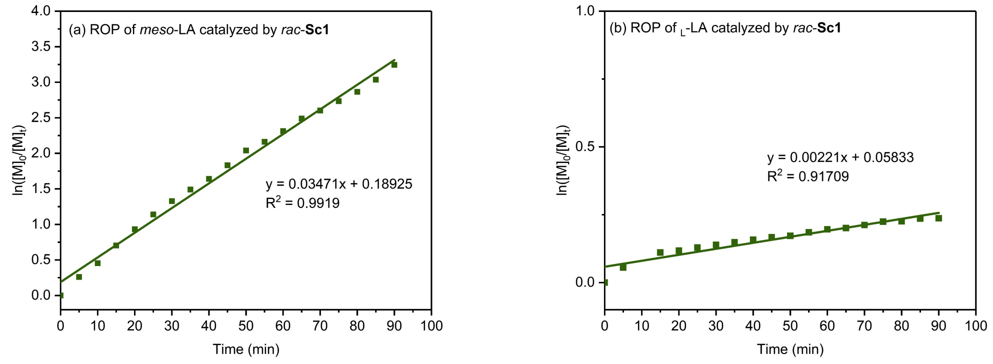

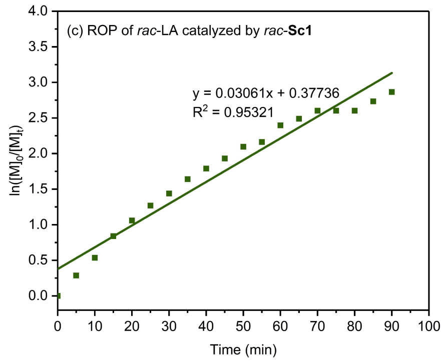

Fig. S5. Kinetics of LA consumption by *rac*-**Sc1** catalyst in THF at room temperature. ([LA]/[*rac*-**Sc1**]/[I] = 200/1/1, [LA] = 1.0 M).

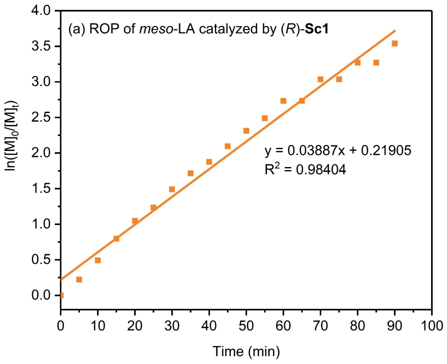

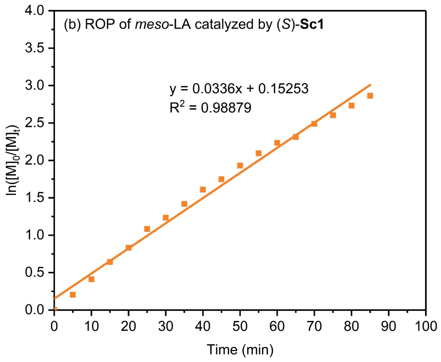

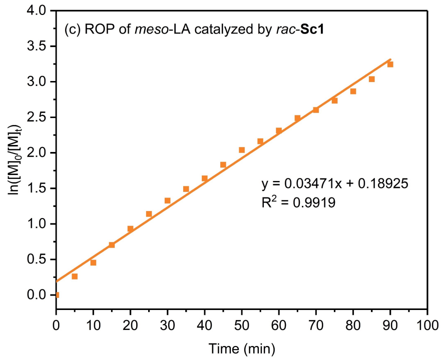

Fig. S6. Kinetics of *meso*-LA consumption by **Sc1** catalyst in THF at room temperature. ([*meso*-LA]/[**Sc1**]/[I] = 200/1/1, [*meso*-LA] = 1.0 M).

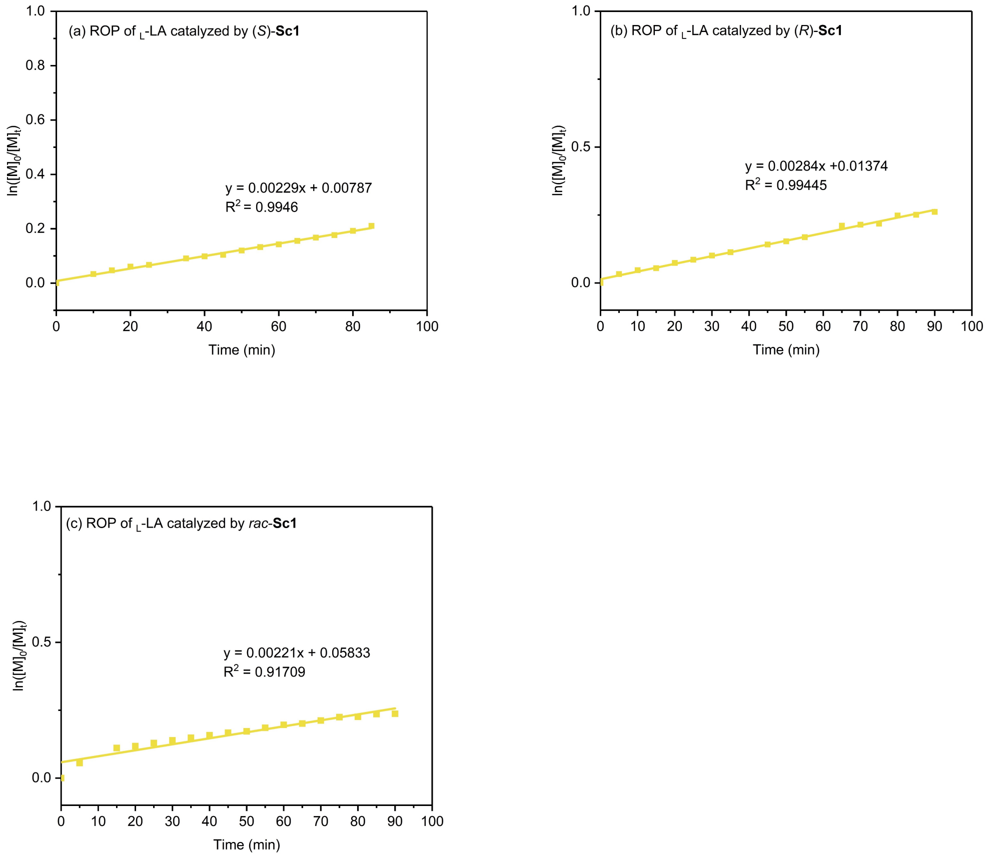

Fig. S7. Kinetics of L-LA consumption by **Sc1** catalyst in THF at room temperature. ([L-LA]/[**Sc1**]/[I] = 200/1/1, [L-LA] = 1.0 M).

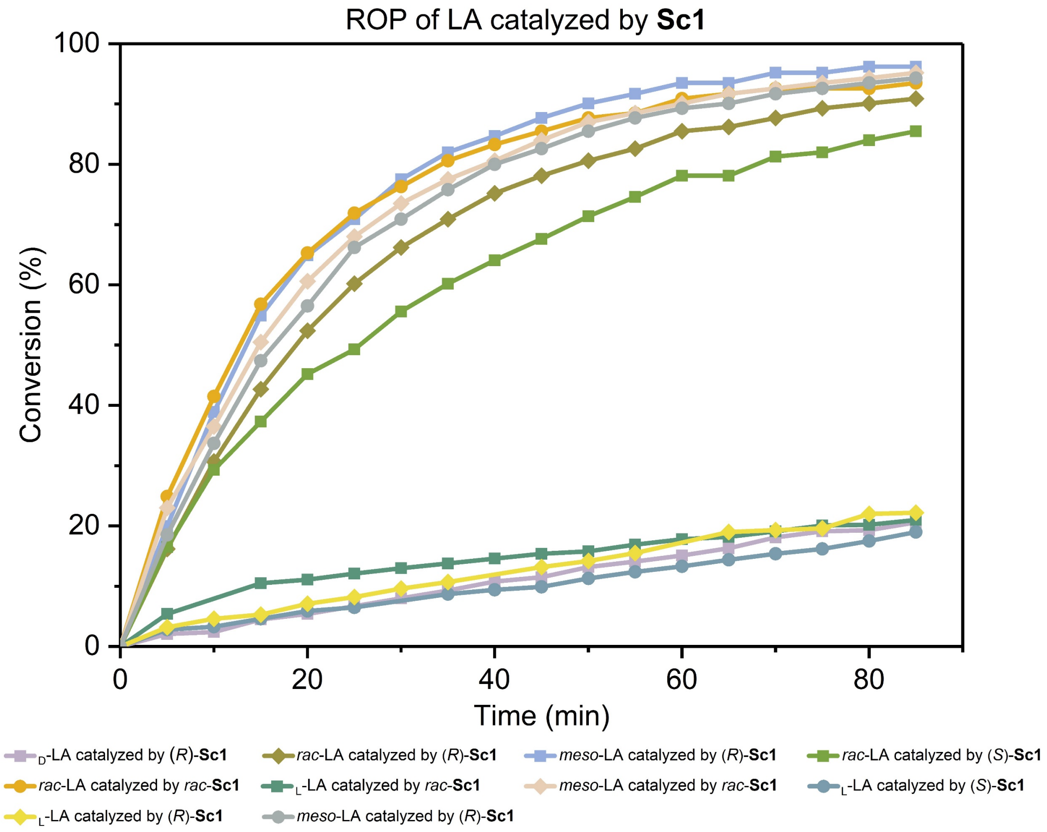

Fig. S8. A summary of plots of monomer conversion as a function of time for the ROP of LA catalyzed by **Sc1**.

Polymer Characterizations

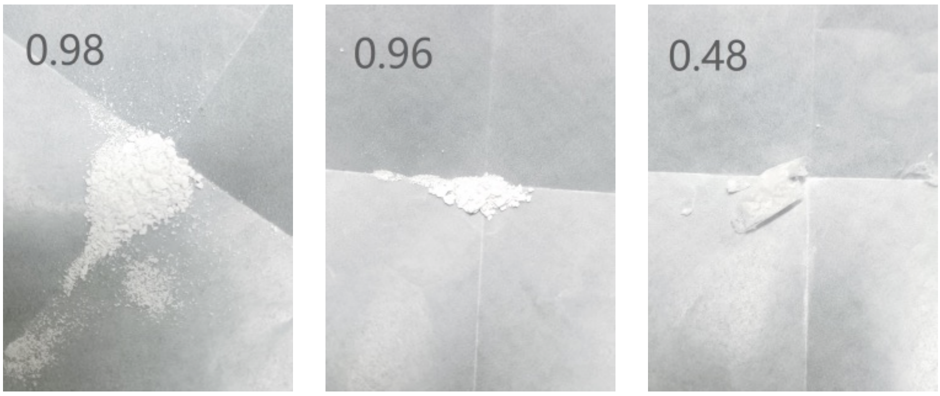

Fig. S9. Different syndiotactic PLA. PLA were produced by [*meso*-LA]/[**Sc**]/[I] = 200/1/1 in THF.

(1) Syndiotactic PLA with P_r = 0.98 obtained by *rac*-**Sc2**. (2) Syndiotactic PLA with P_r = 0.96 obtained by (*R*)-**Sc1**. (3) Random PLA with P_r = 0.48 obtained by $Sc(N(SiHMe_2)_2)_3(THF)$.

NMR spectra of syndiotactic PLA samples

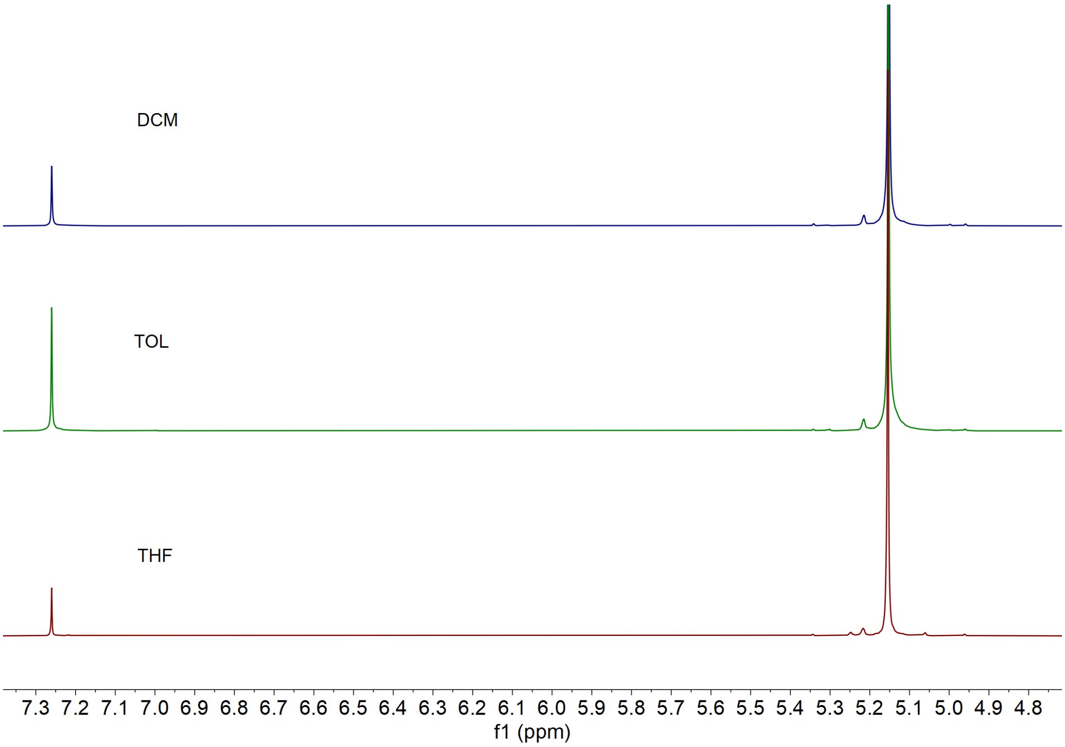

Fig. S10. Homonuclear decoupled ^{1}H NMR ($CDCl_3$, 25 °C) spectra of syndiotactic PLA samples. Syndiotactic PLA samples were produced by [*meso*-LA]/[(*R*)-**Sc1**]/[I] = 200/1/1 in different solvents.

(1) PLA (P_r = 0.95) was produced in DCM (bule line). (2) PLA (P_r = 0.95) was produced in TOL (green line). (3) PLA (P_r = 0.96) was produced in THF (red line).

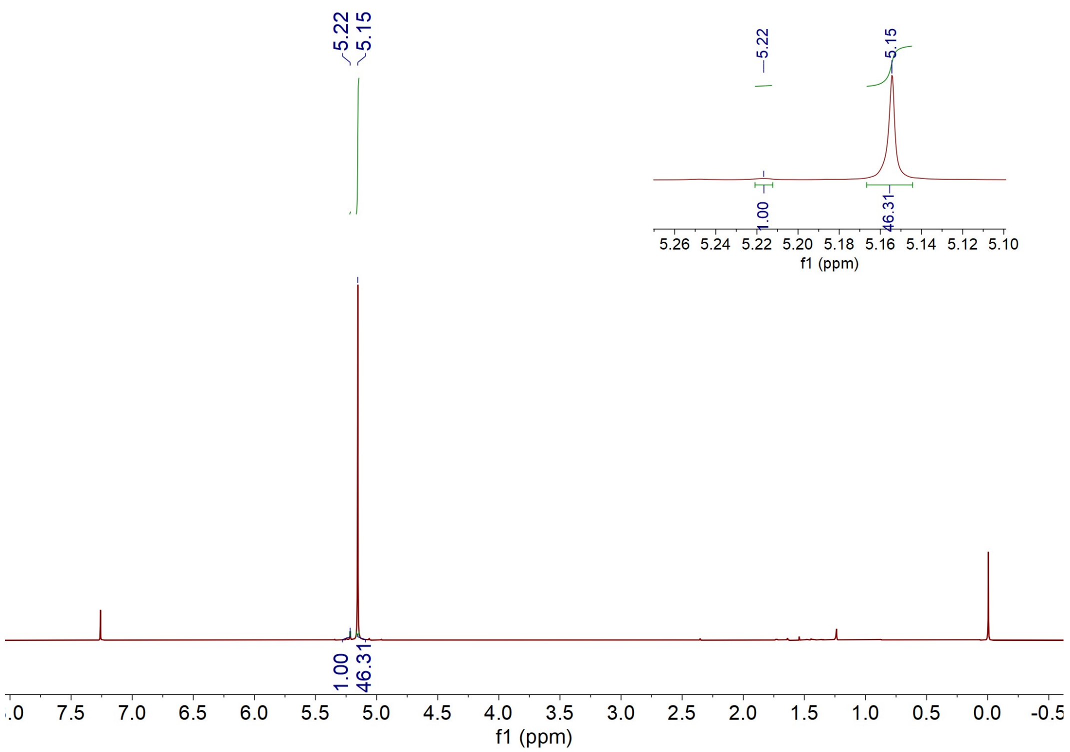

Fig. S11. Homonuclear decoupled ^{1}H NMR ($CDCl_3$, 25 °C) spectrum of syndiotactic PLA (P_r = 0.96). PLA was produced by [*meso*-LA]/[(*R*)-**Sc1**]/[I] = 200/1/1 in THF.

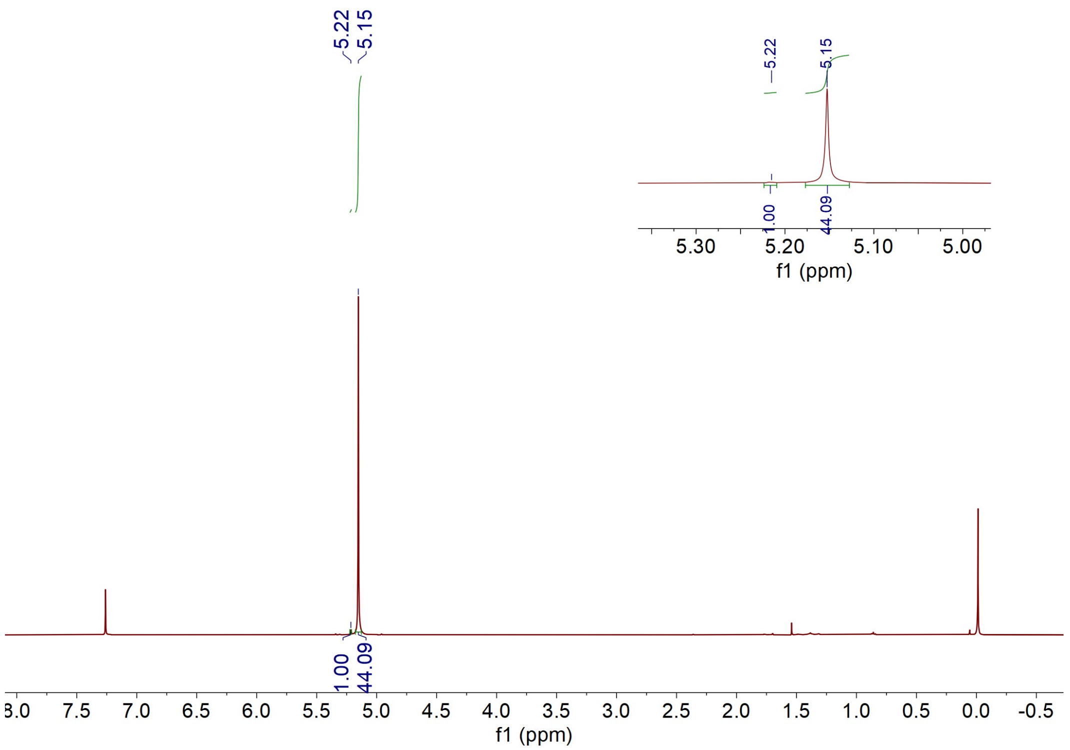

Fig. S12. Homonuclear decoupled ^{1}H NMR ($CDCl_3$, 25 °C) spectrum of syndiotactic PLA (P_r = 0.96). PLA was produced by [*meso*-LA]/[(*R*)-**Sc1**]/[I] = 500/1/1 in THF.

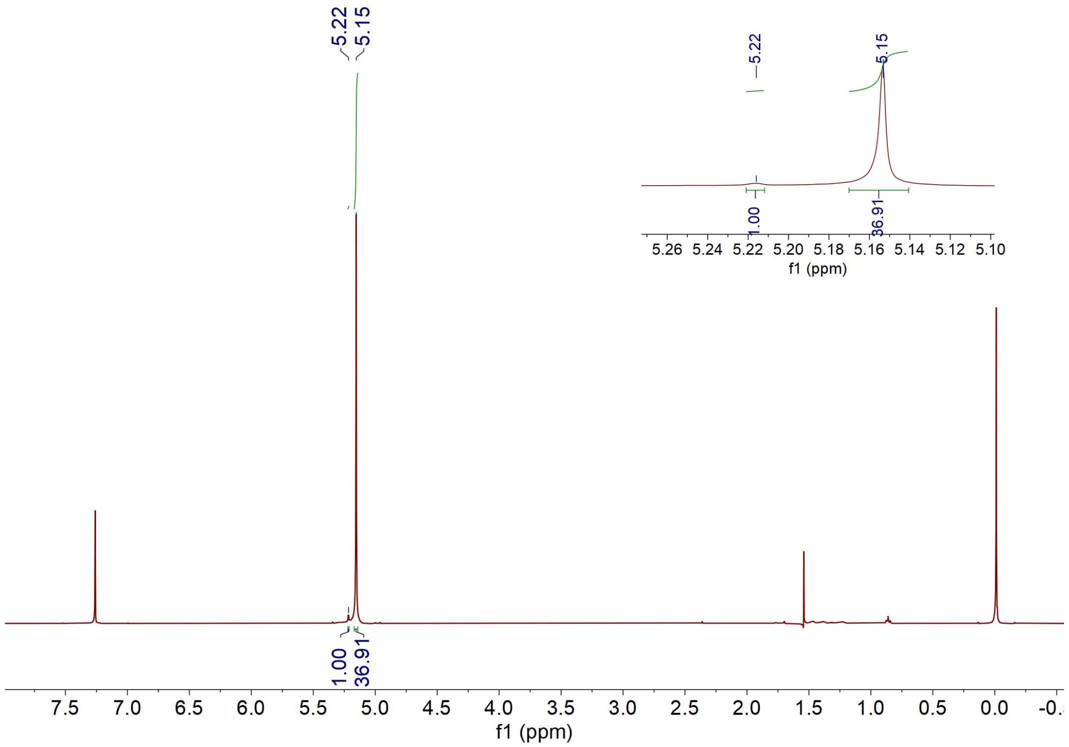

Fig. S13. Homonuclear decoupled ^{1}H NMR ($CDCl_3$, 25 °C) spectrum of syndiotactic PLA (P_r = 0.95). PLA was produced by [*meso*-LA]/[(*S*)-**Sc1**]/[I] = 200/1/1 in THF.

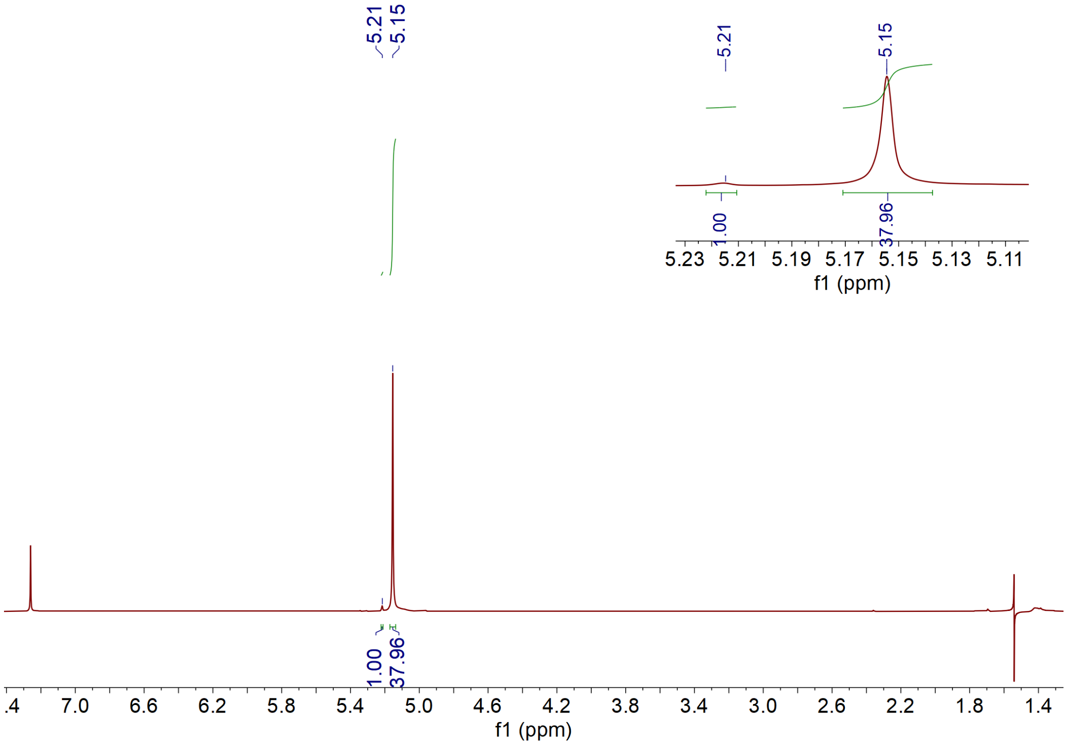

Fig. S14. Homonuclear decoupled ^{1}H NMR ($CDCl_3$, 25 °C) spectrum of syndiotactic PLA (P_r = 0.95). PLA was produced by [*meso*-LA]/[*rac*-**Sc1**]/[I] = 200/1/1 in THF.

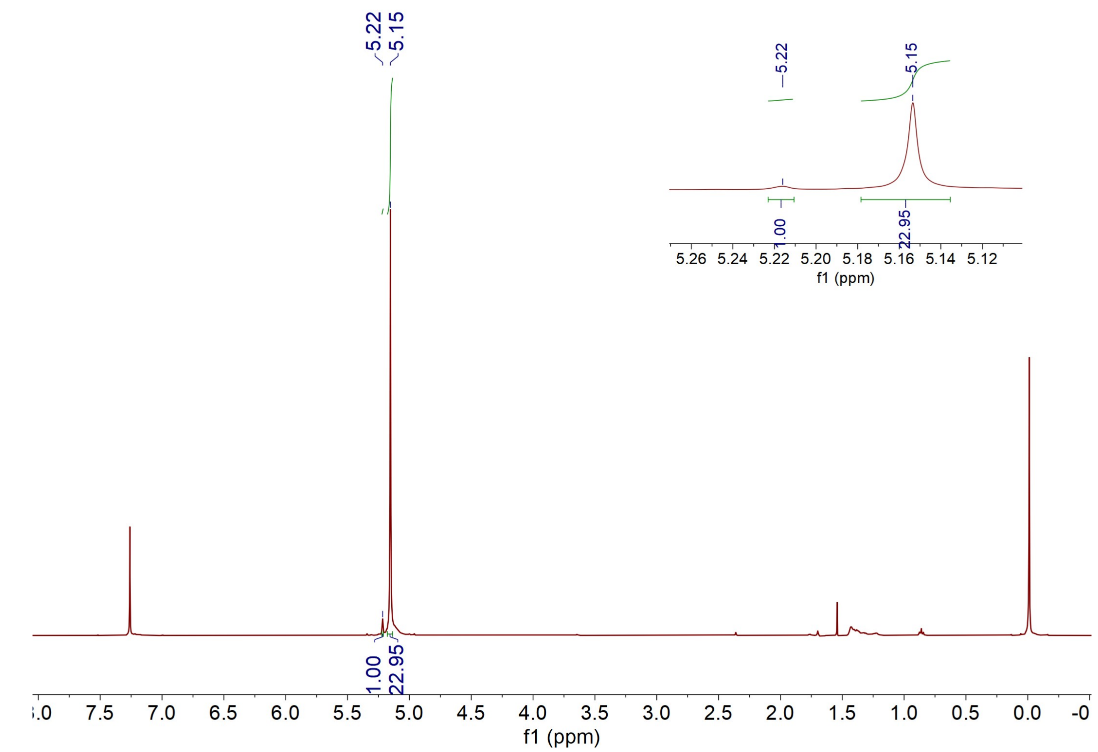

Fig. S15. Homonuclear decoupled ^{1}H NMR ($CDCl_3$, 25 °C) spectrum of syndiotactic PLA (P_r = 0.92). PLA was produced by [*meso*-LA]/[(*R*)-**Sc2**]/[I] = 200/1/1 in THF.

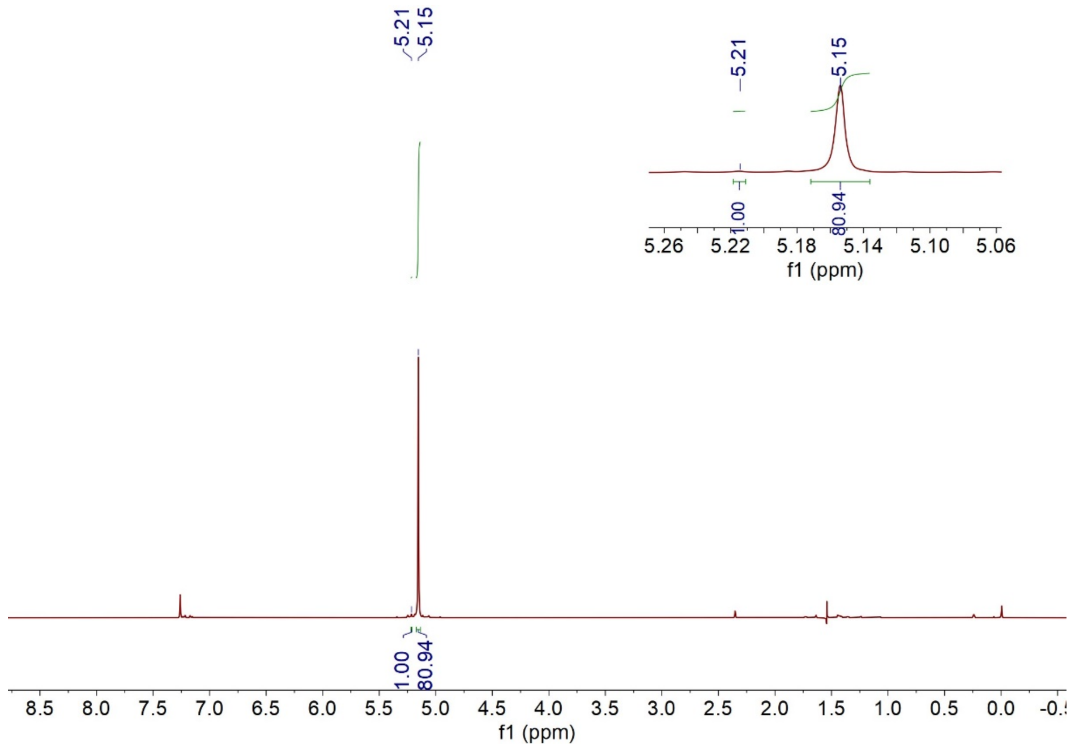

Fig. S16. Homonuclear decoupled ^{1}H NMR ($CDCl_3$, 25 °C) spectrum of syndiotactic PLA (P_r = 0.98). PLA was produced by [*meso*-LA]/[*rac*-**Sc2**]/[I] = 50/1/1 in THF.

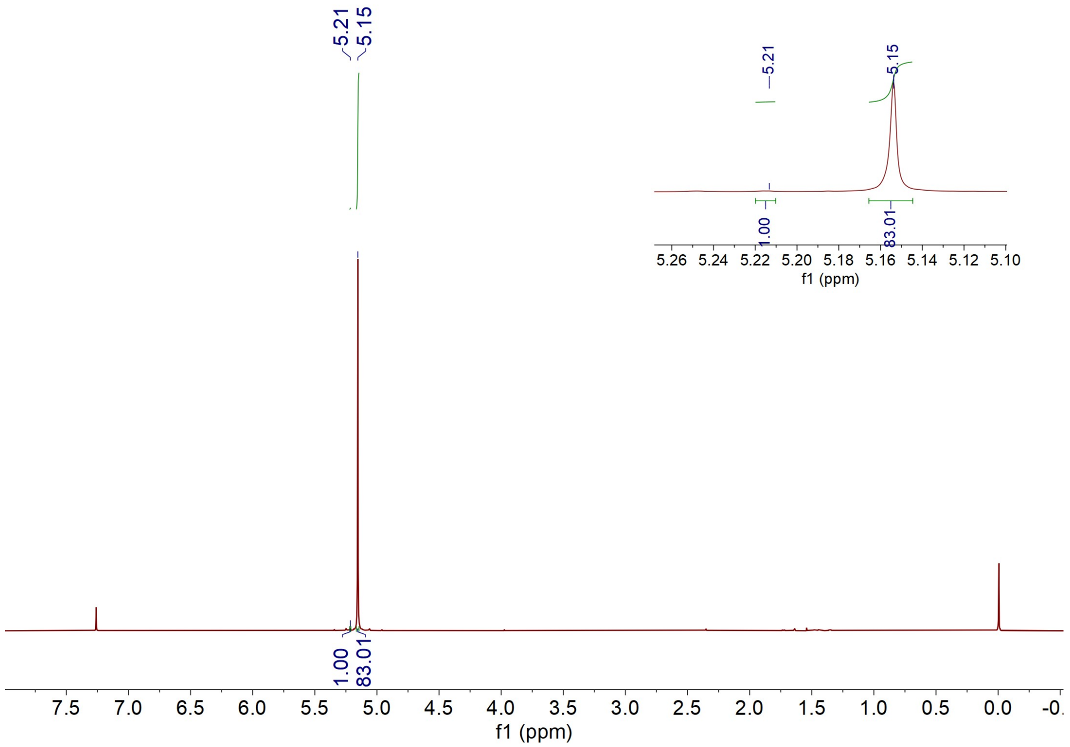

Fig. S17. Homonuclear decoupled ^{1}H NMR ($CDCl_3$, 25 °C) spectrum of syndiotactic PLA (P_r = 0.98). PLA was produced by [*meso*-LA]/[*rac*-**Sc2**]/[I] = 200/1/1 in THF.

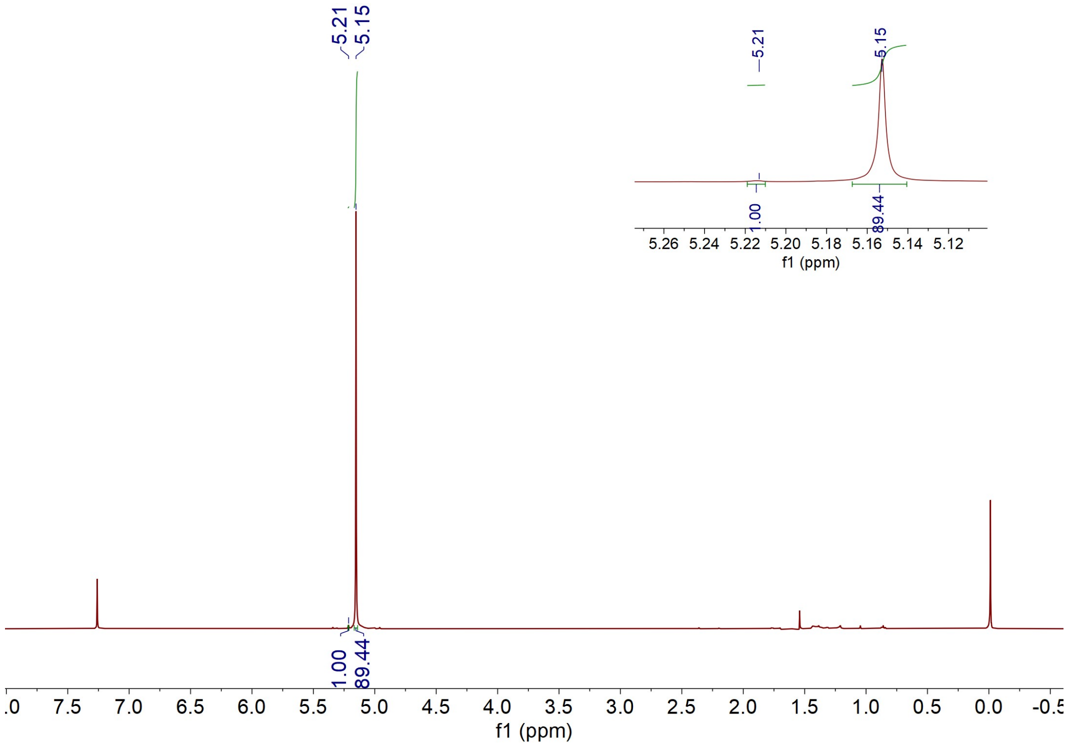

Fig. S18. Homonuclear decoupled ^{1}H NMR ($CDCl_3$, 25 °C) spectrum of syndiotactic PLA (P_r = 0.98). PLA was produced by [*meso*-LA]/[*rac*-**Sc2**]/[I] = 500/1/1 in THF.

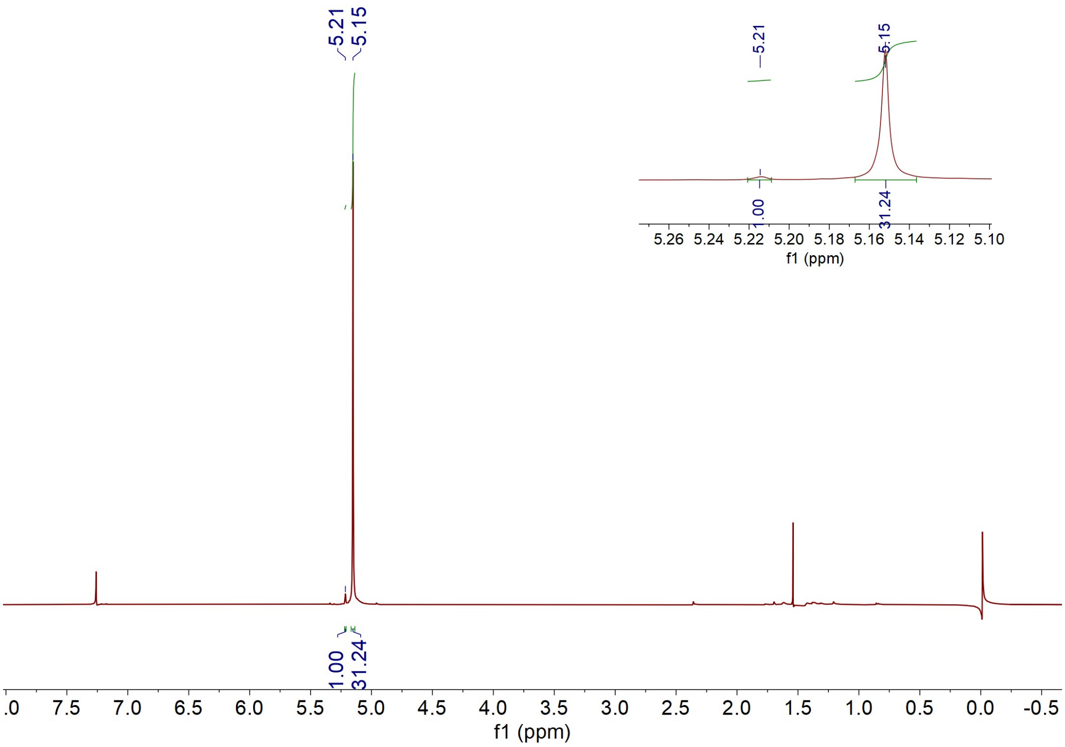

Fig. S19. Homonuclear decoupled ^{1}H NMR ($CDCl_3$, 25 °C) spectrum of syndiotactic PLA (P_r = 0.94). PLA was produced by [*meso*-LA]/[(*R*)-**Sc1**]/[I] = 200/1/1, neat.

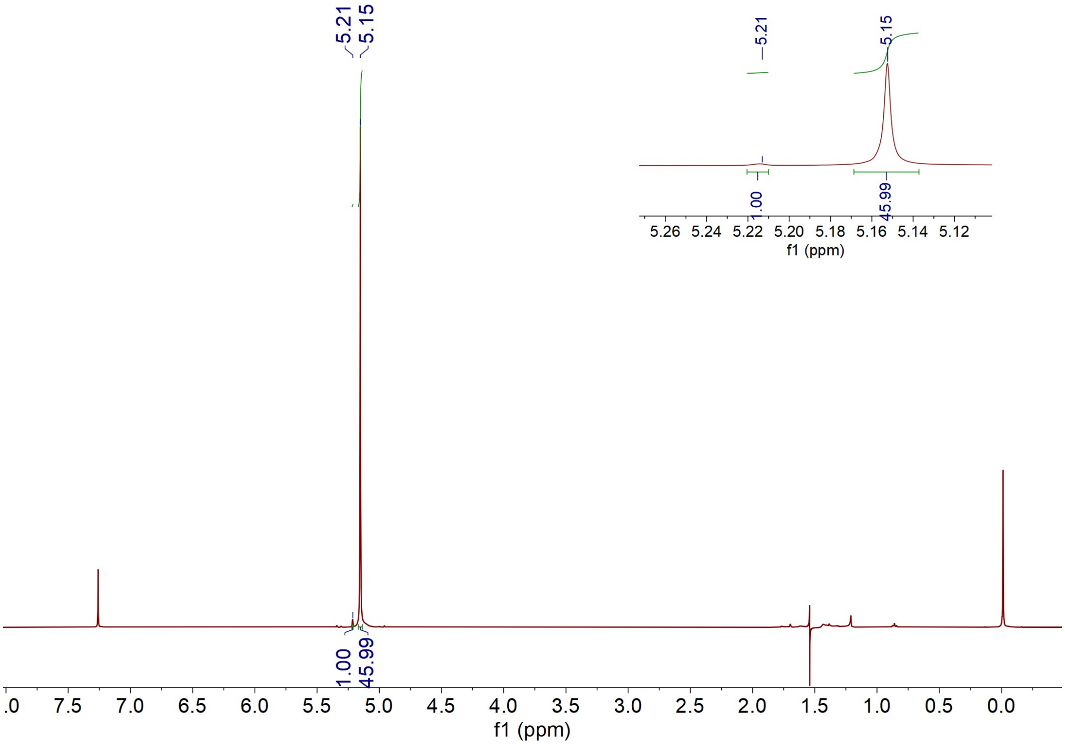

Fig. S20. Homonuclear decoupled ^{1}H NMR ($CDCl_3$, 25 °C) spectrum of syndiotactic PLA (P_r = 0.96). PLA was produced by [*meso*-LA]/[(*R*)-**Sc1**]/[I] = 2000/1/1, neat.

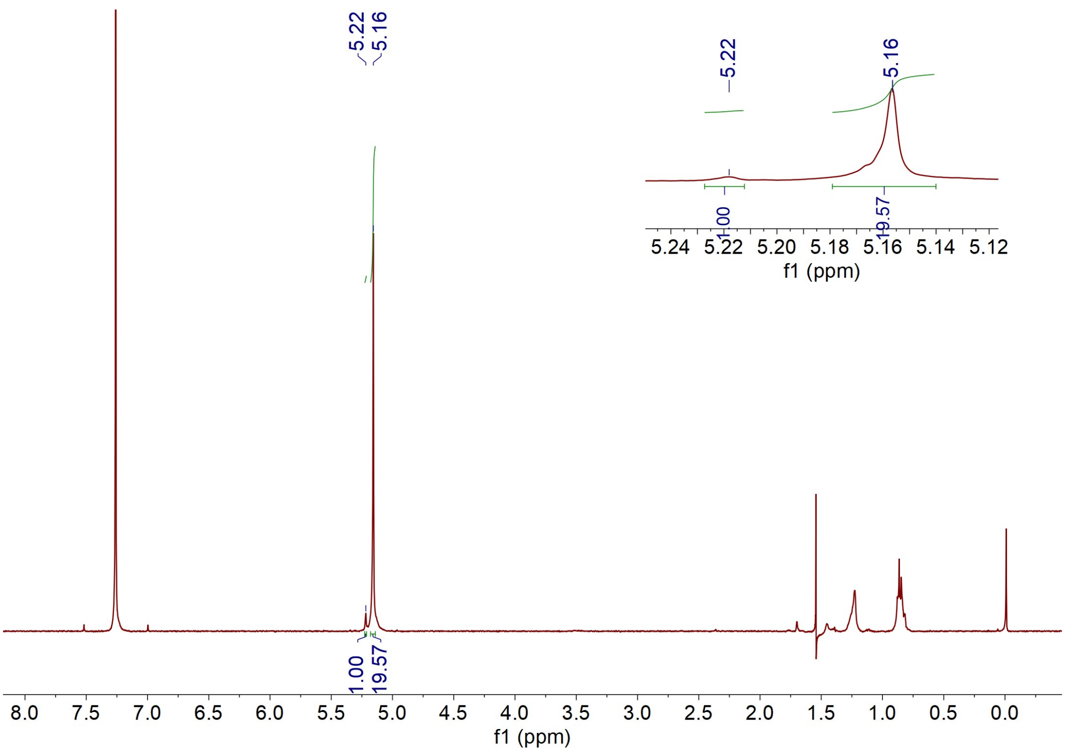

Fig. S21. Homonuclear decoupled ^{1}H NMR ($CDCl_3$, 25 °C) spectrum of syndiotactic PLA (P_r = 0.90). PLA was produced by [*meso*-LA]/[*rac*-**Sc2**]/[I] = 1000/1/1, neat.

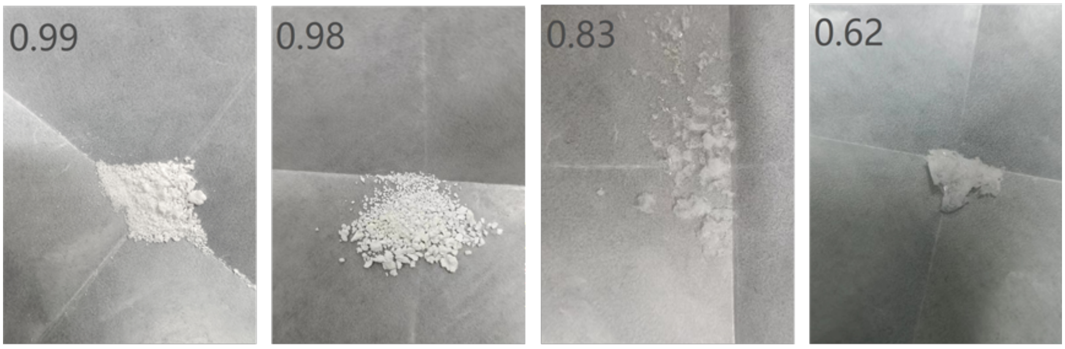

Fig. S22. Different heterotactic PLA. PLA were produced by [*rac*-LA]/[**Sc**]/[I] = 200/1/1.
(1) Heterotactic PLA with P_r = 0.99 obtained by *rac*-**Sc2** in THF. (2) Heterotactic PLA with P_r = 0.98 obtained by *rac*-**Sc1** in THF. (3) Heterotactic PLA with P_r = 0.83 obtained by (*R*)-**Sc1** in TOL. (4) Random PLA with P_r = 0.62 obtained by Sc(N(SiHMe$_2$)$_2$)$_3$(THF) in THF.

NMR spectra of heterotactic PLA samples

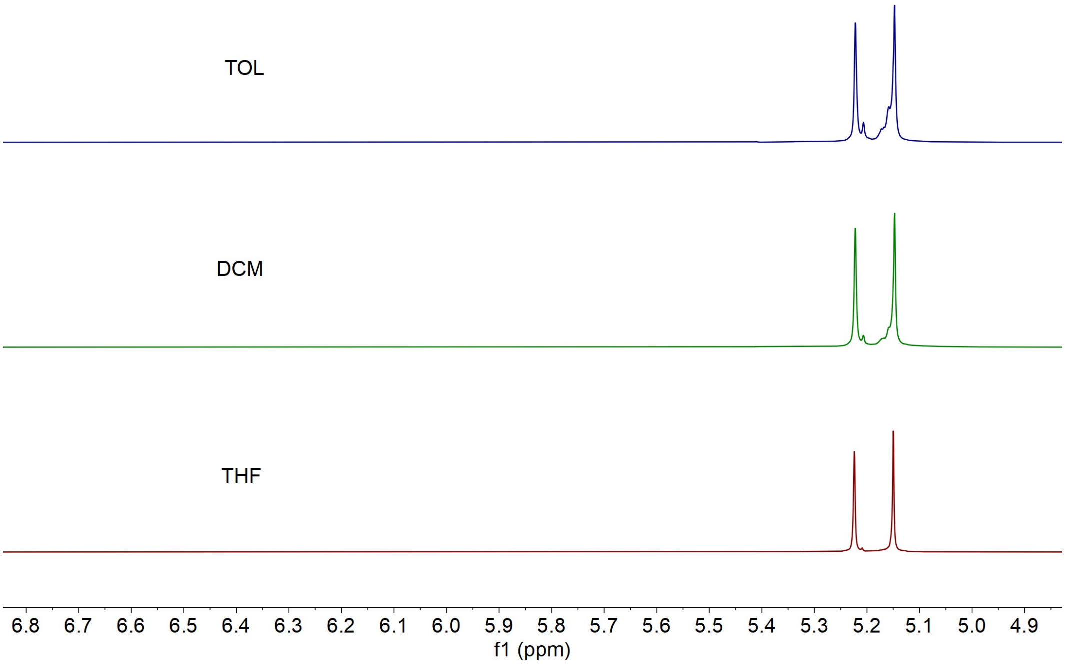

Fig. S23. Homonuclear decoupled ^{1}H NMR (CDCl$_3$, 25 °C) spectra of heterotactic PLA. Heterotactic PLA samples were produced by [*rac*-LA]/[(*R*)-**Sc1**]/[I] = 200/1/1 in different solvents.
(1) PLA (P_r = 0.83) was produced in TOL (blue line). (2) PLA (P_r = 0.91) was produced in DCM (green line). (3) PLA (P_r = 0.97) was produced in THF (red line).

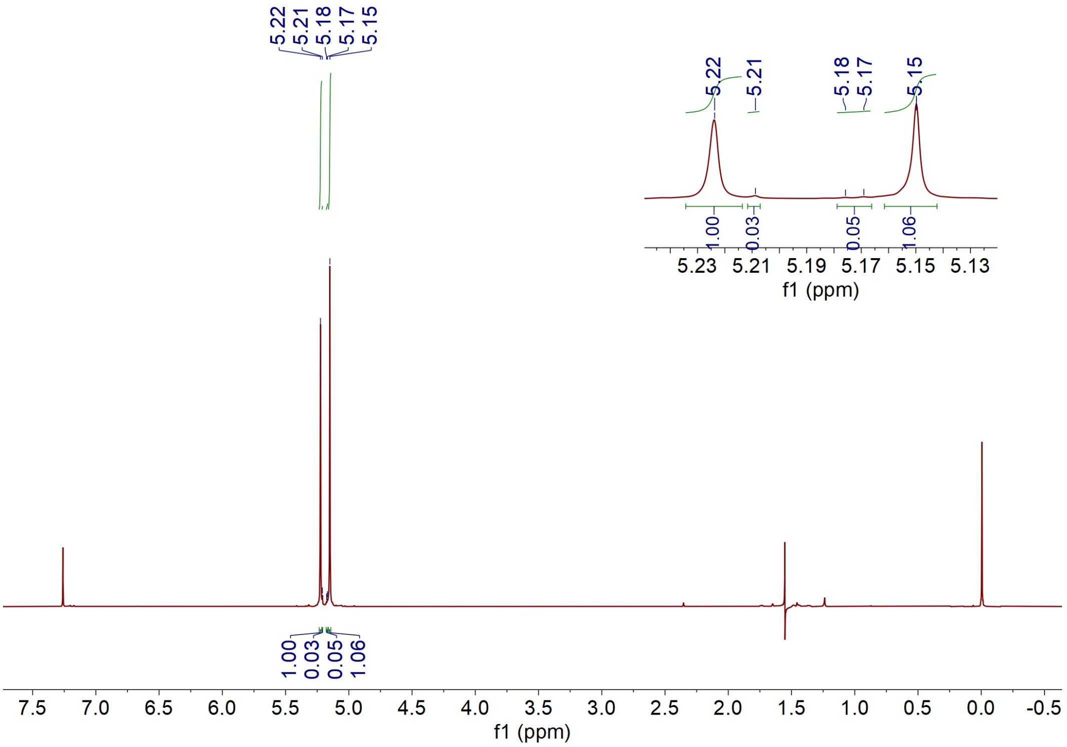

Fig. S24. Homonuclear decoupled ^{1}H NMR ($CDCl_3$, 25 °C) spectrum of heterotactic PLA (P_r = 0.97). PLA was produced by [*rac*-LA]/[(*R*)-**Sc1**]/[I] = 200/1/1 in THF.

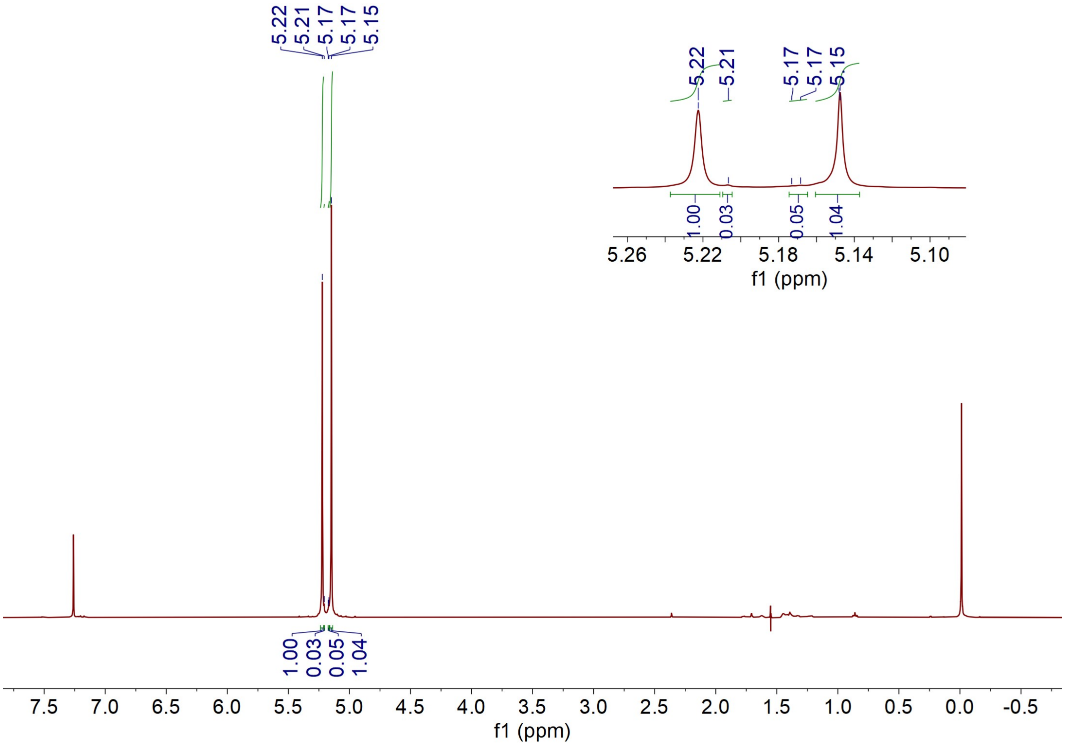

Fig. S25. Homonuclear decoupled ^{1}H NMR ($CDCl_3$, 25 °C) spectrum of heterotactic PLA (P_r = 0.97). PLA was produced by [*rac*-LA]/[(*S*)-**Sc1**]/[I] = 200/1/1 in THF.

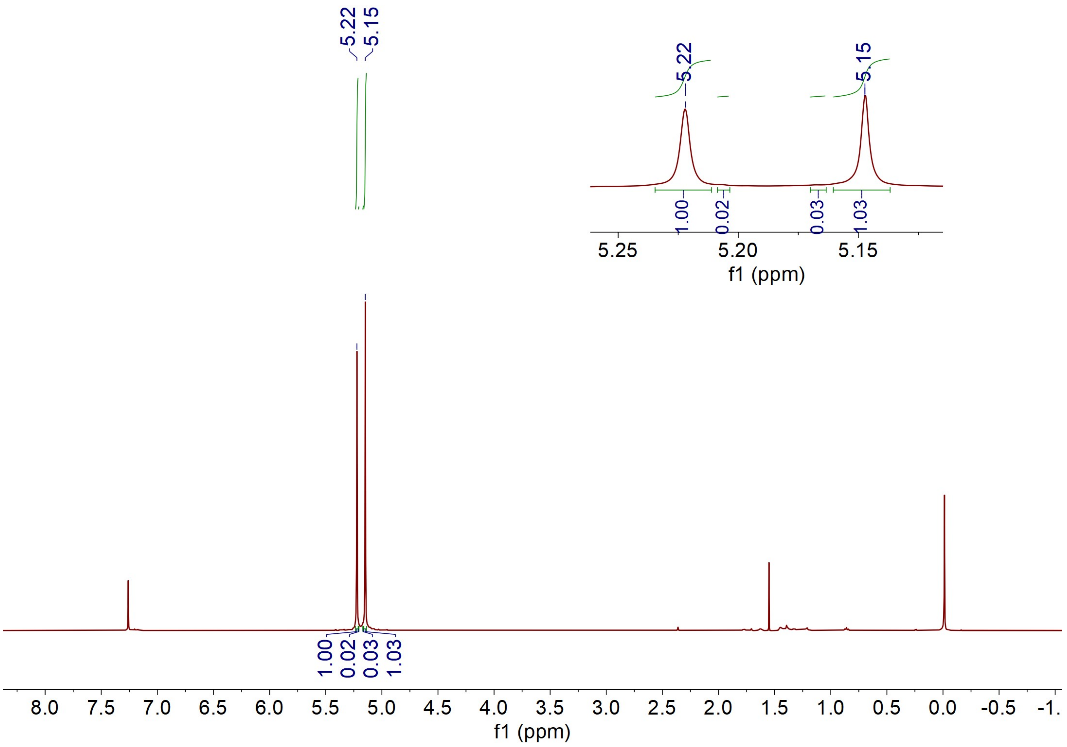

Fig. S26. Homonuclear decoupled ^{1}H NMR ($CDCl_3$, 25 °C) spectrum of heterotactic PLA (P_r = 0.98). PLA was produced by [*rac*-LA]/[*rac*-**Sc1**]/[I] = 200/1/1 in THF.

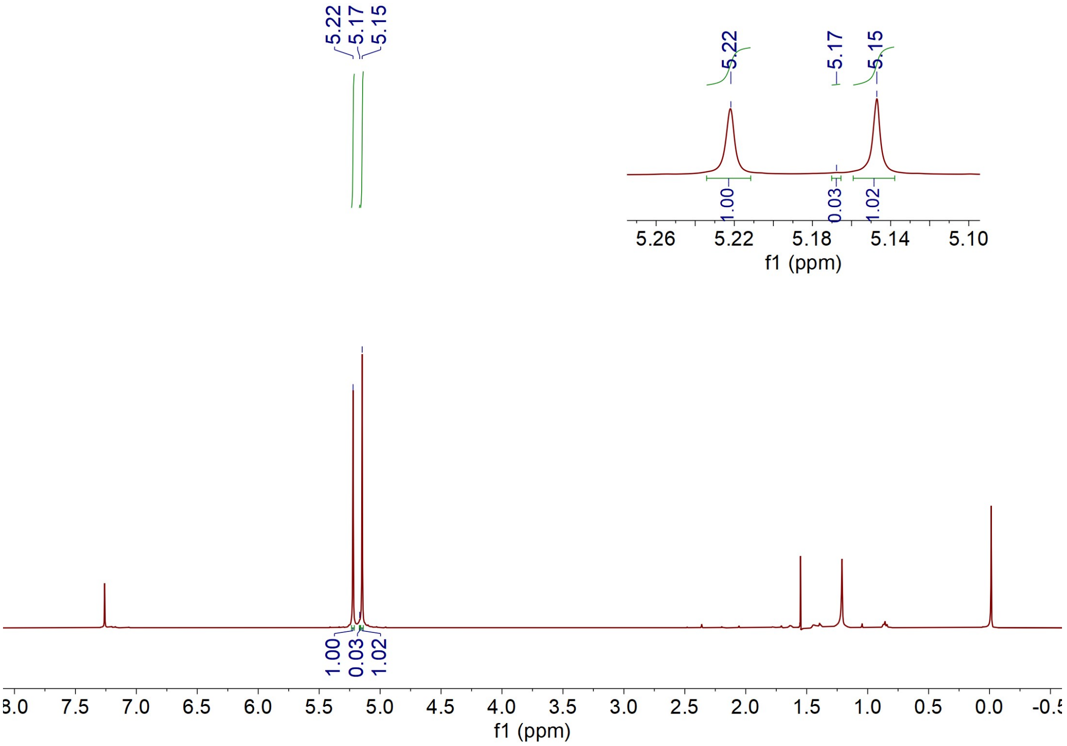

Fig. S27. Homonuclear decoupled ^{1}H NMR ($CDCl_3$, 25 °C) spectrum of heterotactic PLA (P_r = 0.99). PLA was produced by [*rac*-LA]/[*rac*-**Sc2**]/[I] = 200/1/1 in THF.

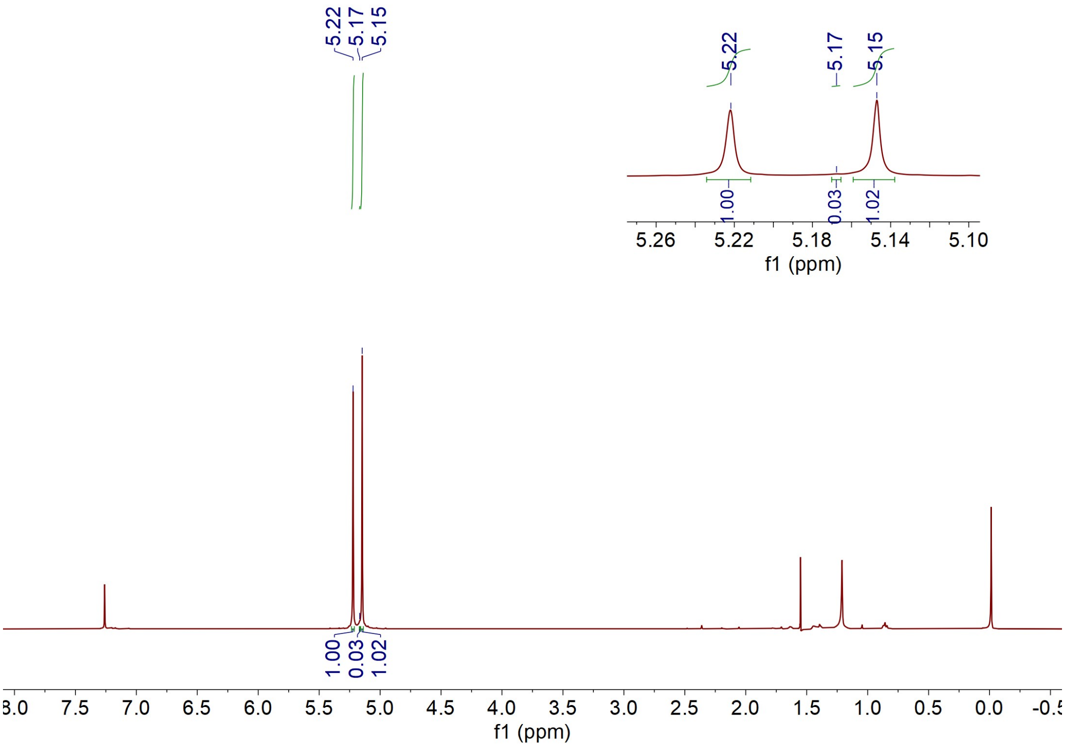

Fig. S28. Homonuclear decoupled ^{1}H NMR ($CDCl_3$, 25 °C) spectrum of heterotactic PLA (P_r = 0.99). PLA was produced by [*rac*-LA]/[*rac*-**Sc2**]/[I] = 500/1/1 in THF.

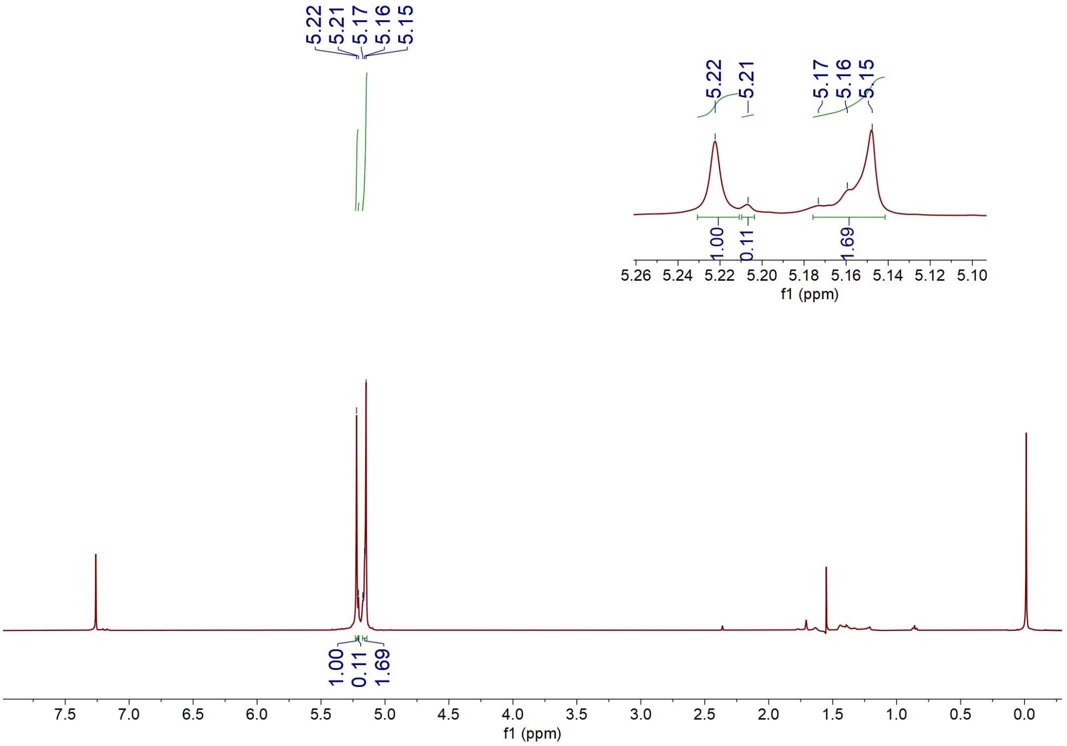

Fig. S29. Homonuclear decoupled ^{1}H NMR ($CDCl_3$, 25 °C) spectrum of heterotactic PLA (P_r = 0.85). PLA was produced by [*rac*-LA]/[(*R*)-**Sc1**]/[I] = 200/1/1, neat.

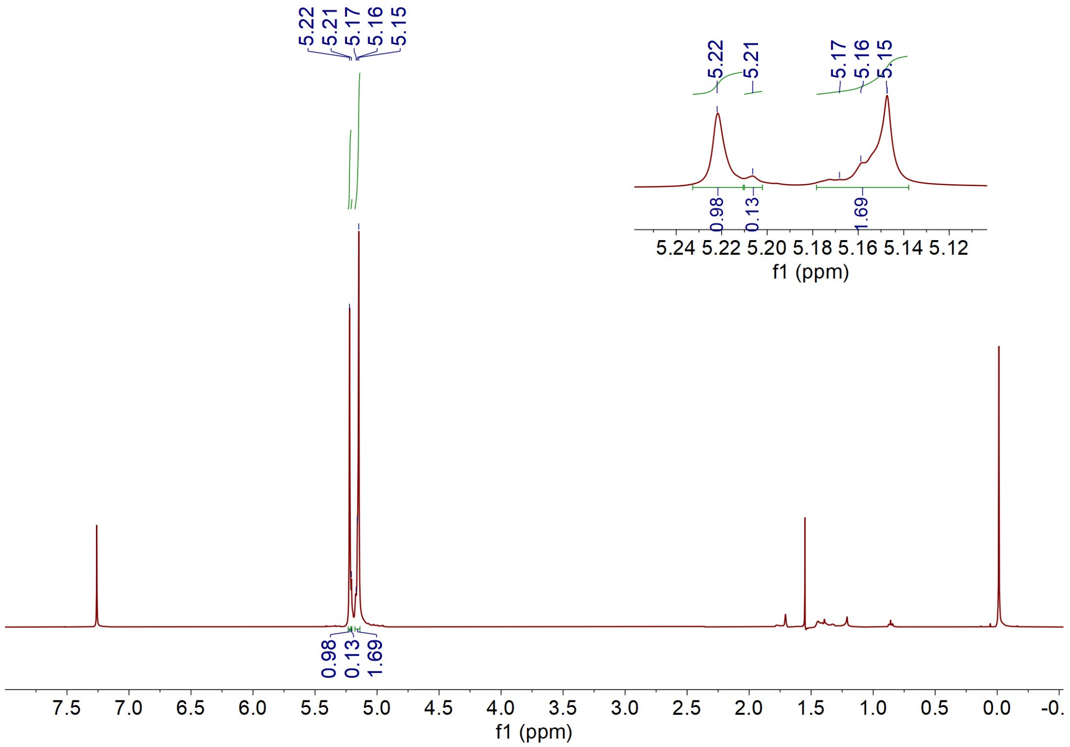

Fig. S30. Homonuclear decoupled ^{1}H NMR ($CDCl_3$, 25 °C) spectrum of heterotactic PLA (P_r = 0.85). PLA was produced by [*rac*-LA]/[*rac*-**Sc2**]/[I] = 1000/1/1 in THF, neat.

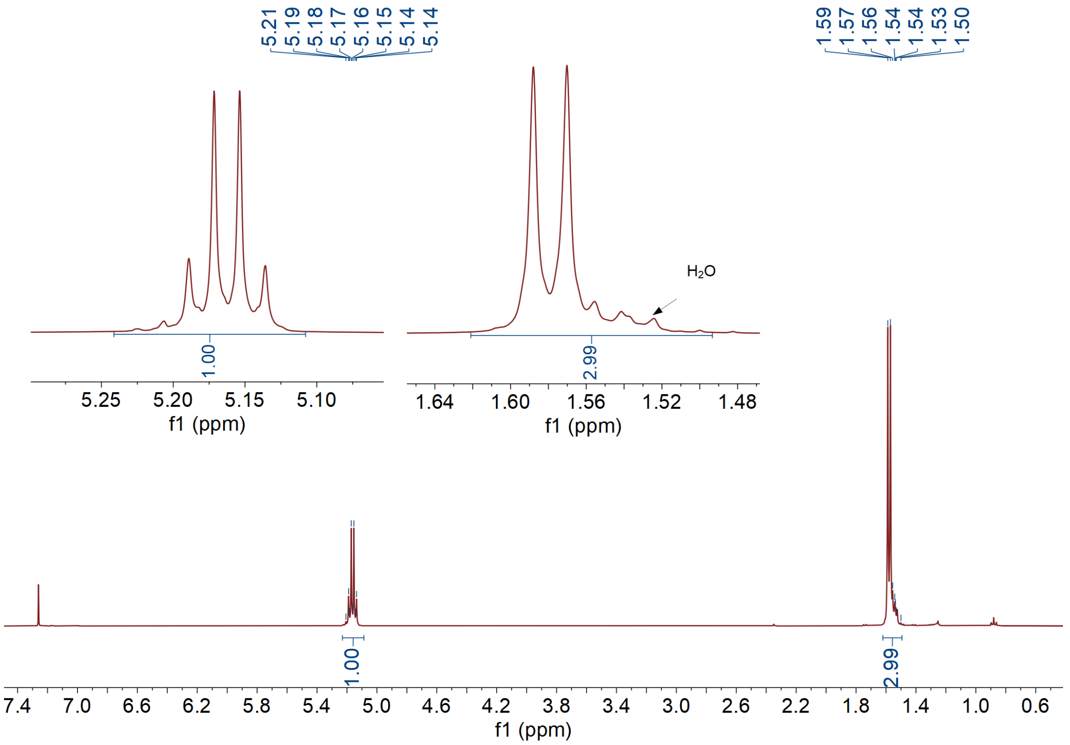

Figure S31 ^{1}H NMR ($CDCl_3$, 25 °C) spectrum of P(*meso*-LA-*grad*-LLA). P(*meso*-LA-*grad*-LLA) was produced by [L-LA]/[*meso*-LA]/[(*S*)-**Sc1**]/[I] = 190/10/1/1 inTHF.

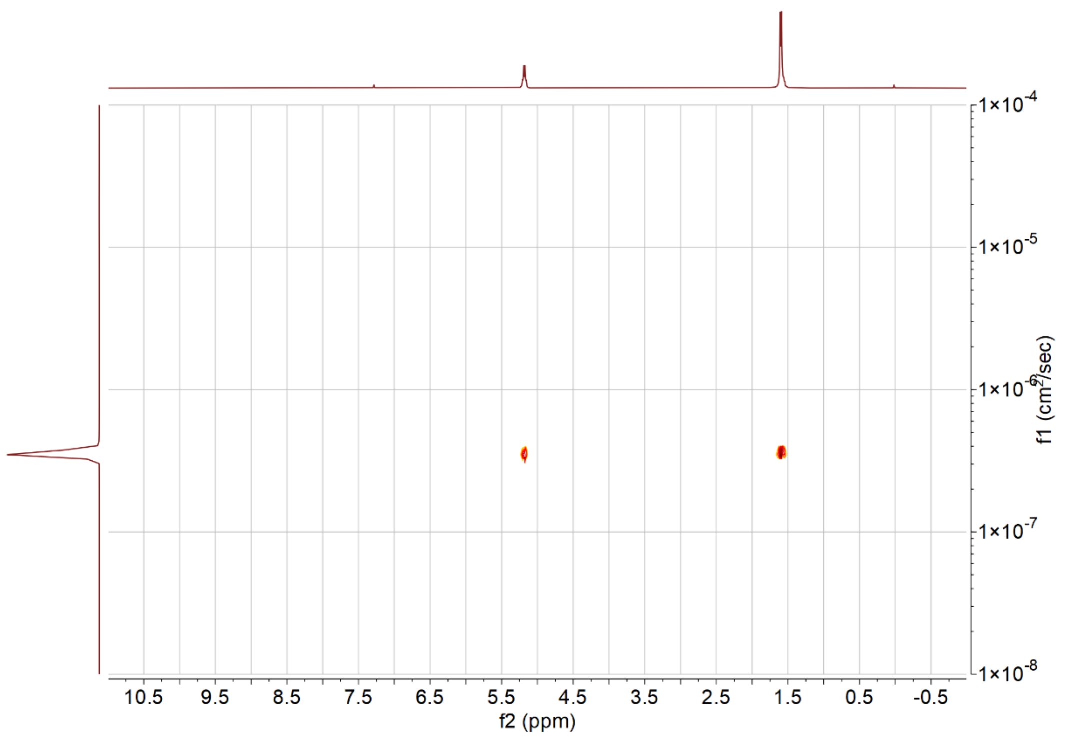

Figure S32. ^{1}H DOSY NMR (400 MHz, $CDCl_3$) spectrum of P(*meso*-LA-*grad*-LLA). P(*meso*-LA-*grad*-LLA) was obtained by[L-LA]/[*meso*-LA]/[(*S*)-**Sc1**]/[I] = 190//10/1/1 in THF.

SEC traces of PLA

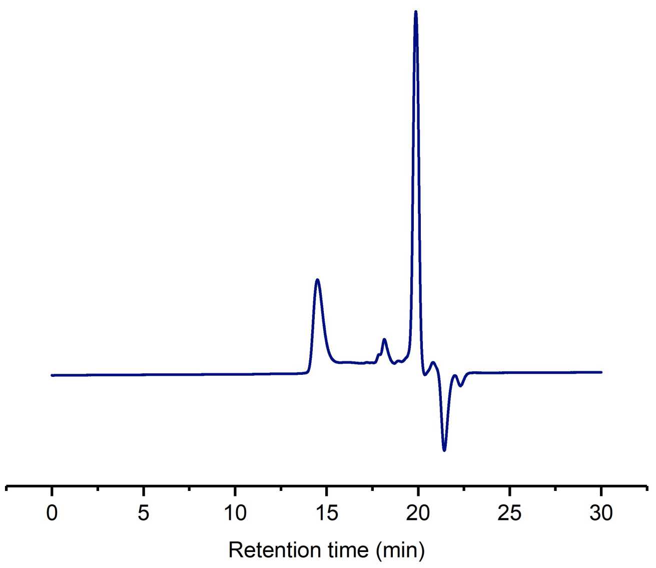

Fig. S33. SEC trace of syndiotactic PLA (P_r = 0.96, M_n = 26.1 kDa, $Đ$ = 1.05) obtained by [*meso*-LA]/[(*R*)-**Sc1**]/[I] = 200/1/1 in THF.

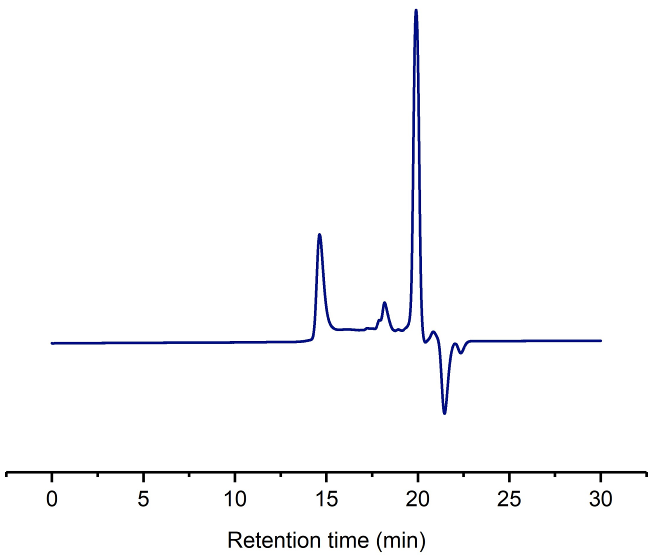

Fig. S34. SEC trace of syndiotactic PLA (P_r = 0.98, M_n = 25.6 kDa, *Đ* = 1.04) obtained by [*meso*-LA]/[*rac*-**Sc2**]/[I] = 200/1/1 in THF.

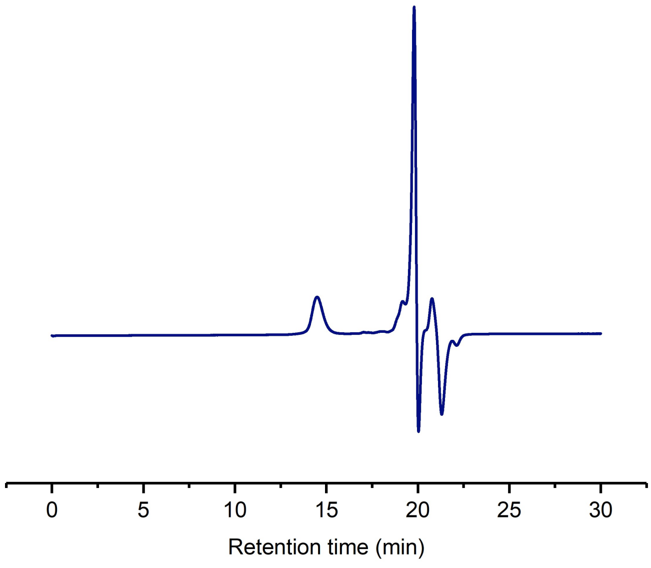

Fig. S35. SEC trace of syndiotactic PLA (P_r = 0.94, M_n = 21.8 kDa, *Đ* = 1.09) obtained by [*meso*-LA]/[(*R*)-**Sc1**]/[I] = 200/1/1, neat.

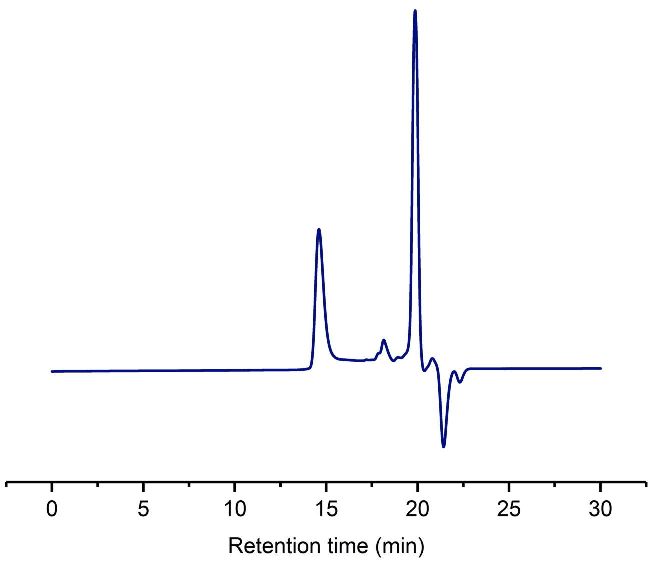

Fig. S36. SEC trace of heterotactic PLA (P_r = 0.97, M_n = 25.2 kDa, $Đ$ = 1.07) obtained by [*rac*-LA]/[(*R*)-**Sc1**]/[I] = 200/1/1 in THF.

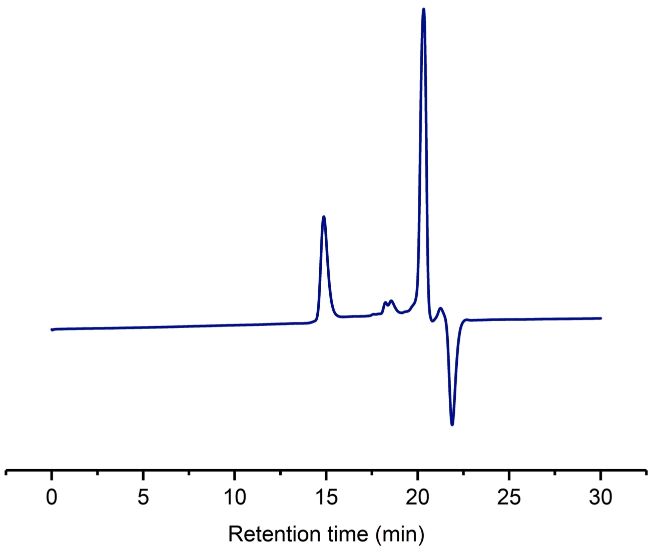

Fig. S37. SEC trace of heterotactic PLA (P_r = 0.99, M_n = 25.2 kDa, $Đ$ = 1.05) obtained by [*rac*-LA]/[*rac*-**Sc2**]/[I] = 200/1/1 in THF.

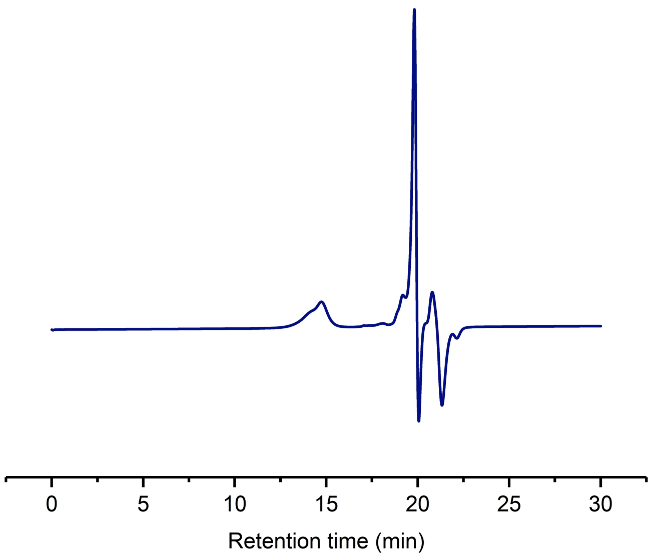

Fig. S38. SEC trace of heterotactic PLA (P_r = 0.85, M_n = 21.3 kDa, $Đ$ = 1.30) obtained by [*rac*-LA]/[(*R*)-**Sc1**]/[I] = 200/1/1, neat.

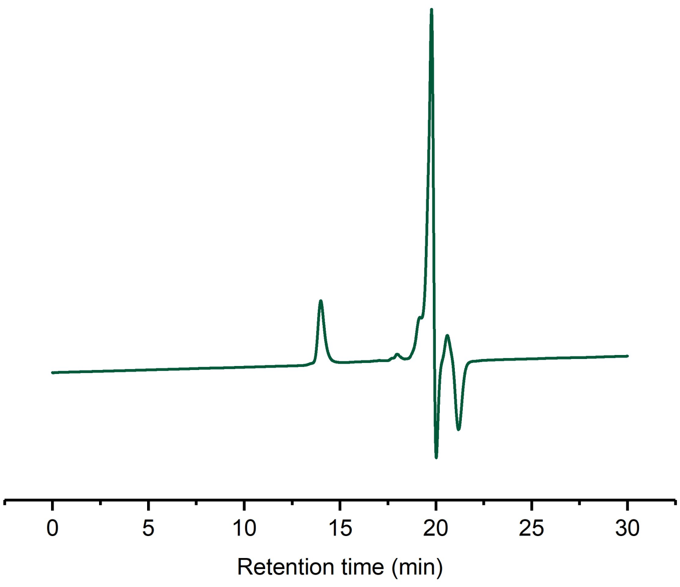

Fig. S39. SEC trace of P(*meso*-LA-*grad*-LLA) (M_n = 41.0 kDa, $Đ$ = 1.04). P(*meso*-LA-*grad*-LLA) synthesized from [L-LA]/[*meso*-LA]/[(*S*)-**Sc1**]/[I] = 180/20/1/1 in THF.

TGA curves for PLA

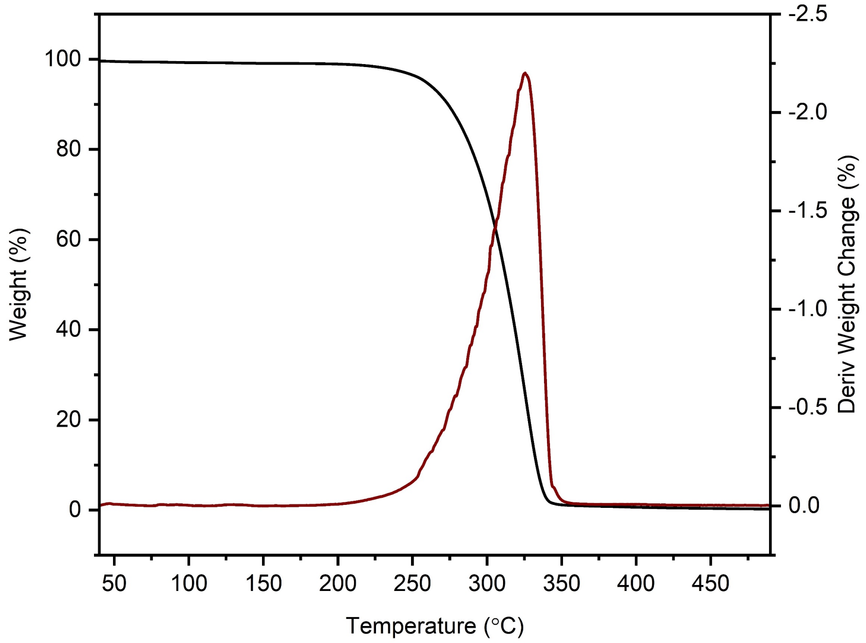

Fig. S40. TGA and DTG curves for syndiotactic PLA (P_r = 0.96) obtained by [*meso*-LA]/[(*R*)-**Sc1**]/[I] = 200/1/1 in THF, T_d = 259 °C, T_{max} = 337 °C.

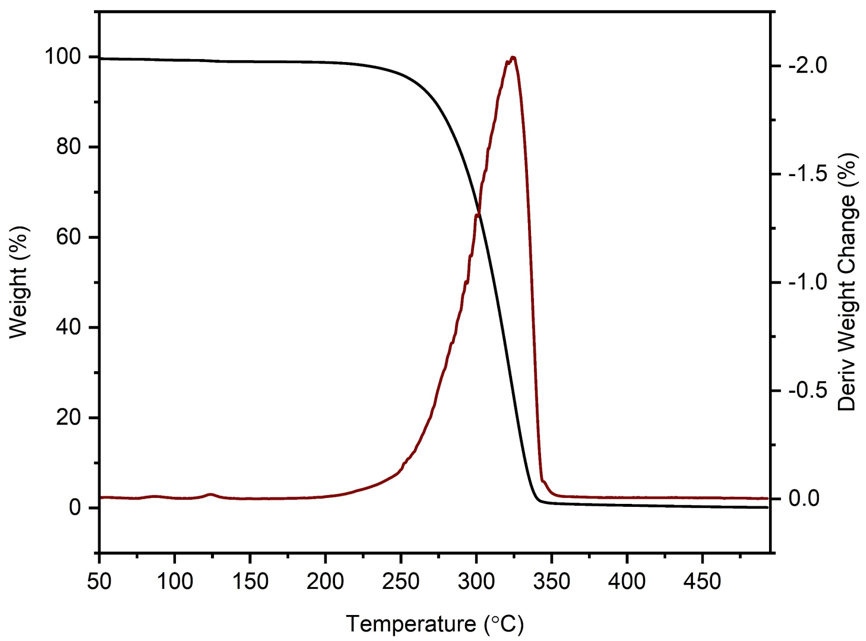

Fig. S41. TGA and DTG curves for syndiotactic PLA (P_r = 0.98) obtained by [*meso*-LA]/[*rac*-**Sc2**]/[I] = 200/1/1 in THF, T_d = 256 °C, T_{max} = 324 °C.

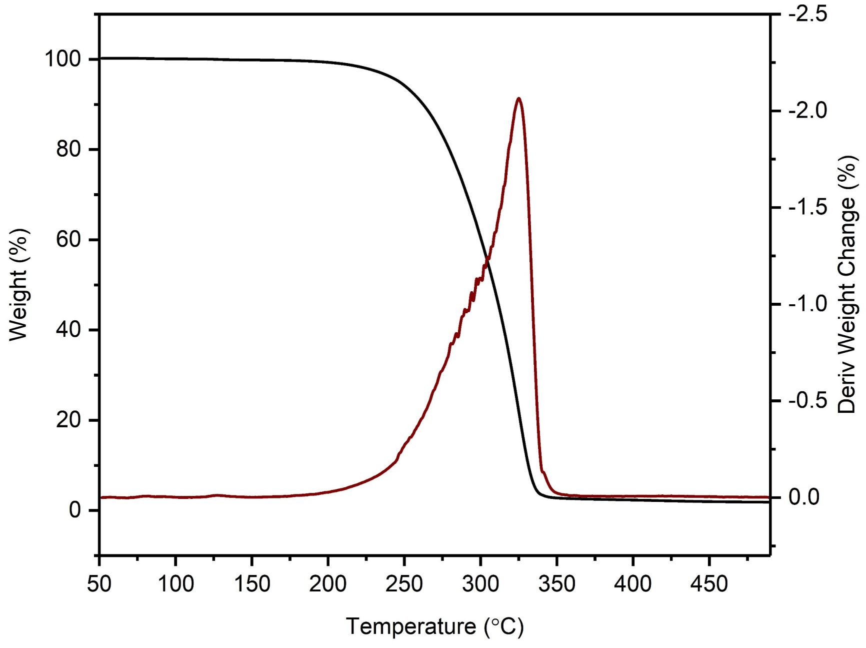

Fig. S42. TGA and DTG curves for syndiotactic PLA (P_r = 0.94) obtained by [*meso*-LA]/[(*R*)-**Sc1**]/[I] = 200/1/1, neat, T_d = 247 °C, T_{max} = 325 °C.

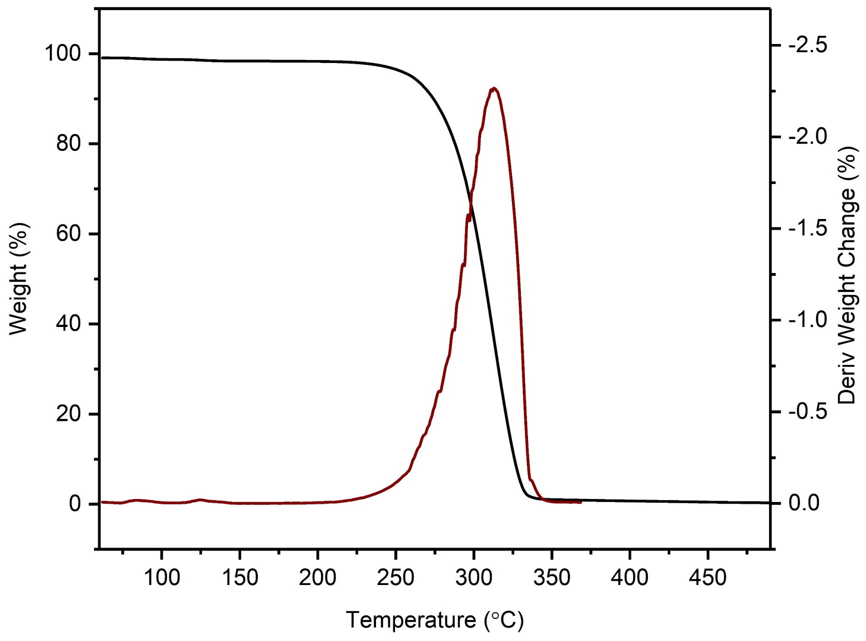

Fig. S43. TGA and DTG curves for syndiotactic PLA (P_r = 0.96) obtained by [*meso*-LA]/[(*R*)-**Sc1**]/[I] = 2000/1/1, neat, T_d = 260 °C, T_{max} = 312 °C.

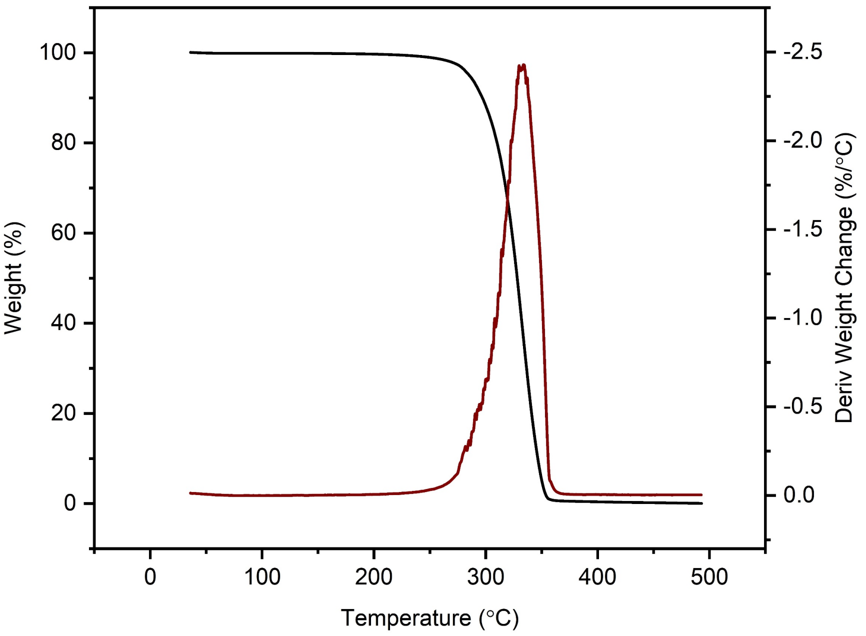

Fig. S44. TGA and DTG curves for heterotactic PLA (P_r = 0.98) obtained by [*rac*-LA]/[*rac*-**Sc1**]/[I] = 200/1/1 in THF, T_d = 285 °C, T_{max} = 334 °C.

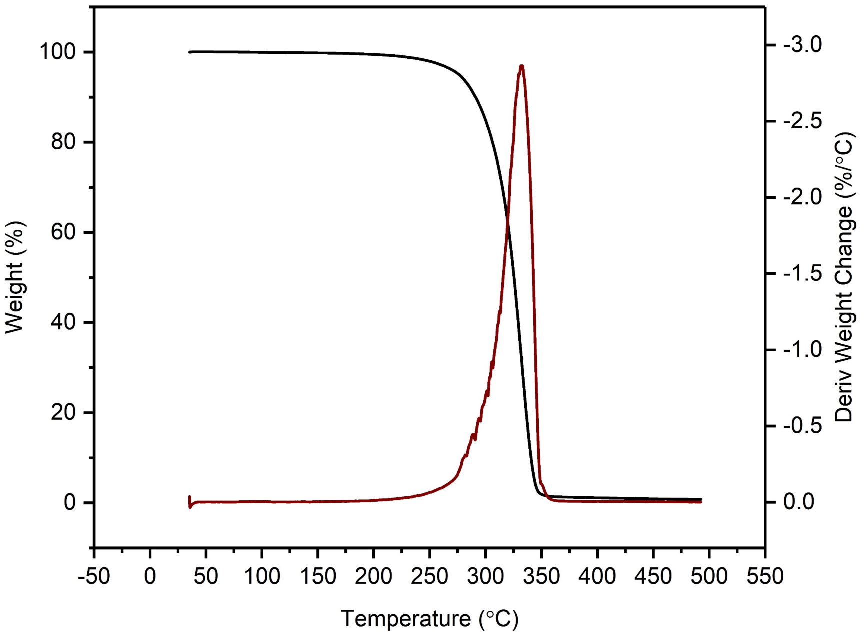

Fig. S45. TGA and DTG curves for heterotactic PLA (P_r = 0.99) obtained by [*rac*-LA]/[*rac*-**Sc2**]/[I] = 200/1/1 in THF, T_d = 276 °C, T_{max} = 332 °C.

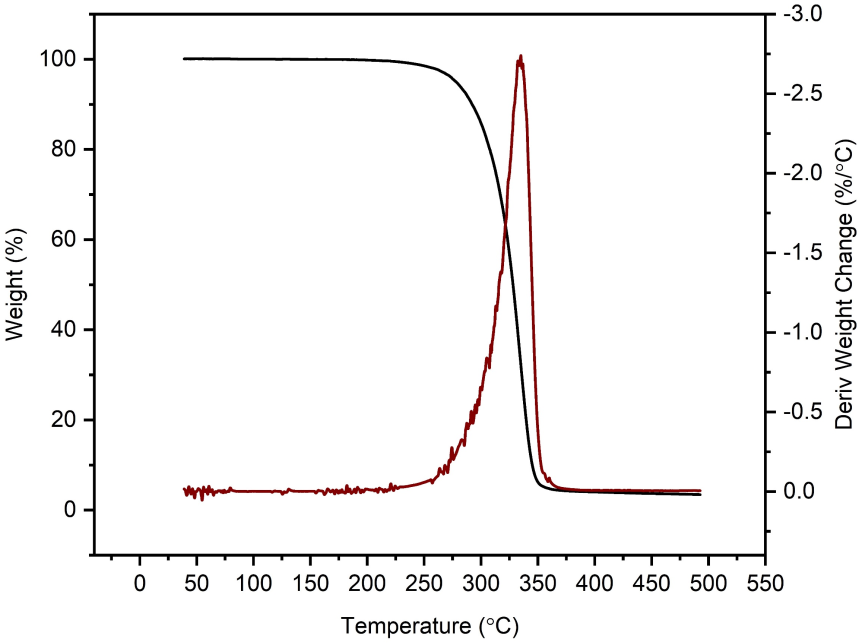

Fig. S46. TGA and DTG curves for heterotactic PLA (P_r = 0.99) obtained by [*rac*-LA]/[*rac*-**Sc2**]/[I] = 500/1/1 in THF, T_d = 278 °C, T_{max} = 335 °C.

DSC curves for PLA

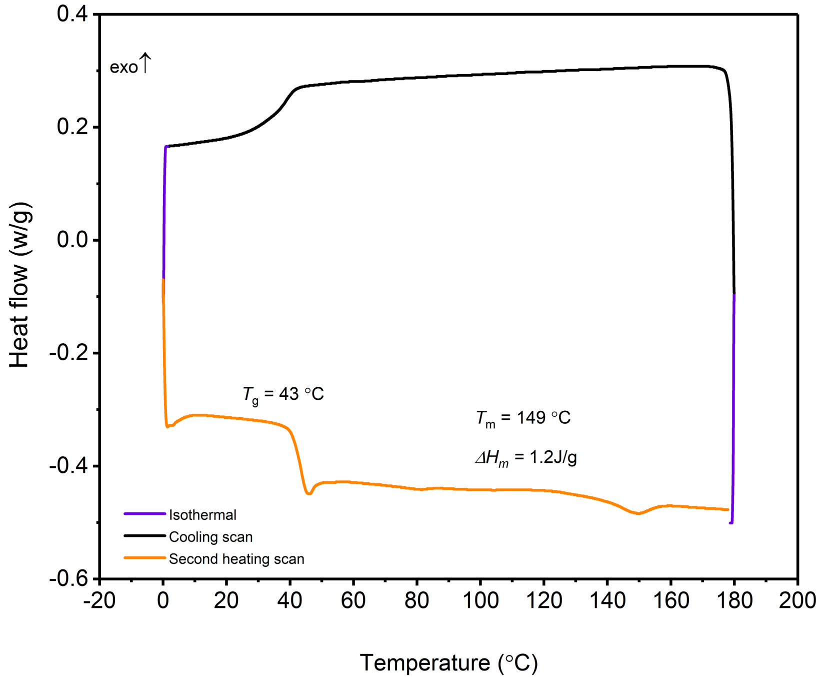

Fig. S47. DSC curves for syndiotactic PLA (P_r = 0.96). PLA was obtained by [*meso*-LA]/[(*R*)-**Sc1**]/[I] = 200/1/1 in THF, T_m = 149 °C.

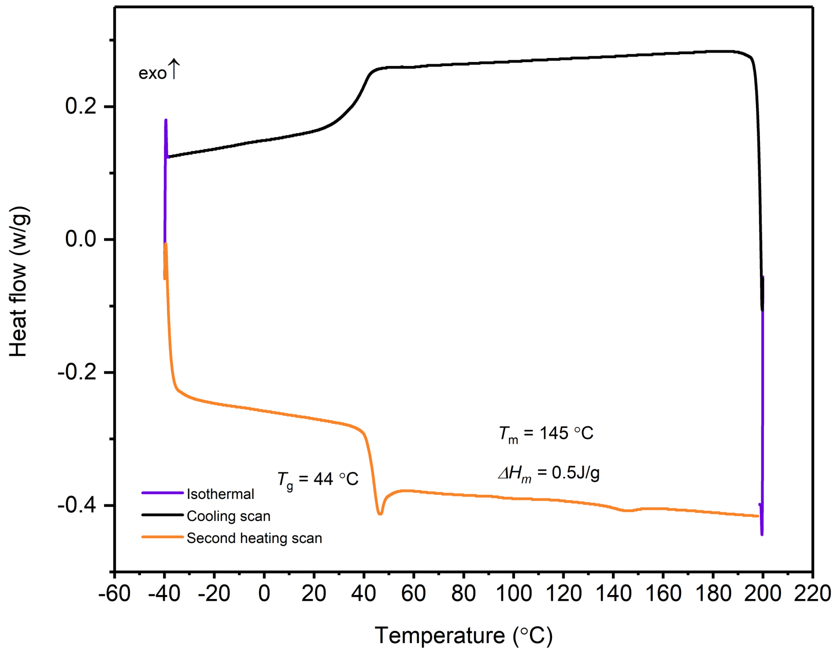

Fig. S48. DSC curves for syndiotactic PLA (P_r = 0.95). PLA was obtained by [*meso*-LA]/[(*R*)-**Sc1**]/[I] = 200/1/1 in TOL, T_m = 145 °C.

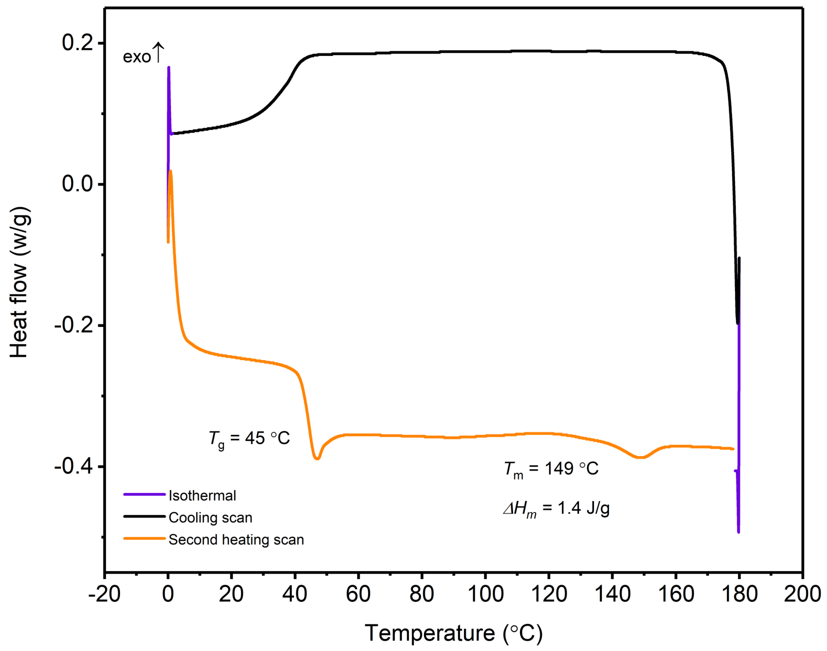

Fig. S49. DSC curves for syndiotac PLA ($P_{\rm r}$ = 0.96). PLA was obtained by [*meso*-LA]/[(*R*)-**Sc1**]/[I] = 500/1/1 in THF, $T_{\rm m}$ = 149 °C.

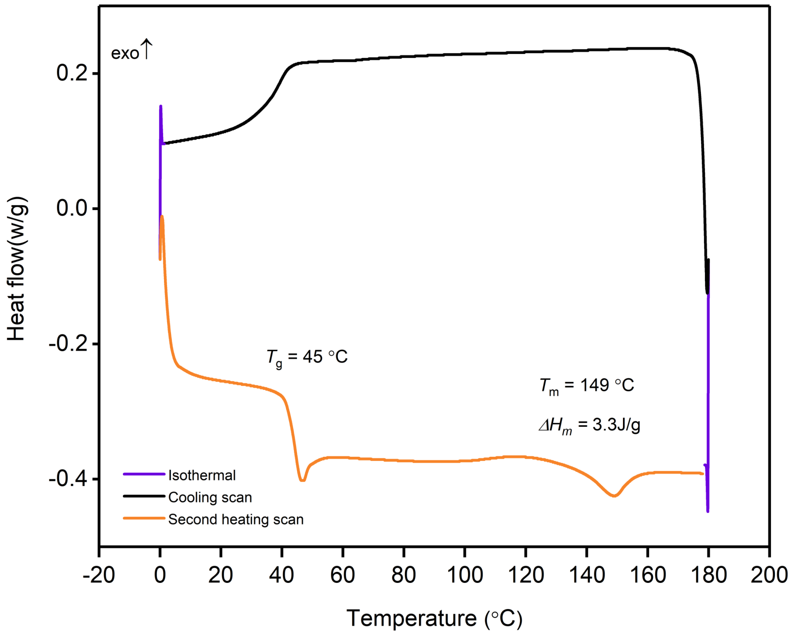

Fig. S50. DSC curves for syndiotactic PLA ($P_{\rm r}$ = 0.95). PLA was obtained by [*meso*-LA]/[(*S*)-**Sc1**]/[I] = 200/1/1 in THF, $T_{\rm m}$ = 149 °C.

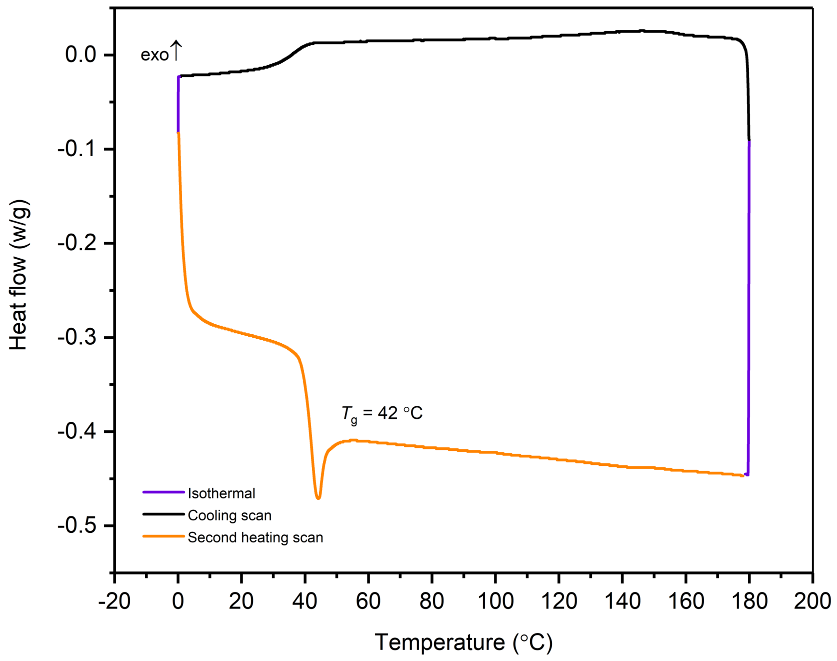

Fig. S51. DSC curves for syndiotactic PLA (P_r = 0.92). PLA was obtained by [*meso*-LA]/[(*R*)-**Sc2**]/[I] = 200/1/1 in THF.

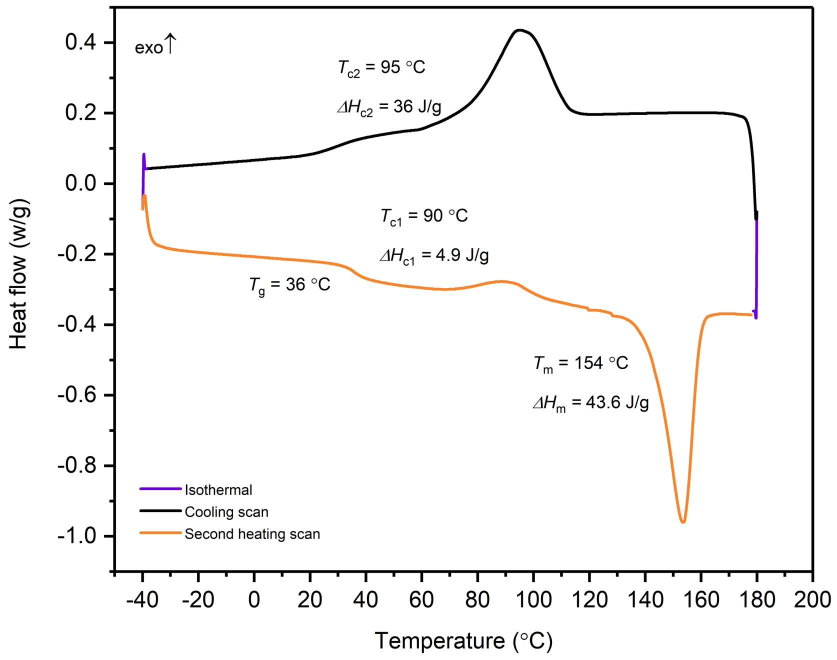

Fig. S52. DSC curves for syndiotactic PLA (P_r = 0.98). PLA was obtained by [*meso*-LA]/[*rac*-**Sc2**]/[I] = 50/1/1 in THF, T_m = 154 °C.

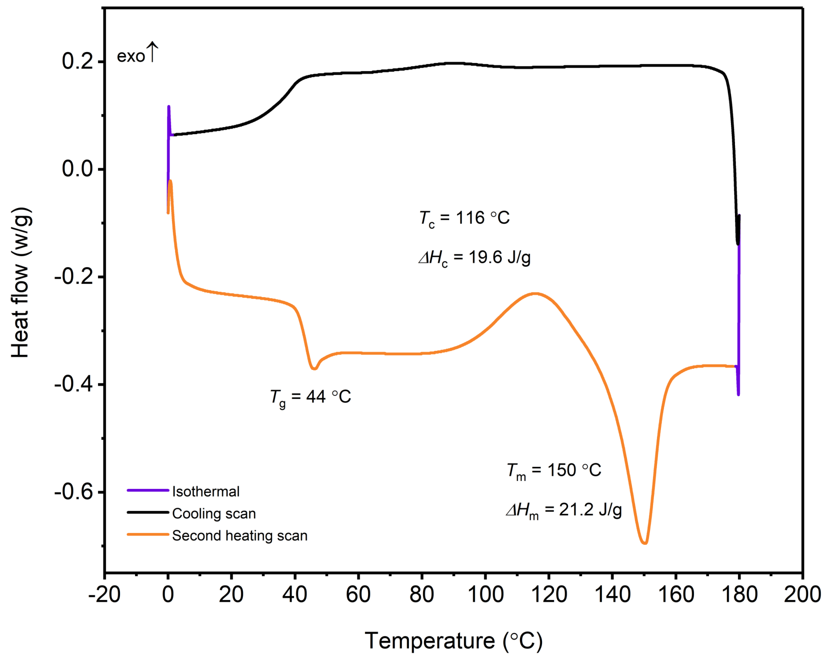

Fig. S53. DSC curves for syndiotactic PLA (P_r = 0.98). PLA was obtained by [*meso*-LA]/[*rac*-**Sc2**]/[I] = 200/1/1 in THF, T_m = 150 °C.

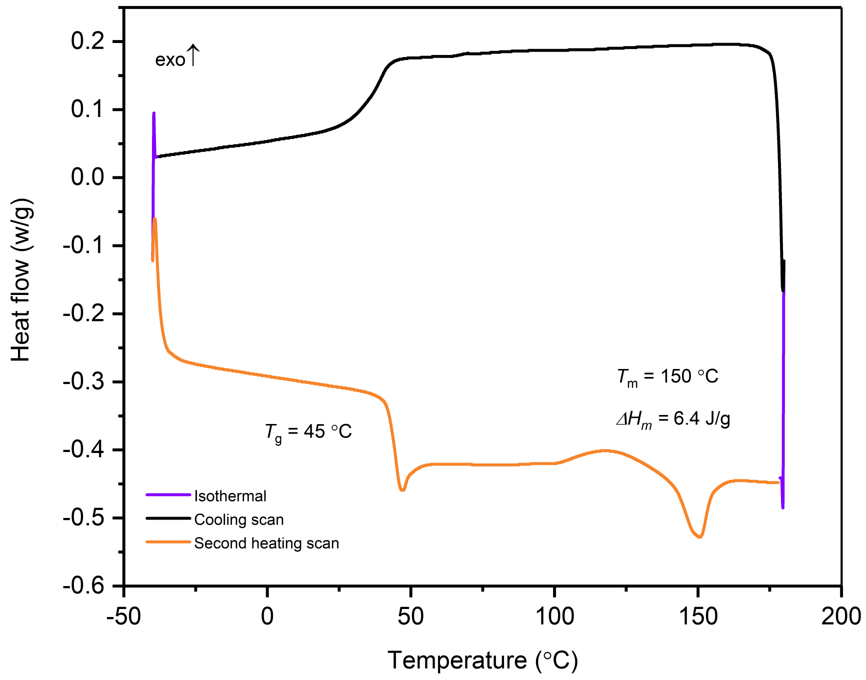

Fig. S54. DSC curves for syndiotactic PLA (P_r = 0.98). PLA was obtained by [*meso*-LA]/[*rac*-**Sc2**]/[I] = 500/1/1 in THF, T_m = 150 °C.

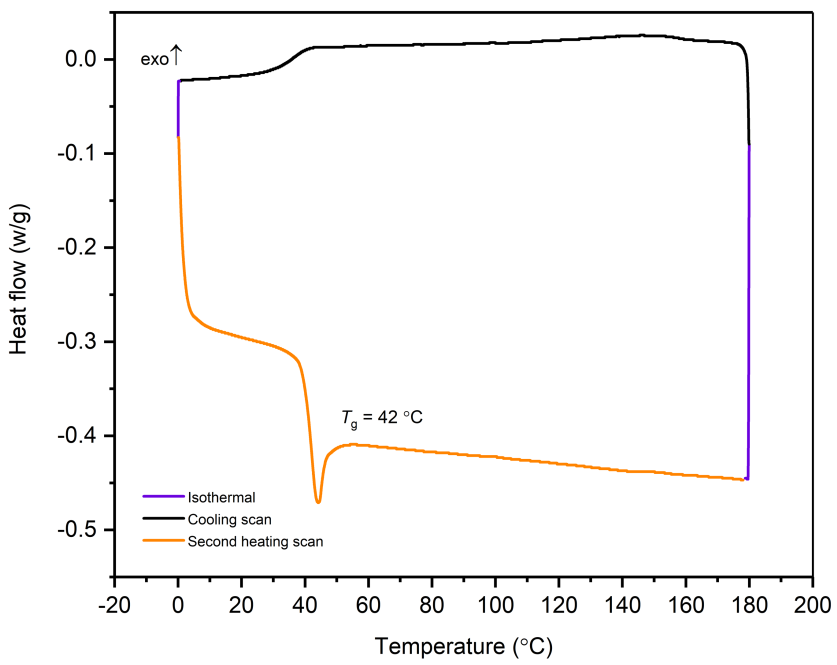

Fig. S55. DSC curves for syndiotactic PLA (P_r = 0.94). PLA was obtained by [*meso*-LA]/[(*R*)-**Sc1**]/[I] = 200/1/1, neat.

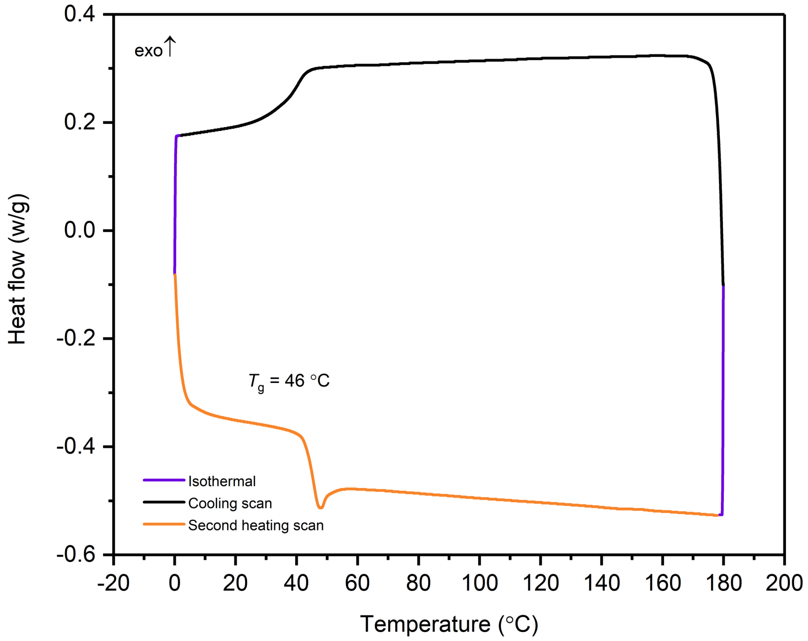

Fig. S56. DSC curves for syndiotactic PLA (P_r = 0.96). PLA was obtained by [*meso*-LA]/[(*R*)-**Sc1**]/[I] = 2000/1/1, neat.

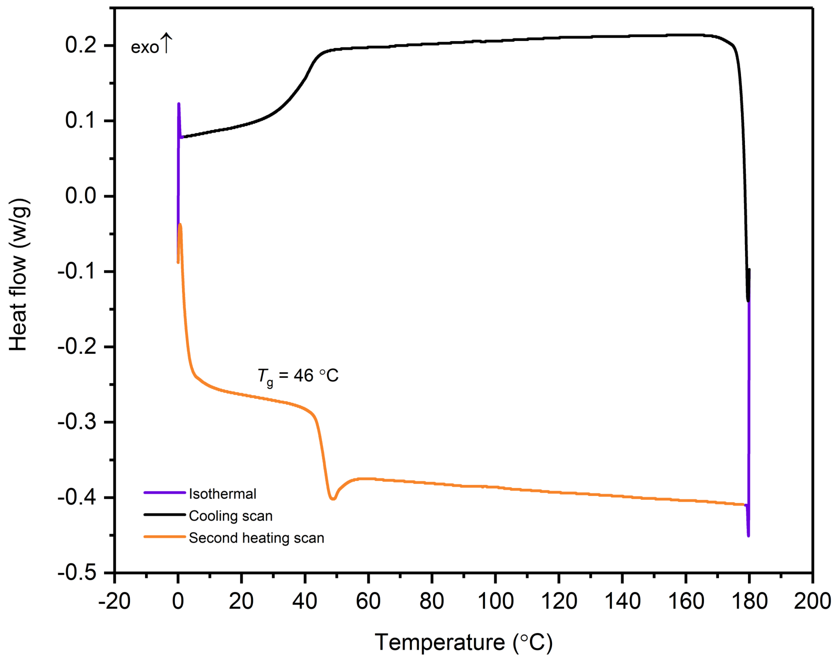

Fig. S57. DSC curves for heterotactic PLA (P_r = 0.97). PLA was obtained by [*rac*-LA]/[(*R*)-**Sc1**]/[I] = 200/1/1 in THF.

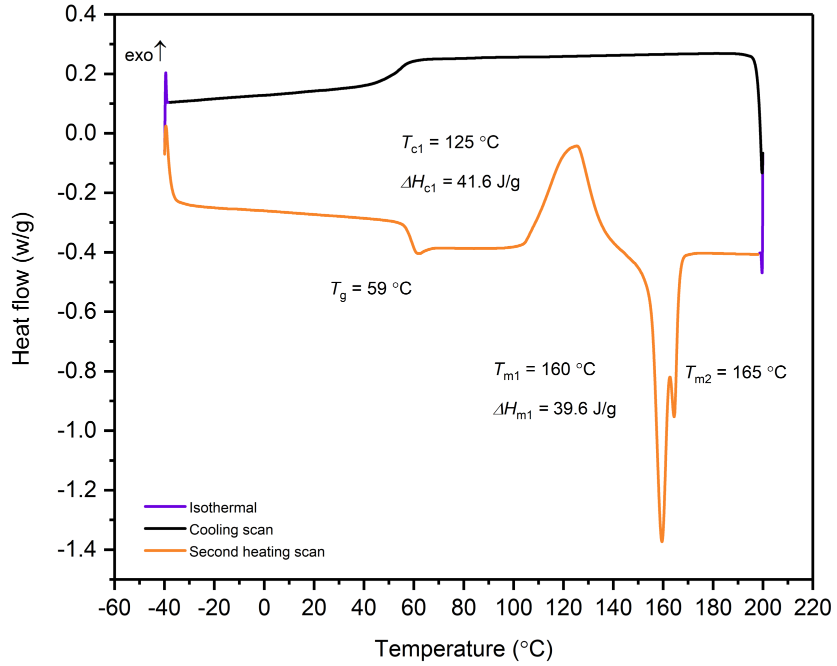

Fig. S58. DSC curves for PLLA. PLLA was obtained by [L-LA]/[(*S*)-**Sc1**]/[I] = 200/1/1 in THF, T_m = 160 °C/165 °C.

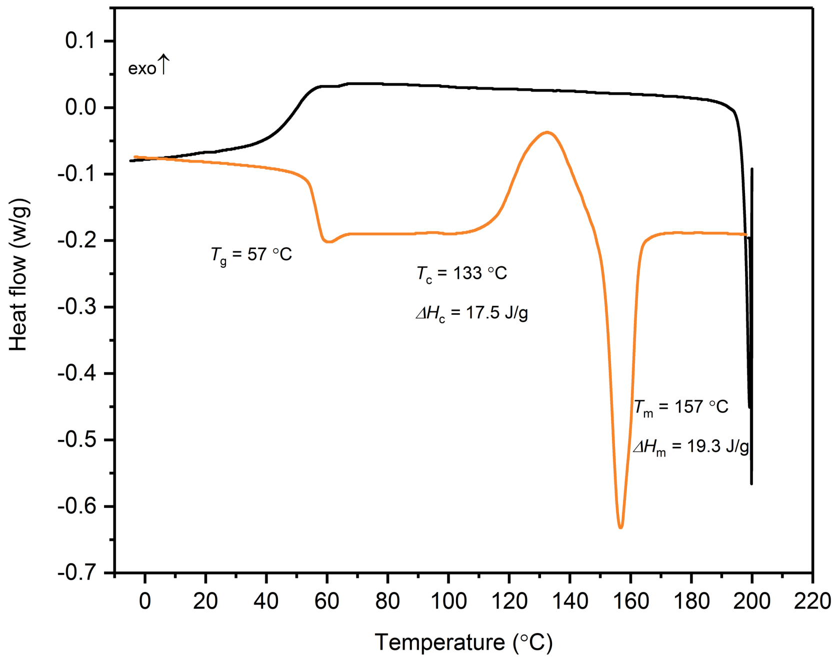

Fig. S59. DSC curves for P(*meso*-LA-*grad*-LLA). P(*meso*-LA-*grad*-LLA) was obtained by [L-LA]/[*meso*-LA]/[(*S*)-**Sc1**]/[I] = 198/2/1/1 in THF, T_m =157 °C.

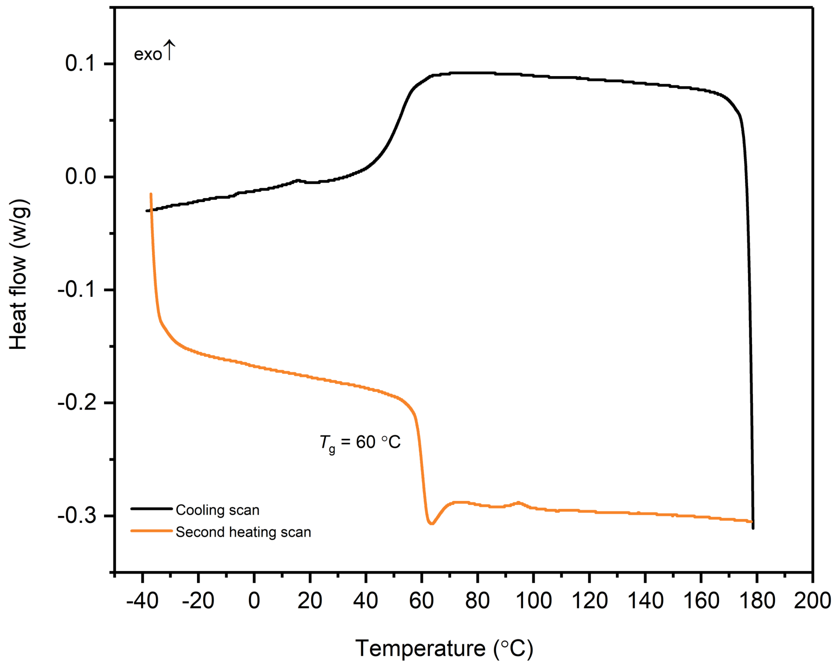

Fig. S60. DSC curves for P(*meso*-LA-*grad*-LLA). P(*meso*-LA-*grad*-LLA) was obtained by [LA]/[($Sn(Oct)_2$]/[BnOH] = 214000/214/1 at 180 °C, [L-LA]/[*meso*-LA] = 9/1, neat.

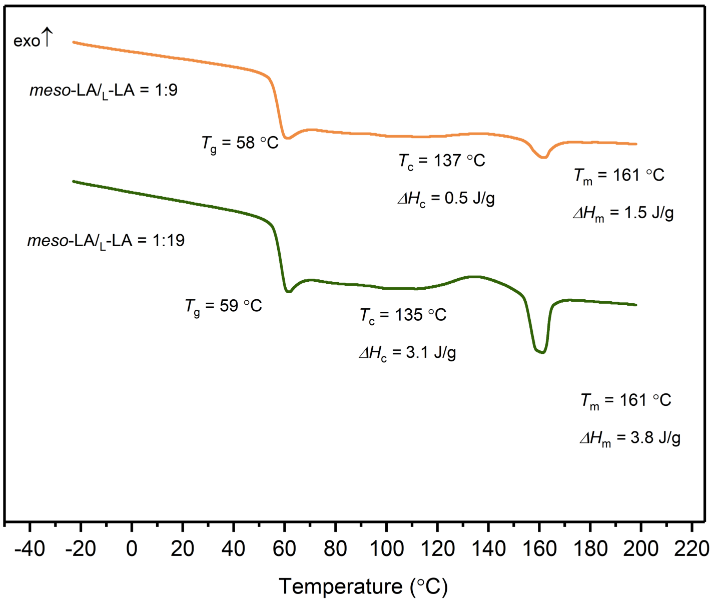

Fig. S61. DSC curves for P(*meso*-LA-*grad*-LLA). P(*meso*-LA-*grad*-LLA) was obtained by [LA]/[(*S*)-**Sc1**]/[I] = 600/1/1 in THF.

(a) *meso*-LA/L-LA = 1/9, T_m = 161 °C (orange line). (b) *meso*-LA/L-LA = 1/19, T_m = 161 °C (green line).

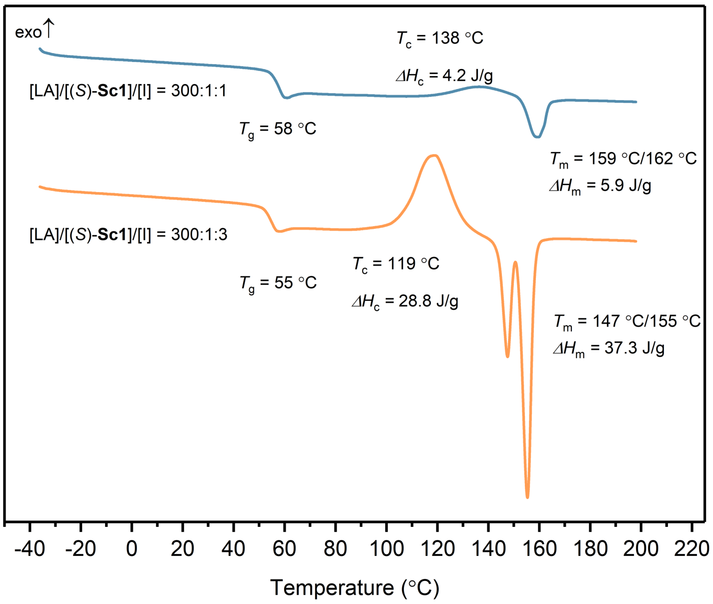

Fig. S62. DSC curves for P(*meso*-LA-*grad*-LLA). P(*meso*-LA-*grad*-LLA) was catalyzed by (*S*)-**Sc1** in THF, *meso*-LA/L-LA = 1/9.

(a) [LA]/[(*S*)-**Sc1**]/[I] = 300/1/1, T_m = 159 °C/161 °C (blue line). (b) [LA]/[(*S*)-**Sc1**]/[I] = 300/1/3, T_m = 147 °C/155 °C (orange line).

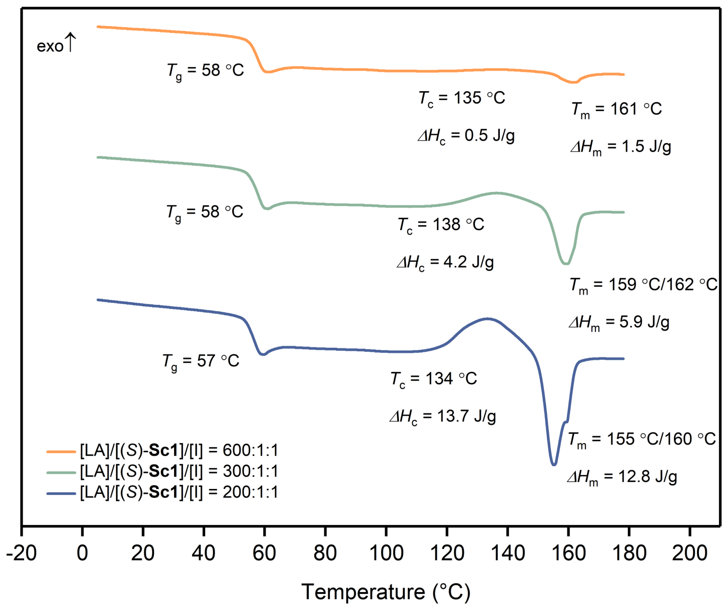

Fig. S63. DSC curves for P(*meso*-LA-*grad*-LLA). P(*meso*-LA-*grad*-LLA) was catalyzed by (*S*)-**Sc1** in THF, *meso*-LA/L-LA = 1/9.
(a) [LA]/[(*S*)-**Sc1**]/[I] = 600/1/1, T_m = 161 °C (orange line). (b) [LA]/[(*S*)-**Sc1**]/[I] = 300/1/1, T_m = 159 °C/162 °C (green line). (c) [LA]/[(*S*)-**Sc1**]/[I] = 200/1/1, T_m = 155 °C/160 °C (blue line).

Chemical Recycling to Monomer (CRM)

General procedure for the CRM of polymers in dilute solution

PLLA and *rac*-**Sc2** were mixture and dissolved in 0.5 mL THF. The solvent was then removed under vacuumed. The left dry mixture was equipped with a sublimation setup (cooling temperature: −20 °C) and stirred at 180−200 °C under 0.8 mbar vacuum for a certain time. At the end of the reaction, the setup was cooled to room temperature and transferred to the glovebox. The sublimate was collected, weighted, and characterized by ^{1}H NMR and HPLC spectroscopy.

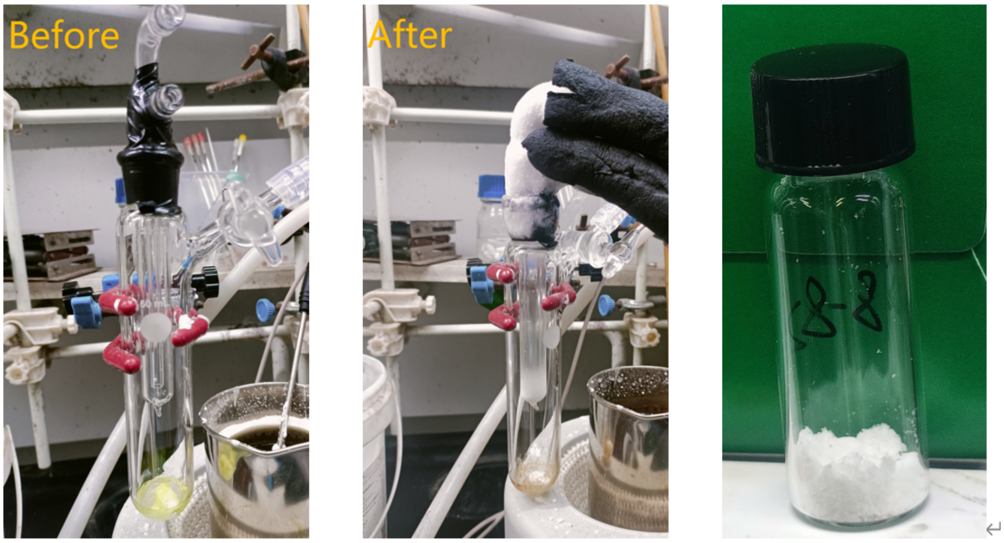

Fig. S64. Thermal depolymerization of PLLA to monomer via sublimation.

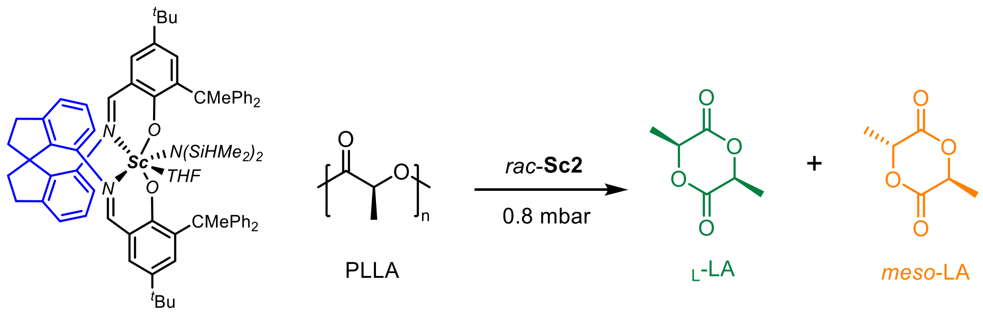

Table S3. Results of bulk thermal chemical recycling PLLA using spiro-salen catalyst *rac*-**Sc2**.[a]

Entry	catalyst	Ratio. (mol%)	Temp. (°C)	Time (h)	Monomer Yield[b] (%)	*meso*-LA[b] (%)	L-LA[c] (%)	ee[c] (%)
1	*rac*-**Sc2**	0	200	10	5	N. D.	N. D.	N. D.
2	*rac*-**Sc2**	0.1	200	11.5	80	1	>99	>99.5
3	*rac*-**Sc2**	0.2	200	7.5	93	2	99	>99.5
4	*rac*-**Sc2**	0.2	200	10.5	99	2	99	>99.5
5	*rac*-**Sc2**	0.2	180	11	80	1	>99	>99.5
6[d]	$Sc(OTf)_3$	0.2	200	9	16	2	95	93

[a] Reaction conditions: PLLA was produced by $Sn(Oct)_2$(M_n = 11.6 kDa, $Đ$ = 1.71, T_m = 167 °C/171 °C, $T_{d,5\%}$ = 329 °C), the cooling temperature was −20 °C. [b] Determined by the weight of the sublimate and the purity of recycled monomer determined by ^{1}H NMR spectroscopy. [c] The percentage of L-LA in sublimate product was determined by HPLC spectroscopy. [d] Reaction conditions : Commercial PLLA (M_n = 57.7 kDa, $Đ$ =1.61 T_m = 157 °C/164 °C, $T_{d,5\%}$ = 336 °C) was purchased from NatureWorks, the cooling temperature was −20 °C

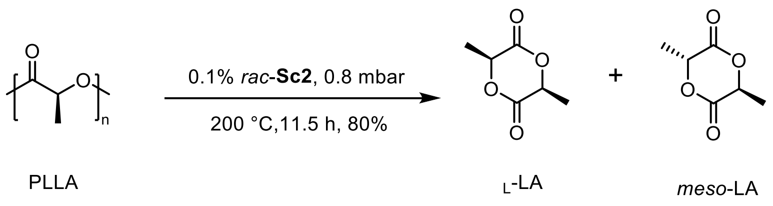

$$meso\text{-LA\%} = \frac{1\times2}{160.41} \times 100\% = 1.2\%$$

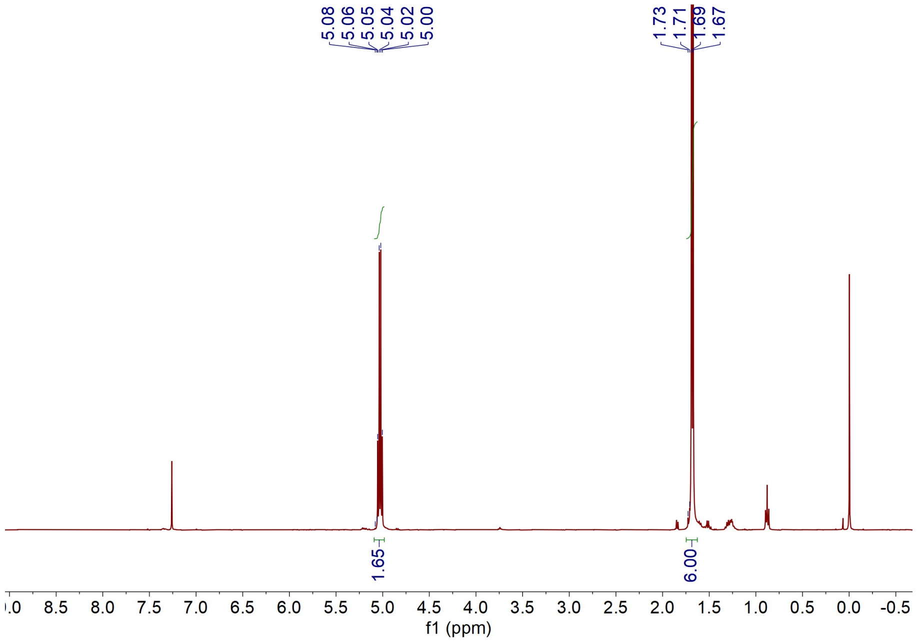

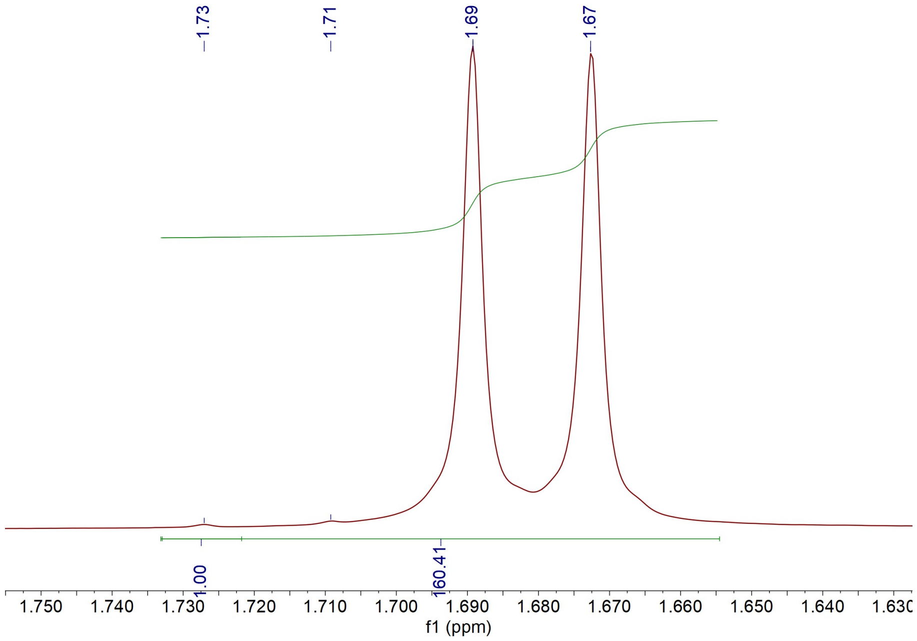

Fig. S65. ^{1}H NMR ($CDCl_3$, 25 °C) spectrum of recycled LA isolated from depolymerization of PLLA by *rac*-**Sc2**, [*rac*-**Sc2**]$_0$/[PLLA]$_0$ = 1/1000, 200 °C, 11.5 h.

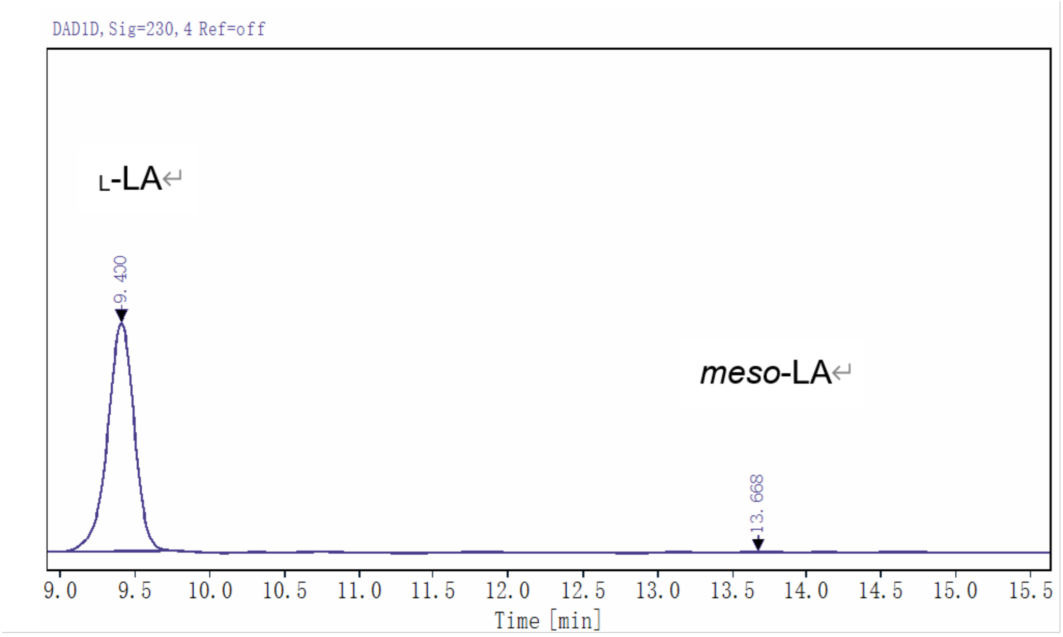

LA	Time (min)	Area	Height	width	Area (%)
L-LA	9.400	0.8167	829.9346	65.8495	99.6017
meso-LA	13.668	0.4451	3.3185	0.2444	0.3983

Fig. S66. HPLC chromatograms of recycled LA, [*rac*-**Sc2**]$_0$/[PLLA]$_0$ = 1/1000, 200 °C,11.5 h. Column, Chiralpak IA; flow rate, 1 mL min^{-1}; eluent, hexane-isopropanol = 90/10; detector, UV (230 nm); temperature, 35 °C.

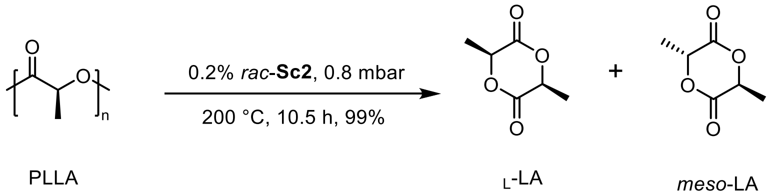

$$meso\text{-LA}\% = \frac{1\times2}{86.12} \times 100\% = 2.3\%$$

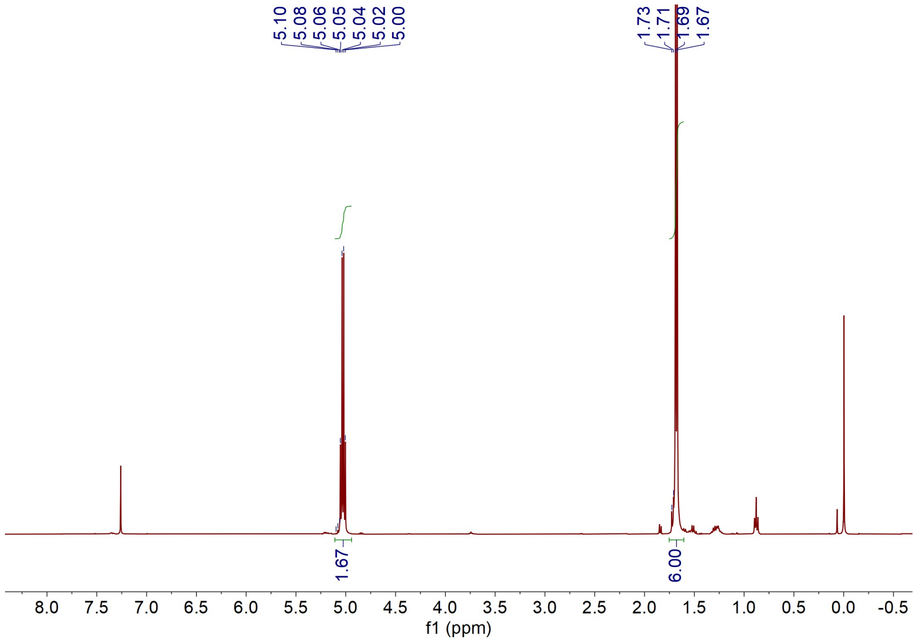

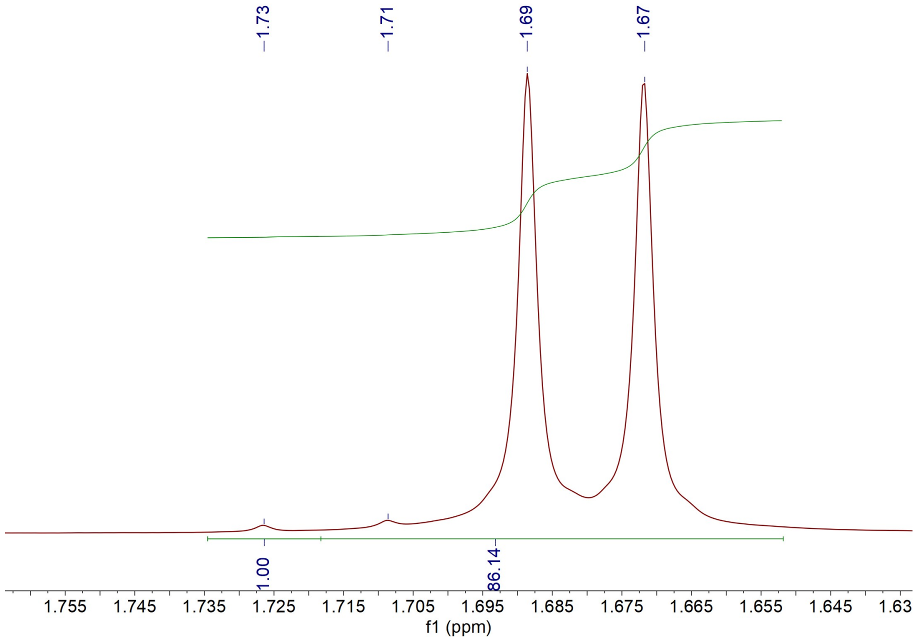

Fig. S67. ^{1}H NMR ($CDCl_3$, 25 °C) spectrum of recycled LA isolated from depolymerization of PLLA from mixtures of *rac*-**Sc2** and PLLA, [*rac*-**Sc2**]$_0$/[PLLA]$_0$ = 1/500, 200 °C, 10.5 h.

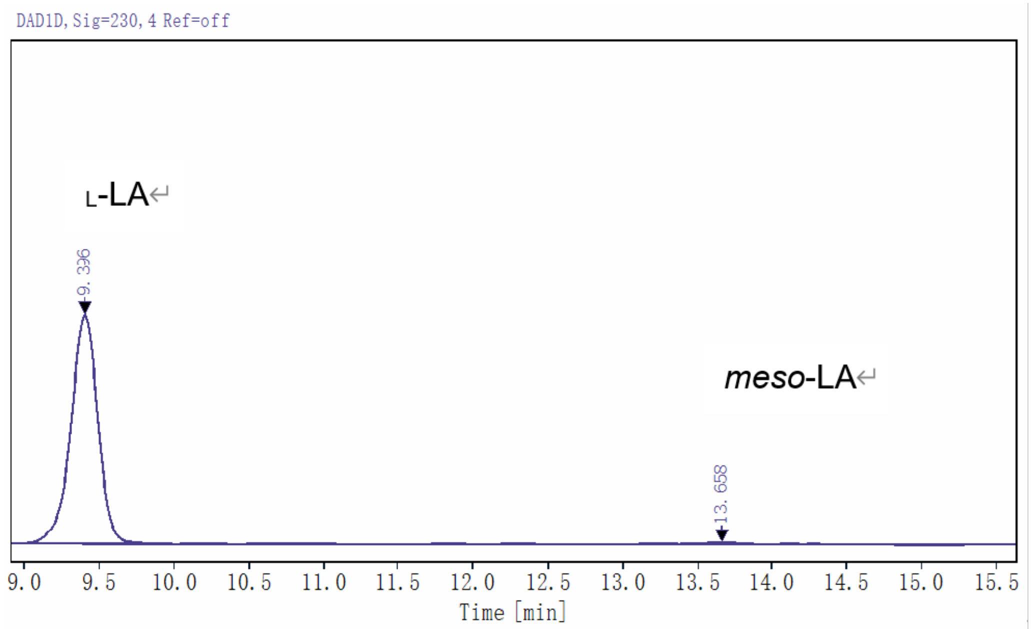

LA	Time (min)	Area	Height	width	Area (%)
L-LA	9.396	1.0168	881.0166	69.2357	99.1722
meso-LA	13.658	0.5479	7.3542	0.5191	0.8278

Fig. S68. HPLC chromatograms of recycled LA, [*rac*-**Sc2**]$_0$/[PLLA]$_0$ = 1/500, 200 °C,10.5 h. Column, Chiralpak IA; flow rate, 1 mL min^{-1}; eluent, hexane-isopropanol = 90/10; detector, UV (230 nm); temperature, 35 °C.

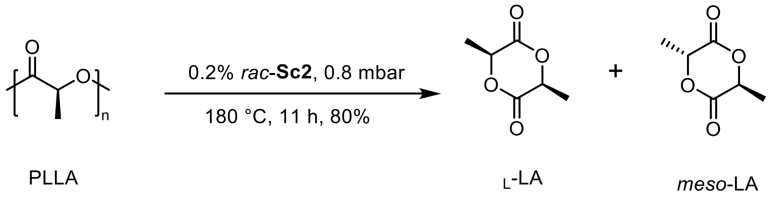

$$meso\text{-LA}\% = \frac{1\times2}{292.27}\times 100\% = 0.7\%$$

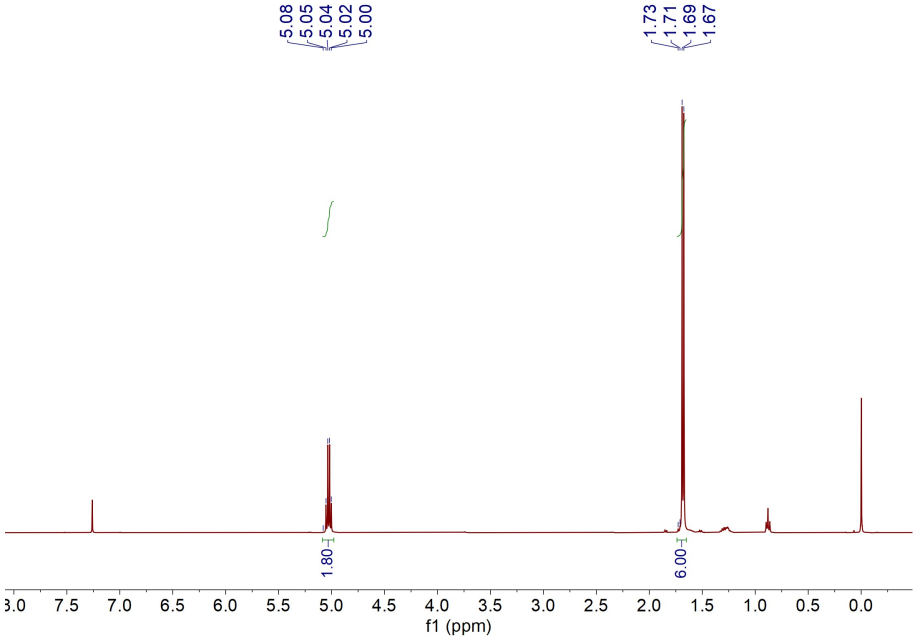

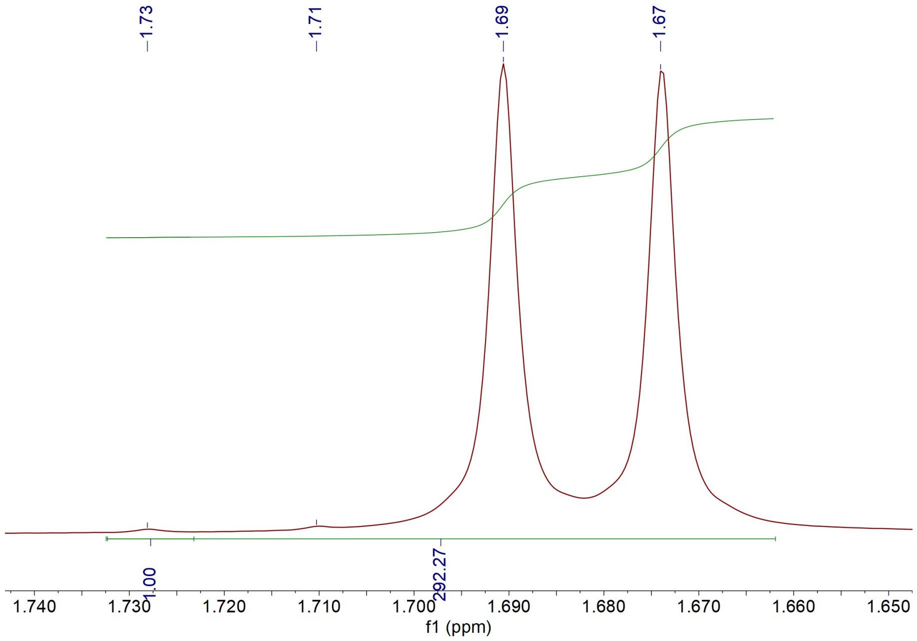

Fig. S69. ^{1}H NMR ($CDCl_3$, 25 °C) spectrum of recycled LA isolated from depolymerization of PLLA from mixtures of *rac*-**Sc2** and PLLA, [*rac*-**Sc2**]$_0$/[PLLA]$_0$ = 1/500, 180 °C, 11 h.

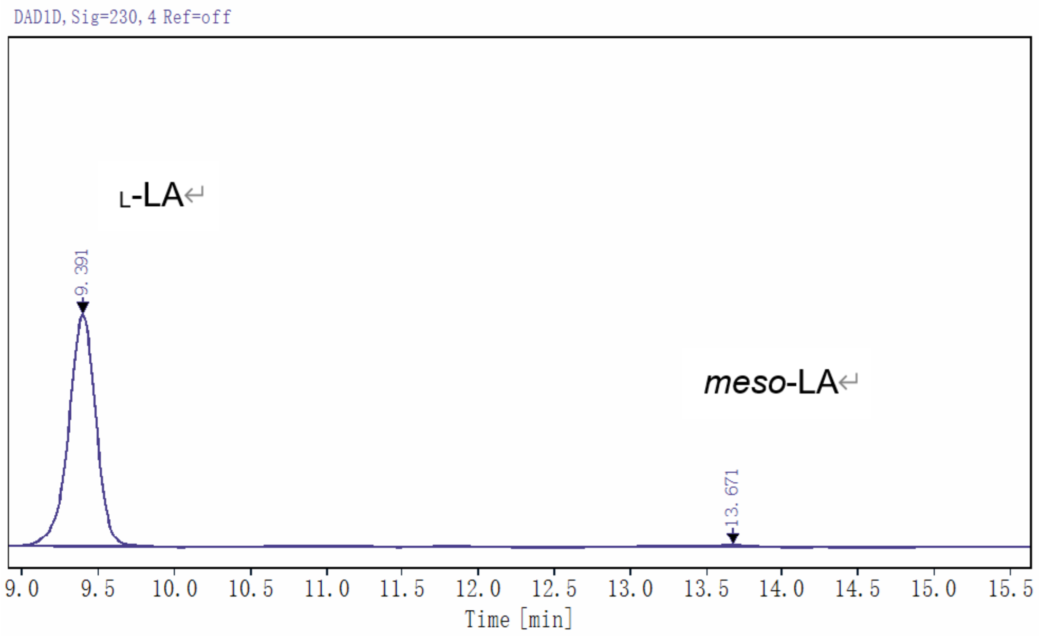

LA	Time (min)	Area	Height	width	Area (%)
L-LA	9.391	0.9404	746.9222	58.8949	99.2720
meso-LA	13.671	0.6251	5.4777	0.3494	0.7280

Fig. S70. HPLC chromatograms of recycled LA, [*rac*-**Sc2**]$_0$/[PLLA]$_0$ = 1/500, 180 °C, 11 h. Column, Chiralpak IA; flow rate, 1 mL min^{-1}; eluent, hexane-isopropanol = 90/10; detector, UV (230 nm); temperature, 35 °C.

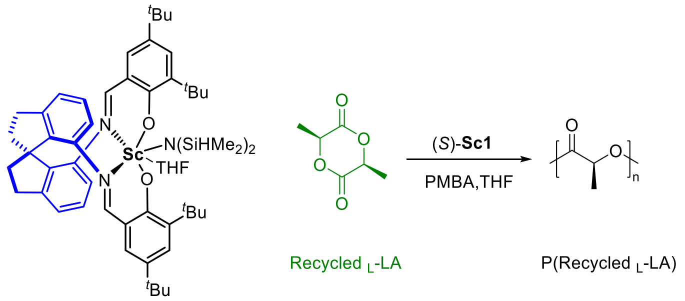

Table S4. Results of re-polymerization of recycled LA by neat thermal chemical recycling.[a]

Entry	*meso*-LA/L-LA	[LA]/[(*S*)-**Sc1**]/[I]	Time (h)	Conv.[b] (%)	M_n[c] (kDa)	$Đ$[c]	T_m[d] (°C)
1[e]	1/99	200:1:1	74	87	20.7	1.30	146/153
2[f]	2/98	200:1:1	58	93	15.9	1.05	146/154
3[g]	2/98	100:1:1	24	97	13.3	1.10	145/153

[a] Condition: The monomer was derived from the bulk thermal chemical recycling of *rac*-**Sc2** at 200 °C, *p*-tolylmethanol as the initiator, THF as the solvent, room temperature. [b] Monomer conversion measured by ^{1}H NMR of the quenched solution. [c] Number-average molecular weight (M_n) and dispersity index ($Đ = M_w/M_n$), determined by size exclusion chromatography (SEC) at 40 °C in THF. [d] Melting temperature (T_m) was measured by DSC with the cooling and heating scan rate of 10 °C min^{-1} for all samples. [e] Recycled L-LA: *rac*-**Sc2** = 0.1%, 200 °C, 11.5 h (Table S3, entry 2). [f] Recycled L-LA: *rac*-**Sc2** = 0.2%, 200 °C, 7.5 h (Table S3, entry 3). [g] Recycled L-LA: *rac*-**Sc2** = 0.2%, 200 °C, 10.5 h (Table S3, entry 4).

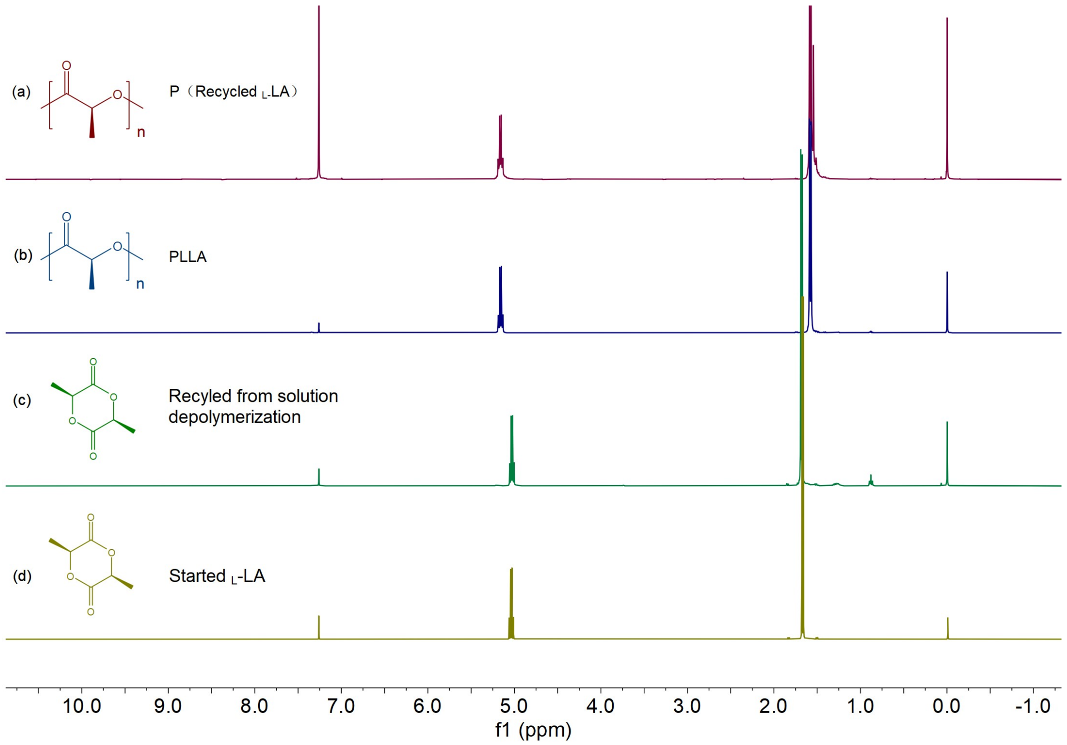

Fig. S71. [1] H NMR ($CDCl_3$, 25 °C) spectra of (a) P(Recycled L-LA) obtained by [Recycled L-LA]/[(*S*)-**Sc1**]/[*p*-tolylmethanol] = 100/1/1 in THF. (b) PLLA obtained by [L-LA]/[Sn(Oct)$_2$]/[BnOH] = 5000/1/50. (c) Recycled L-LA (*rac*-**Sc2** = 0.1%, 200 °C, 11.5 h) by the solution depolymerization. (d) Starting L-LA for comparison.

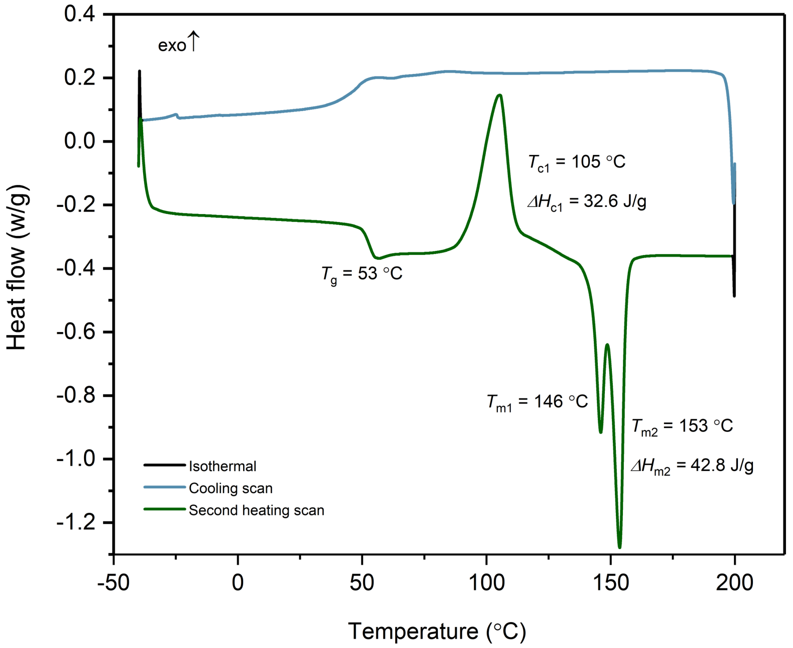

Fig. S72. DSC curves of P(Recycled L-LA) obtained by [Recycled L-LA]/[(*S*)-**Sc1**]/[*p*-tolylmethanol] = 200/1/1, T_m = 146/153 °C.

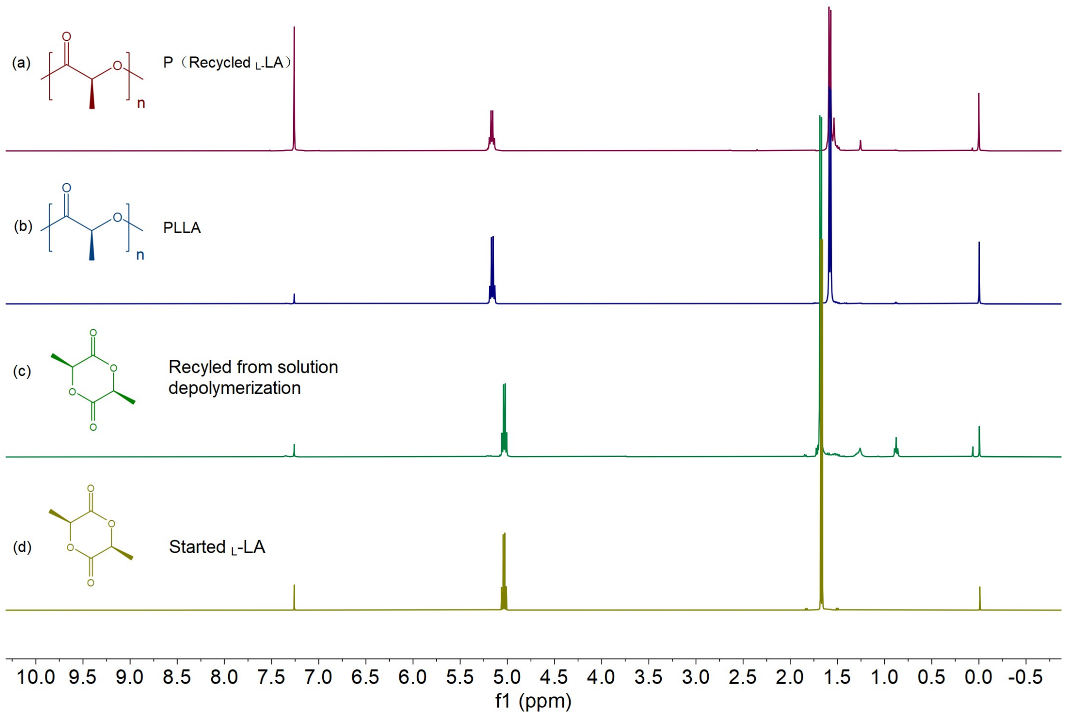

Fig. S73. ^{1}H NMR ($CDCl_3$, 25 °C) spectra of (a) P(Recycled L-LA)obtained by [Recycled L-LA]/[(*S*)-**Sc1**]/[*p*-tolylmethanol] = 200/1/1 in THF. (b) PLLA obtained by [L-LA]/[Sn(Oct)$_2$]/[BnOH] = 5000/1/50. (c) Recycled L-LA (*rac*-**Sc2** = 0.2%, 200 °C, 7.5 h) by the solution depolymerization. (d) Starting L-LA for comparison.

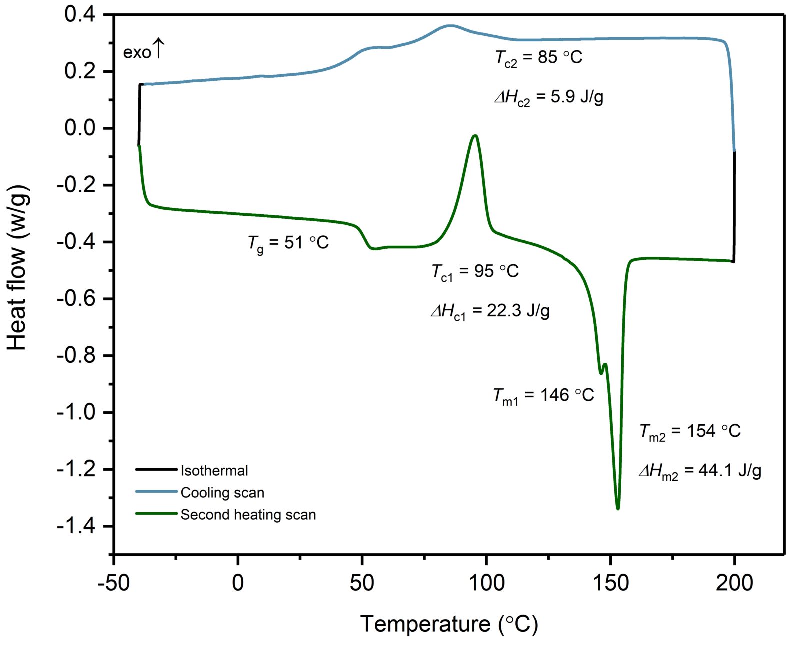

Fig. S74. DSC curves of P(Recycled L-LA)obtained by [Recycled L-LA]/[(*S*)-**Sc1**]/[*p*-tolylmethanol] = 200/1/1, T_m = 146/154 °C.

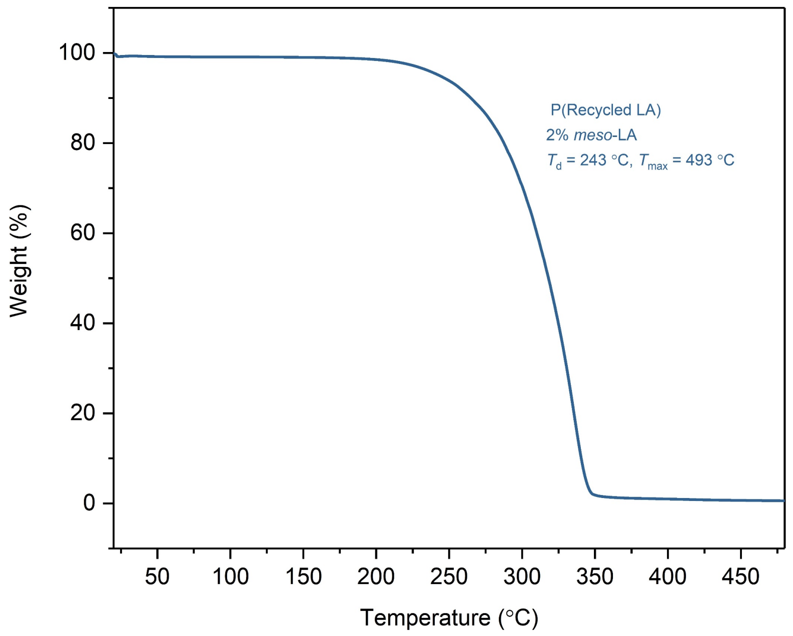

Fig. S75. TGA and DTG curves for P(Recycled L-LA) obtained by [Recycled L-LA]/[(*S*)-**Sc1**]/[*p*-tolylmethanol] = 200/1/1, T_d = 243 °C, T_{max} = 493 °C.

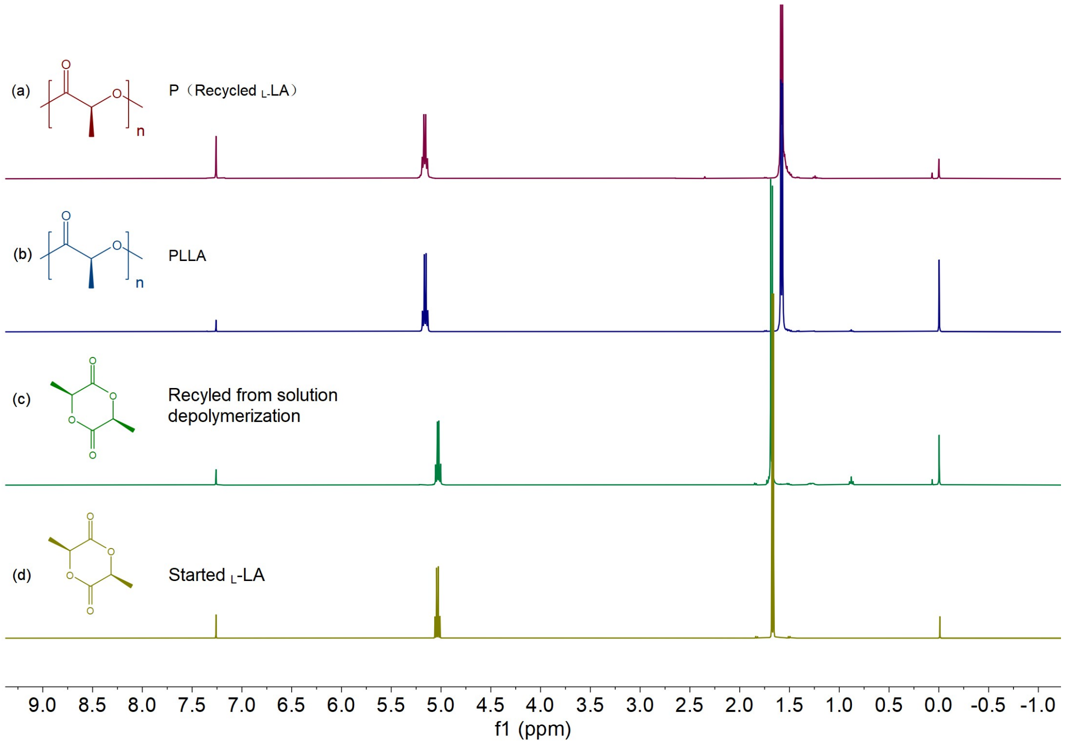

Fig. S76. [1] H NMR ($CDCl_3$, 25°C) spectra of (a) P(Recycled L-LA)obtained by [Recycled L-LA]/[(*S*)-**Sc1**]/[*p*-tolylmethanol] = 100/1/1 in THF. (b) PLLA obtained by [L-LA]/[$Sn(Oct)_2$]/[BnOH] = 5000/1/50. (c) Recycled L-LA(*rac*-**Sc2** = 0.2%, 200 °C, 11.5 h) by the solution depolymerization. (d) Starting L-LA for comparison.

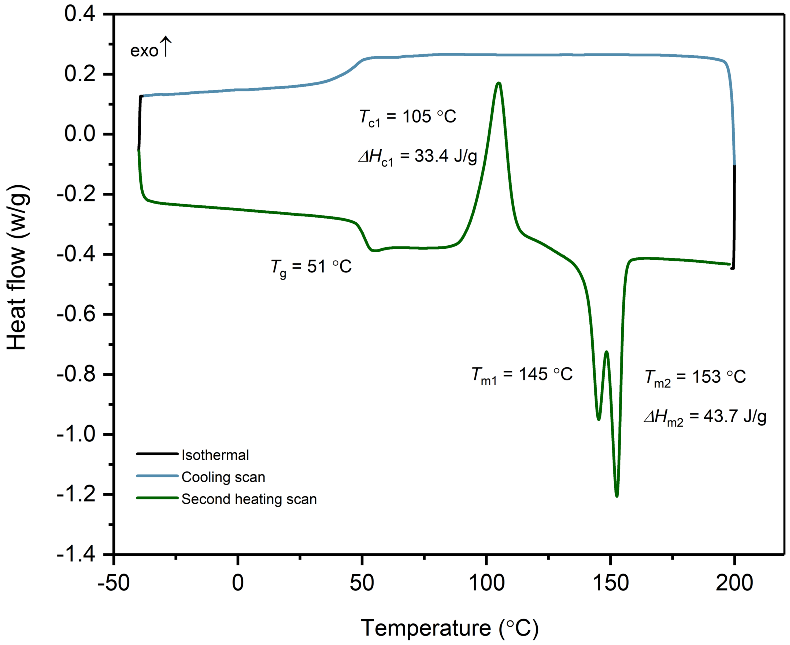

Fig. S77. DSC curves of P(Recycled L-LA)obtained by [Recycled L-LA]/[(*S*)-**Sc1**]/[*p*-tolylmethanol] = 100/1/1, T_m = 145/153 °C

Supplementary References

[1] Trimaille T, Möller M, Gurny R. Synthesis and ring-opening polymerization of new monoalkyl-substituted lactides. *J Polym Sci* 2004; **42:** 4379-4391.

[2] Huang Y-T, Huang H-Y, Cheng J-L *et al.* A Regio- and Stereoselective Ring-Opening Polymerization Approach to Isotactic Alternating Poly(lactic-*co*-glycolic acid) with Stereocomplexation. *Angew Chem Int Ed* 2025; **64:** e202419494.

[3] Chamberlain BM, Cheng M, Moore DR *et al.* Polymerization of Lactide with Zinc and Magnesium β-Diiminate Complexes: Stereocontrol and Mechanism. *J Am Chem Soc* 2001; **123:** 3229-3238.
